# Single-nucleus RNA-Seq reveals singular gene signatures of human ductal cells during adaptation to insulin resistance

**DOI:** 10.1172/jci.insight.153877

**Published:** 2022-08-22

**Authors:** Ercument Dirice, Giorgio Basile, Sevim Kahraman, Danielle Diegisser, Jiang Hu, Rohit N. Kulkarni

**Affiliations:** 1 Islet Cell and Regenerative Biology, Joslin Diabetes Center, Boston, Massachusetts, USA.; 2 Department of Medicine, Beth Israel Deaconess Medical Center, Harvard Medical School, Boston, Massachusetts, USA.; 3 Department of Pharmacology, New York Medical College, Valhalla, New York, USA.; 4 Harvard Stem Cell Institute, Boston, Massachusetts, USA.

**Keywords:** Development, Endocrinology, Beta cells, Islet cells

## Abstract

Adaptation to increased insulin demand is mediated by β cell proliferation and neogenesis, among other mechanisms. Although it is known that pancreatic β cells can arise from ductal progenitors, these observations have been limited mostly to the neonatal period. We have recently reported that the duct is a source of insulin-secreting cells in adult insulin-resistant states. To further explore the signaling pathways underlying the dynamic β cell reserve during insulin resistance, we undertook human islet and duct transplantations under the kidney capsule of immunodeficient NOD/SCID-γ (NSG) mouse models that were pregnant, were insulin-resistant, or had insulin resistance superimposed upon pregnancy (insulin resistance + pregnancy), followed by single-nucleus RNA-Seq (snRNA-Seq) on snap-frozen graft samples. We observed an upregulation of proliferation markers (e.g., *NEAT1*) and expression of islet endocrine cell markers (e.g., *GCG* and *PPY*), as well as mature β cell markers (e.g., *INS*), in transplanted human duct grafts in response to high insulin demand. We also noted downregulation of ductal cell identity genes (e.g., *KRT19* and *ONECUT2*) coupled with upregulation of β cell development and insulin signaling pathways. These results indicate that subsets of ductal cells are able to gain β cell identity and reflect a form of compensation during the adaptation to insulin resistance in both physiological and pathological states.

## Graphical abstract

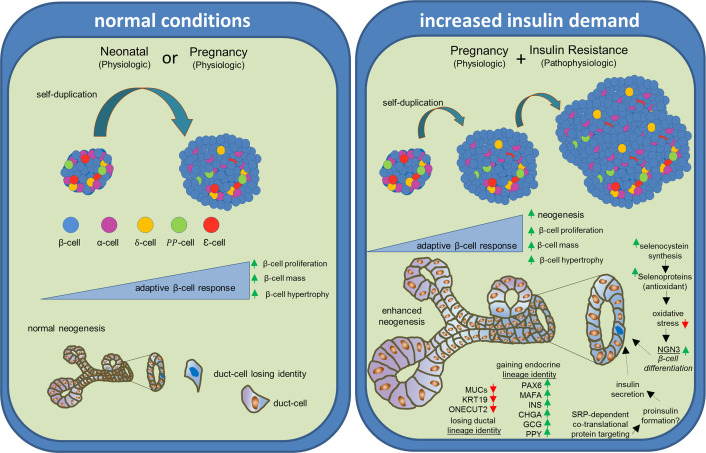

## Introduction

Compensation for increased insulin demand includes key mechanisms ranging from either an increase in insulin secretion, expansion in β cell mass, or both. The latter occurs as a consequence of the balance between cell growth (proliferation, hypertrophy, neogenesis) and cell loss (apoptosis, atrophy, autophagy) (1–7). Typically, the inability of β cells to successfully handle the increased demand for insulin triggers overt diabetes mellitus. Therefore, understanding the mechanisms of this compensatory response will inform potential approaches to therapeutically enhance functional β cell mass to counter all forms of diabetes.

In addition to proliferation of preexisting β cells (8), other mechanisms such as β cell hypertrophy and neogenesis have also been reported to contribute to β cell compensation in rodents (3). Among these mechanisms, neogenesis, described as the differentiation of progenitors to form new β cells (9), has been reported to occur in the neonatal period (10). However, several reports, including a study from our group (7), argue for neogenesis as a contributor to β cell compensation even in adult mammals (mouse and human) in the face of increased insulin demand (3). In our previous study, we superimposed pregnancy to further increase insulin demand in a model that already exhibits an increase in β cell mass, such as the unique genetically engineered liver-specific insulin receptor-KO (LIRKO) mouse, to demonstrate that pancreatic ducts are a dynamic source of insulin-secreting cells (7).

Here, we explored the mechanisms underlying the contribution of the ductal epithelium to adaptive β cell mass, using single-nucleus RNA-Seq (snRNA-Seq), a technique we recently optimized as a reliable alternative when single-cell RNA-Seq (scRNA-Seq) is not suitable to examine archived frozen human islet grafts (11). In the current study, we undertook snRNA-Seq of snap-frozen human islet and duct graft samples obtained from nonpregnant, pregnant control, or genetically engineered insulin-resistant mice (control NOD/SCID-γ–Lox [NSG-Lox] or genetically engineered insulin-resistant NSG-LIRKO, respectively, described below), to analyze the effects of pregnancy, insulin resistance, or a combination of pregnancy and insulin resistance — the latter hereinafter referred to as “combined model.” snRNA-Seq analyses of human ductal clusters revealed unique gene signatures, including the presence of mature and immature β cell markers, poly-hormonal islet endocrine cell markers, an upregulation in β cell development, and insulin signaling pathways coupled with downregulation of markers of ductal cell identity together supporting neogenesis. These data provide genetic evidence for the duct as a source of β/β-like cells in the compensatory response to insulin resistance.

## Results

### snRNA-Seq reveals insulin-related transcriptional diversity among clusters.

**Figure 1:**
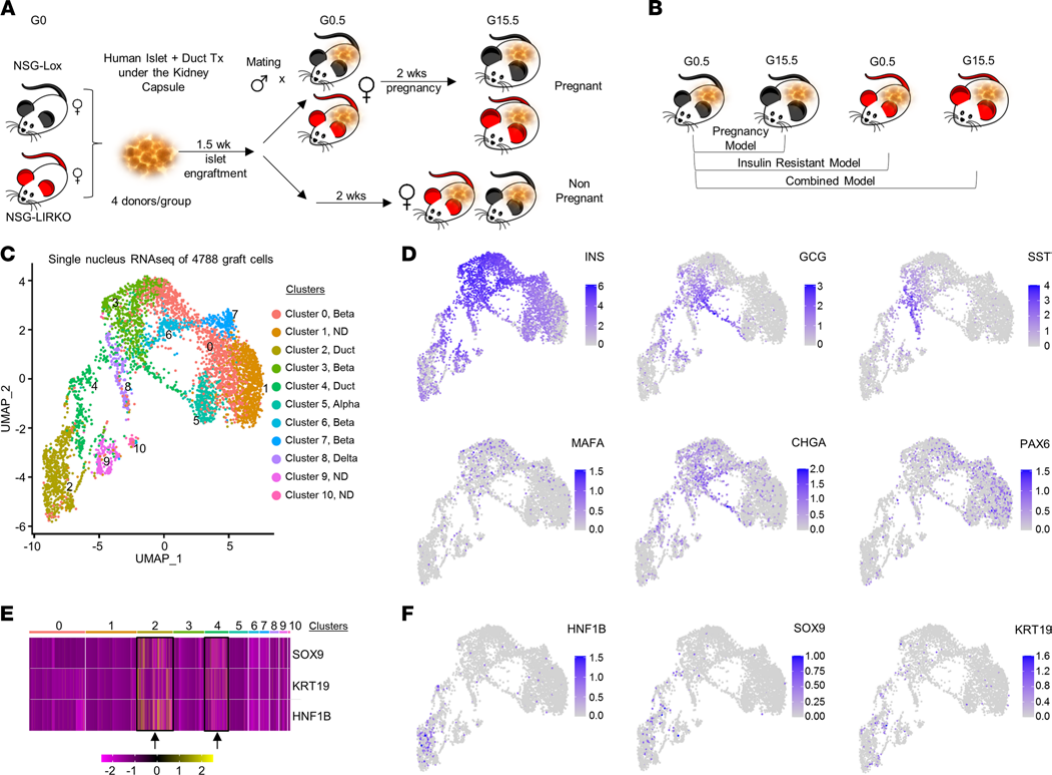
Single-nucleus RNA-Seq reveals presence of insulin and glucagon double-positive ductal cells. (**A** and **B**) Experimental strategy and the 3 experimental groups (pregnancy model, insulin-resistant model, and combined [insulin resistance + pregnancy] model) showing female NSG-Lox (black) and NSG-LIRKO (red) mice transplanted with human islets (1000 IEQs) and duct aggregates (obtained from the same donor; *n* = 4 donors) under the kidney capsule. Ten days after transplantation, mice were rendered pregnant and sacrificed on gestation day 15.5 (G15.5) for collection of human grafts. Nonpregnant female mice transplanted with human islets and ducts were used as controls. The experimental groups include nonpregnant NSG-Lox (NP NSG-Lox, lean back mice, *n* = 4), pregnant NSG-Lox mice (P NSG-Lox, wide black mice, *n* = 4), nonpregnant NSG-LIRKO (NP NSG-LIRKO, lean red mice, *n* = 4), and pregnant NSG-LIRKO (P NSG-LIRKO, wide red mice, *n* = 4). The effect of pregnancy was evaluated by comparing pregnant NSG-Lox mice (P NSG-Lox) with the nonpregnant NSG-Lox animals (NP NSG-Lox) and defined as pregnancy model. The effect of insulin resistance was determined by comparing nonpregnant NSG-LRKO mice (NP NSG-LIRKO) with nonpregnant NSG-Lox mice (NP NSG-Lox) and defined as insulin-resistant model. The effect of insulin resistance + pregnancy was considered a combined model and evaluated by comparing pregnant NSG-LIRKO models (P NSG-LIRKO) with nonpregnant NSG-Lox mice (NP NSG-Lox). (**C**) Global UMAP plot of 4788 profiled nuclei colored by the 11 clusters. Clusters were identified according to the expression patterns of the endocrine and exocrine cell marker genes. (**D**) Global UMAP plot showing expression of indicated gene markers for different endocrine cells. (**E**) Selected heatmap showing normalized expression of ductal cell markers (*HNF1B*, *KRT19*, and *SOX9*) within all the identified nuclear clusters of 4 different mouse models: nonpregnant (NP) NSG-Lox, pregnant (P) NSG-Lox, NP NSG-LIRKO, and P NSG-LIRKO. (**F**) Global UMAP showing expression of indicated markers for ductal cells.

To identify the origin of new insulin-expressing cells during increased insulin demand, we followed up on a model that was created to study human ductal epithelium–derived β/β-like cells (7). Briefly, we compared control (NSG-Lox) with genetically engineered insulin-resistant (NSG-LIRKO) mice (Figure 1, A and B). Both groups were rendered pregnant following transplantation of 100 human ductal “aggregates,” along with 1000 human islet equivalents (IEQs) from the same donor (for donor information, see Supplemental Table 1; supplemental material available online with this article; https://doi.org/10.1172/jci.insight.153877DS1). After 15.5 days, the grafts were harvested from mice in nonpregnant or pregnant states and stored frozen until analyses. One half of the frozen engrafted human islet and duct samples were subsequently thawed and processed to isolate nuclei for snRNA-Seq, as reported previously (11). We began analyses by using Cellbender (https://doi.org/10.1101/791699) and Doubletfinder (12) algorithms to eliminate the contribution of contaminant RNA and multiplets (i.e., beads containing > 1 nucleus), respectively, from the sequencing outputs, as previously described (11). The proportion of ambient RNA in the beads was estimated to be ~14% in all groups and was, therefore, excluded from subsequent analyses (Supplemental Figure 1). This enabled only high-quality nuclei to be used for cell-clustering analysis. Profiling of human islet and ductal cell grafts was performed on data from snRNA-Seq (4788 nuclei) using Uniform Manifold Approximation and Protection (UMAP), a dimension-reduction technique used to cluster cells/nuclei that show similar transcriptional profiles (13) (Figure 1C). We identified a total of 11 clusters (numbered 0–10) composed of islet endocrine and nonendocrine cells (Figure 1C). In particular, we observed nuclei expressing high levels of insulin gene (*INS*) (log_2_CPM [count per million] ≥ 5) localized to cluster 3 (Figure 1, C and D). Mature markers that define β cells, such as MAF bZIP transcription factor A (*MAFA*) or chromogranin A (*CHGA*), were expressed exclusively in cluster 3 (Figure 1, C and D). High-level expression of glucagon gene (*GCG*), the hormone expressed by α cells, was observed in clusters 3 and 4 (log_2_CPM ≥ 2) (Figure 1, C and D). Nevertheless, *INS* and *GCG* transcripts were also detected, albeit at lower levels, in nuclei grouped in additional clusters (Figure 1, C and D). For example, low levels of *INS* (log_2_CPM < 5) were detected in clusters 0, 4, 7, 8, 9, and 10, while low expression of *GCG* (log_2_CPM < 2) were observed in clusters 6, 8, and 9 (Figure 1, C and D), suggesting the presence of transcripts in poly-hormonal states. These data demonstrate that snRNA-Seq of transplanted human islets and ductal aggregates recapitulate the expression of islet endocrine cells.

### Ductal cells exhibit insulin^+^ or insulin/glucagon double-positive expression.

**Figure 2:**
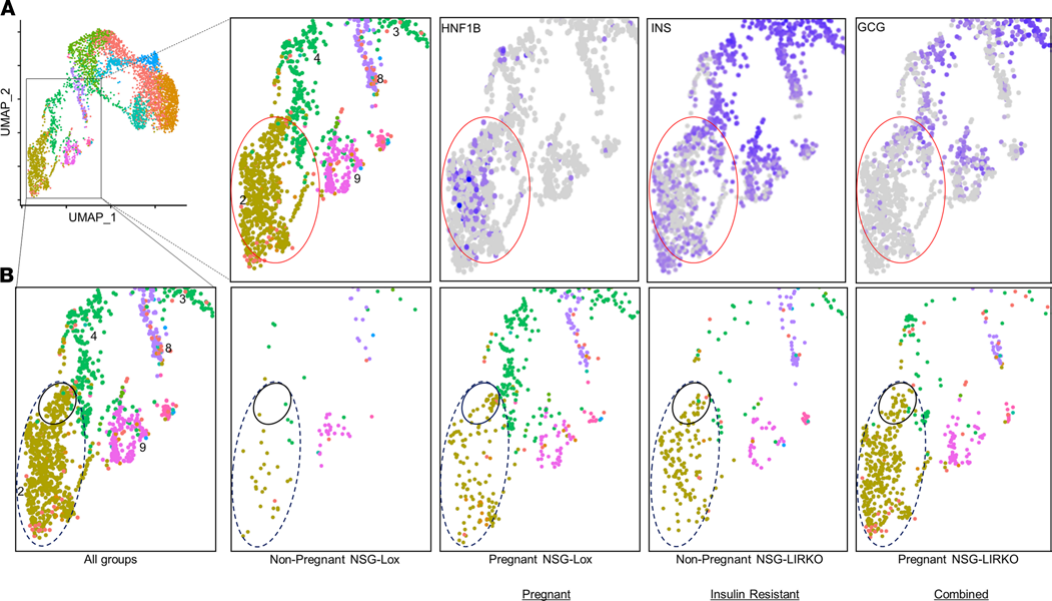
Identification of a specific ductal subgroup in response to increased insulin demand. (**A**) Expression levels of *HNF1B*, *INS*, and *GCG* in ductal nuclear cluster 2 (red oval), ranging from high expression (purple dots) to low expression levels (gray dots). (**B**) UMAP plot showing ductal nuclear cluster 2 (yellow dots inside the blue-dotted oval) from human graft samples of all the 4 experimental groups (NP NSG-Lox, P NSG-Lox, NP NSG-LIRKO, and P NSG-LIRKO). The black solid circle within the blue dotted oval represents a ductal subpopulation.

Moving forward, we focused our studies on clusters defined as ductal cells and used HNF1 homeobox B (*HNF1B*), keratin 19 (*KRT19*), and SRY-box transcription factor 9 (*SOX9*) as ductal marker genes (14). We identified cluster 2 as a major ductal cluster, with nuclei expressing relatively high levels of ductal markers (*HNF1B* log_2_CPM ≥ 1, *KRT19* log_2_CPM ≥ 1, and *SOX9* log_2_CPM ≥ 1), and we identified cluster 4 as a partial ductal cluster, including specimens with relatively lower expression of duct-specific genes (*HNF1B* log_2_CPM ≤ 1, *KRT19* log_2_CPM ≤ 1, and *SOX9* log_2_CPM ≤ 1) (Figure 1, C, E, and F). Detailed analysis of cluster 2 revealed the presence of ductal nuclei expressing both insulin and glucagon (Figure 2A).

A notable observation was that *GCG* expression in ductal clusters 2 and 4 could be divided into subgroups suggesting heterogeneity (Figure 2A). These results prompted us to search for ductal cell groups that are a potential source of insulin-secreting β/β-like cells. Indeed, analysis of individual human grafts from the mouse models (pregnant, insulin-resistant, or combined groups) further supported our premise regarding subgroups among ductal cells. For example, a specific ductal subpopulation enriched in *INS*/*GCG* double-positive nuclei within cluster 2, was evident in the pregnant, insulin-resistant, or combined models (Figure 2B; small black circle within the dotted oval, and Supplemental Figure 2; shown with red arrows). Furthermore, confocal and fluorescence microscopy analyses of kidney sections of the transplanted grafts containing human ducts and islets revealed cells coexpressing the ductal marker CK19 and insulin (Supplemental Figure 3A and Supplemental Figure 4, A–D), CK19 and glucagon (Supplemental Figure 3B), or insulin and glucagon (Supplemental Figure 3C). Notably, we did not detect groups of nuclei, which expressed *CHGA*, *MAFA*, and *PAX6* (Figure 1D), in the human islet and duct graft samples from the nonpregnant NSG-Lox model (Figure 2B). Taken together, these data suggest that a specific subset of ductal cells has the potential to be mobilized to differentiate toward β-like cells only during increased insulin demand either in physiological or pathological states.

**Figure 3:**
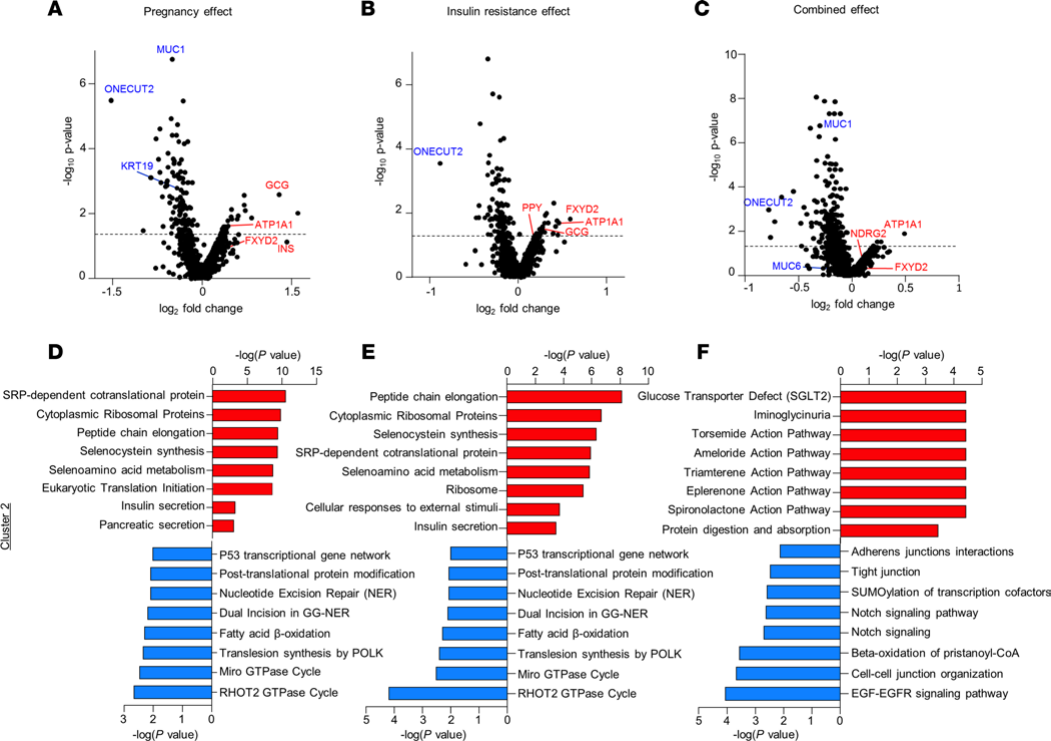
Ductal clusters exhibit regulation in pathways related to β cell development and ECM remodeling in response to pregnancy and insulin resistance. (**A**–**C**) Volcano plots showing the distribution of differential transcript expression defined as a function of fold change within the pregnancy (P NSG-Lox versus NP NSG-Lox) (**A**), insulin-resistant (NP NSG-LIRKO versus NP NSG-Lox) (**B**), or combined (P NSG-LIRKO versus NP NSG-Lox) models and *P* value for ductal cluster 2 (**C**). (**D**–**F**) Selected pathways in ductal cluster 2 differentially regulated in pregnancy (P NSG-Lox versus NP NSG-Lox) (**D**), insulin-resistant (NP NSG-LIRKO versus NP NSG-Lox) (**E**), or combined (P NSG-LIRKO versus NP NSG-Lox) models (**F**). Upregulated pathways are shown in red, and downregulated pathways are shown in blue.

We next focused on identifying differentially expressed genes (*P* < 0.05) within the major and the partial ductal clusters, namely clusters 2 and 4, respectively. We observed enrichment of the α cell hormone *GCG* in the major ductal nuclear cluster (cluster 2) in the pregnancy model and *GCG* and *PPY* in the insulin-resistant model (Figure 3, A and B). Moreover, the ATPase Na^+^/K^+^ transporting subunit α 1 (*ATP1A1*) was one of the top enriched genes in all 3 groups (Figure 3, A–C). *ATP1A1* is an integral membrane protein regulating the electrochemical gradients of Na^+^ and K^+^ ions across the plasma membrane and is upregulated in PAX6-deficient β cells (15). The FXYD domain containing ion transport regulator 2 (*FXYD2*), which has been reported to play a role in β cell growth and proliferation (16), was significantly enriched in the insulin-resistant model in cluster 2 and in the pregnancy model in cluster 4 (Figure 3B and Supplemental Figure 5A). In the ductal cluster 2, we observed an enrichment for the N-myc downstream-regulated gene 2 (*NDRG2*) in the combined group (Figure 3C). *NDRG2* is highly expressed in β cells and is reported to be involved in Akt-mediated protection against lipotoxicity (17).

Among the downregulated genes, One Cut Homeobox 2 (*ONECUT2*), a ductal cell–specific transcription factor (18), was one of the top genes in all 3 groups in cluster 2 (Figure 3, A–C). Besides, the ductal marker *KRT19* was also downregulated in the pregnancy model. Mucin 1 (*MUC1*), a protein involved in cell adhesion that was previously reported as a subductal cell gene marker (19), was significantly reduced in cluster 2 in the pregnancy and combined models (Figure 3A). These results suggest that, during pregnancy and insulin-resistant states, a specific group of cells gain the identity of islet endocrine cells at the expense of the duct epithelium to orchestrate the compensatory response to increased insulin demand.

The significant increase in the number of proliferating ductal cells during increased insulin demand in our previous study (7) prompted us to carefully interrogate the ductal clusters. The long noncoding RNA (lncRNA) nuclear enriched abundant transcript 1 (*NEAT1*), which was reported to promote proliferation and migration in cancer progression (20, 21), was significantly increased in ductal nuclei grouped in cluster 4 in the combined model (Supplemental Figure 5, B and C). Further work is necessary to directly examine its significance in modulating human ductal cell proliferation.

### Similar pathways are activated in ductal cells in response to pregnancy and insulin resistance.

Transcriptomics analyses revealed a similar network of molecules and/or pathways that are differentially regulated during increased insulin demand in the pregnancy or insulin-resistant models. Analyses of the major ductal clusters 2 and 4 revealed that translation and secretion-related pathways (Figure 3, D and E, and Supplemental Tables 2 and 3) overlapped between the pregnancy and the insulin-resistant models. These included the “selenocysteine synthesis” pathway and the signal recognition peptide–dependent (SRP-dependent) co-translational protein targeting to the membrane pathway, which emerged as the most upregulated in both clusters 2 and 4 in the pregnancy and the insulin-resistant models (Figure 3, D and E, and Supplemental Tables 2 and 3). A majority of the differentially regulated transcripts in both of these pathways were ribosomal proteins. Not surprisingly, insulin secretion and pancreatic secretion pathways were upregulated in the pregnancy and insulin-resistant models that was reflected in both ductal clusters 2 and 4 (Figure 3, D and E; Supplemental Figure 5, D–F; and Supplemental Tables 2, 3, 5, and 6) consistent with an upregulation in insulin receptor gene (*INSR*) in ductal cluster 4 in the pregnant NSG-LIRKO versus nonpregnant NSG-Lox comparison (Supplemental Figure 5C and Supplemental Table 7), suggesting that changes in secretion are part of the adaptive response. Compared with individual models of increased insulin demand (pregnancy and insulin-resistant models), the “spironolactone action pathway” associated with improved glucose and lipid metabolism was one of the upregulated mechanisms in the combined model (Figure 3F).

Among the downregulated pathways, signaling pathways related to lipid metabolism (e.g., fatty acid β-oxidation, ceramide signaling) were common to all the models in cluster 2 (Figure 3, D–F, and Supplemental Tables 2–4). In addition, the insulin-resistant and combined models from nuclei in cluster 2, and the pregnancy model from nuclei in cluster 4, displayed downregulation of pathways linked to Notch signaling, a pathway typically active in exocrine tissues (22) (Figure 3, E and F; Supplemental Figure 5D; and Supplemental Tables 3–5). Cell–extracellular matrix (cell-ECM) and cell-to-cell communication pathways, whose downregulation is usually observed during ductal–to–β cell transdifferentiation (23), were found repressed in ductal cluster 2 in the combined model and in ductal cluster 4 in the pregnancy and insulin-resistant models (Figure 3F, Supplemental Figure 5D, and Supplemental Tables 4 and 5). Thus, a subset of cells in specific clusters of the ductal cell population is linked to key pathways that are relevant for β cell development and hormone secretion — 2 processes that are important for an efficient adaptive response to insulin resistance.

### Ductal clusters exposed to high insulin demand display transcriptomic similarities with endocrine progenitor cells.

**Figure 4:**
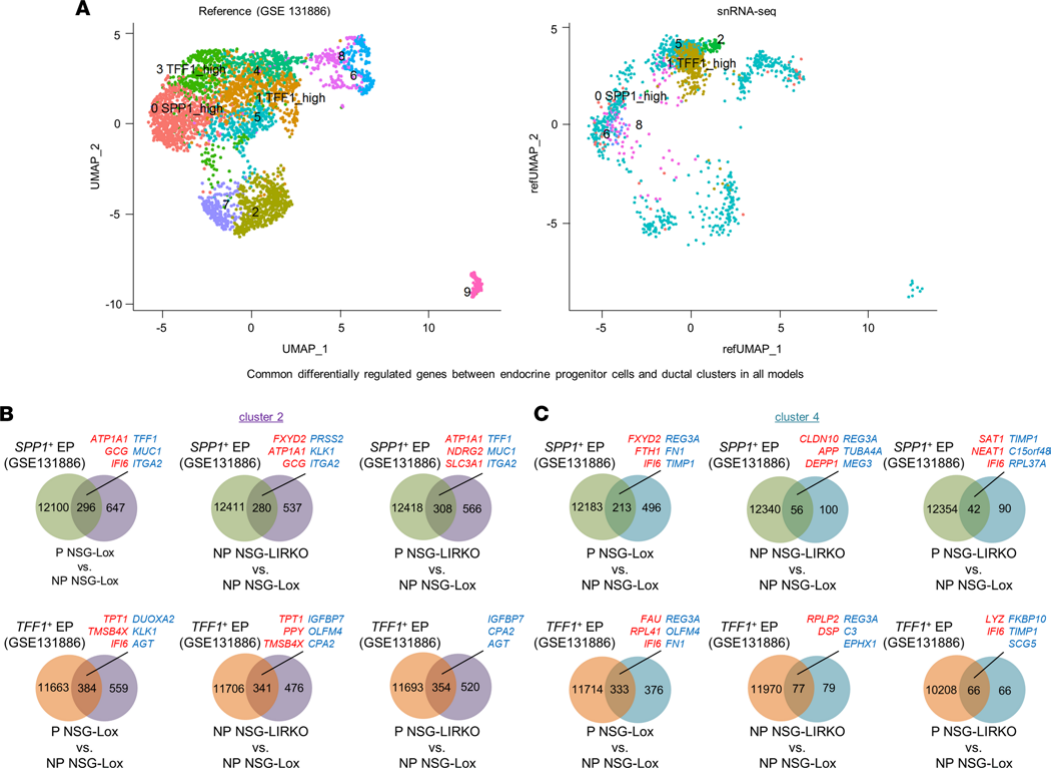
The transcriptomic profile of ductal cells in conditions of high insulin demand resemble the gene expression profile of endocrine progenitor cells. (**A**) Global UMAP plots and cell type prediction in the engrafted human ductal and islet cell snRNA-seq (right panel) following harmonization on the reference data set (GSE131886) (24) generated from the scRNA-seq data sets of cultured human ductal cells (left panel). (**B** and **C**) Venn diagrams representing the intersection between the significant differentially expressed genes in *SPP1*^+^ (green circles) or *TFF1*^+^ cells (orange circles) and in the ductal cluster 2 (purple circles) (**B**) or 2 (aquamarine circles) (**C**) in pregnancy (P NSG-Lox versus NP NSG-Lox, left panels), insulin-resistant (NP NSG-LIRKO versus NP NSG-Lox, middle panels), or combined (P NSG-LIRKO versus NP NSG-Lox, right panels) models. The common upregulated genes are written in red, and the downregulated genes are written in blue.

To test whether the transcriptomic signature of ductal cells transplanted into mouse models exhibiting high insulin demand actually resembled the gene expression profile of endocrine progenitor cells, we compared the 2 ductal clusters (clusters 2 and 4) with ductal endocrine progenitor cells reported in a previous scRNA-Seq study (GSE131886) (24). The endocrine progenitor cells in the latter were grouped into 2 main clusters: (a) Secreted Phosphoprotein 1 (*SPP1*)^+^ cells, designated as harboring endocrine progenitor-like cells, and (b) Trefoil Factor 1 (*TFF1*)^+^ cells, defined as activated/migrating endocrine cells (24). By harmonizing our snRNA-Seq data set on the GSE131886 output (designated as “reference”), and using shared pipelines reported previously (11), we identified 2 nuclear clusters within our own data set, based on transcriptomic features similar to the *TFF1*^+^ and the *SPP1*^+^ cells from the reference (Figure 4A). We identified common gene signatures by intersecting data sets between nuclear clusters 2 and 4 and the *TFF1*^+^ and *SPP1*^+^ endocrine progenitor cells (Figure 4, B and C). For example, we observed that ductal cluster 2 in the pregnancy, insulin-resistant, or combined models shared 31.4% (296 of 943 genes), 34.3% (280 of 817 genes), and 35.2% (308 of 874 genes), respectively, of the differentially regulated genes with the *SPP1*^+^ endocrine progenitor-like cells (Figure 4B). Slightly higher proportions of differentially expressed genes were common between *TFF1*^+^ progenitor-like cells and cluster 2 in pregnancy (40.7%, 384 of 943 genes), insulin-resistant (41.7%, 341 of 817 genes), or combined models (40.5%, 354 of 874 genes) (Figure 4B). On the other hand, the percentage of genes that were common between nuclear ductal cluster 4 and *SPP1*^+^ cells were 30.0% (213 of 709 genes), 35.9% (56 of 156 genes), and 31.8% (42 of 132 genes), in pregnancy, insulin-resistant, and combined models, respectively (Figure 4C). Finally, cluster 4 shared 46.9% (333 of 709 genes), 49.3% (77 of 156 genes), 50.0% (66 of 132 genes) of differentially regulated genes in the pregnancy, insulin-resistant, or combined models, respectively, with *TFF1*^+^ cells (Figure 4C).

The upregulation of islet cell genes in nuclei within cluster 2, such as *GCG* in pregnancy or insulin-resistant models, or *PPY* in the insulin-resistant model, in common with *SPP1*^+^ and *TFF1*^+^ cells, respectively, suggested cells are transitioning toward an endocrine cell phenotype (Figure 4B). In addition, *FXYD2* and ferritin heavy chain 1 (*FTH1*), 2 genes recently identified as β cell specific (18, 25), were upregulated in clusters 2 and 4 in different models and shared with *SPP1*^+^ progenitor cells (Figure 4, B and C). Among those that were downregulated, *MUC1*, integrin subunit α 2 (*ITGA2*), and fibronectin (*FN1*) — which regulate cell adhesion and cell-ECM interactions — were shared by the 2 ductal nuclear clusters 2 and 4 in the different models and the 2 types (*TFF1*^+^ and *SPP1*^+^) of endocrine progenitor cells (Figure 4, B and C). These comparative analyses indicate that ductal cells adapt to an insulin-resistant environment by adopting a transcriptomic profile that resembles typical endocrine progenitor cells.

### T2D human β cells reveal regulation of pathways that are also present in ductal cells in response to pregnancy and insulin resistance.

**Figure 5:**
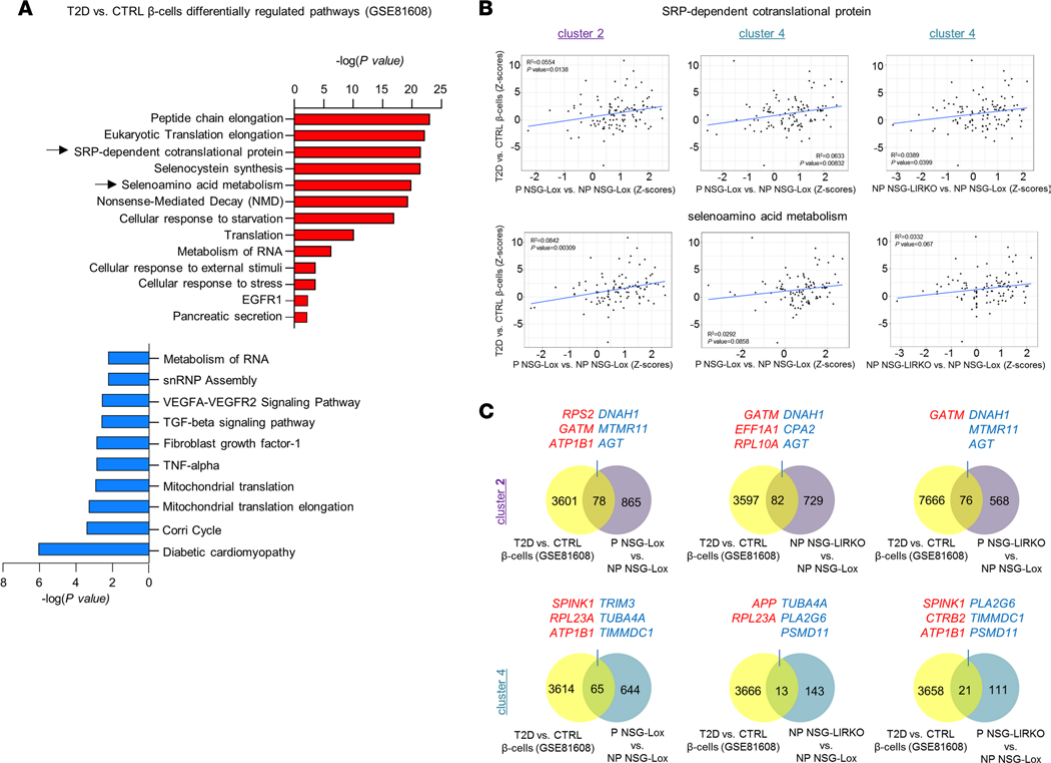
Ductal cells in models of insulin resistance display regulated pathways and genes similar to T2D human β cells. (**A**) Selected pathways derived from reanalysis of the publicly available data set (GSE81608) (26) comparing nondiabetic (CTRL) and T2D human β cells. Arrowheads highlight common pathways activated in ductal cells in pregnancy (P NSG-Lox versus NP NSG-Lox), insulin-resistant (NP NSG-LIRKO versus NP NSG-Lox), or combined models (P NSG-LIRKO versus NP NSG-Lox). Red bars indicate upregulated pathways, and blue bars indicate downregulated pathways ordered by –log_10_ (*P* value; *x* axis) (**B**) Linear regression analysis of expression levels (measured as *Z* scores) of genes related to the SRP-dependent cotranslational protein pathway (top panels) and the Selenoamino acid metabolism pathway (bottom panels) in T2D versus CTRL human β cells (*y* axis) and ductal cluster 2 (left panels) or 4 (middle and right panels) in pregnancy (P NSG-Lox versus NP NSG-Lox) or insulin-resistant (NP NSG-LIRKO versus NP NSG-Lox) models. (**C**) Venn diagrams representing the intersection between the significant differentially expressed genes in T2D versus CTRL human β cells (yellow circles) and the ductal cluster 2 (purple circles, top panels) or 4 (aquamarine circles, bottom panels) in pregnancy (P NSG-Lox versus NP NSG-Lox left panels), insulin-resistant (NP NSG-LIRKO versus NP NSG-Lox, middle panels), or combined (P NSG-LIRKO versus NP NSG-Lox, right panels) models. The upregulated genes are written in red, and the downregulated genes are written in blue.

Finally, to examine whether patients with T2D who potentially exhibit an adaptive response to enhanced insulin demand exhibit similar pathways in their β cells, we compared our results with single-cell data sets in the public domain. Reanalyses of the scRNA-Seq GSE81608 data set comparing nondiabetic versus T2D human islets (26), showed that “selenocysteine synthesis,” “selenoamino acid metabolism,” and “SRP-dependent co-translational protein targeting to the membrane” pathways were all upregulated in β cells from the latter. Notably, these pathways were also identified in ductal cells (cluster 2 and 4) in the pregnancy and insulin-resistant models (Figure 5A and Supplemental Table 8). Common pathways between T2D β cells and ductal clusters in insulin-resistant states were a feature among the downregulated ones (Figure 5A and Supplemental Table 8). Notably, pathways involved in inflammatory processes, such as TNF-α and TGF-β, were suppressed in ductal clusters from grafts transplanted in mice with insulin resistance, as well as in T2D β cells in comparison with their respective controls (CTRL) (Figure 5A and Supplemental Table 8).

To explore the relevance of the “selenoamino acid metabolism” and the “SRP-dependent cotranslational protein targeting to the membrane” pathways, we performed linear regression to assess the correlation of gene expression between T2D β cells and the ductal clusters in the 3 models (pregnancy, insulin-resistant, and combined) (Figure 5B). A positive and significant correlation of the expression of genes involved in the “SRP-dependent cotranslational protein targeting to the membrane” pathway and the selenocysteine signaling was observed between T2D versus CTRL β cells and clusters 2 and 4 in the pregnancy model. Similar positive associations were found between T2D versus CTRL β cells and cluster 4 in the insulin-resistant model. Together, these data emphasize the importance of these pathways in cells transitioning toward an endocrine identity when subjected to high insulin demand.

To determine the similarities in terms of global gene expression between insulin-resistant β cells and ductal clusters subjected to high insulin demand, we intersected the differentially regulated genes in T2D versus CTRL β cells from the GSE81608 public data set with the differentially regulated genes in ductal cluster 2 and cluster 4 in each of the 3 experimental models from the snRNA-Seq outputs (Figure 5C). We observed 78, 82, and 76 differentially expressed genes that were common between the T2D versus CTRL β cells and ductal cluster 2 in the pregnancy, insulin-resistant, or combined models, respectively (Figure 5C). Among these 3 sets, we identified genes involved in ribosomal complex (*RPS2*, *RPL10A*), metabolism (*GATM*, *ATP1B1*), and exocrine pancreas function (*CPA2*). Upon intersecting the T2D versus CTRL β cells with the ductal cluster 4, we identified 65, 13, and 21 significantly dysregulated genes that were shared with pregnancy, insulin-resistant, or combined models, respectively (Figure 5C). These included genes coding for ribosomal protein (*RPL23A*) and genes important for microtubule assembling (*TUBA4A*), in addition to genes related to exocrine function (*CTRB2*, *SPINK1*). These data suggest that models of physiological (pregnancy) or pathological insulin resistance express a specific phenotype of ductal cells that are also evident in β cells from insulin-resistant T2D patients.

### The “Selenocysteine synthesis”and the “SRP-dependent cotranslational protein targeting to the membrane signaling” pathways are potentially involved in driving the endocrine lineage in ductal cells in response to insulin resistance.

**Figure 6:**
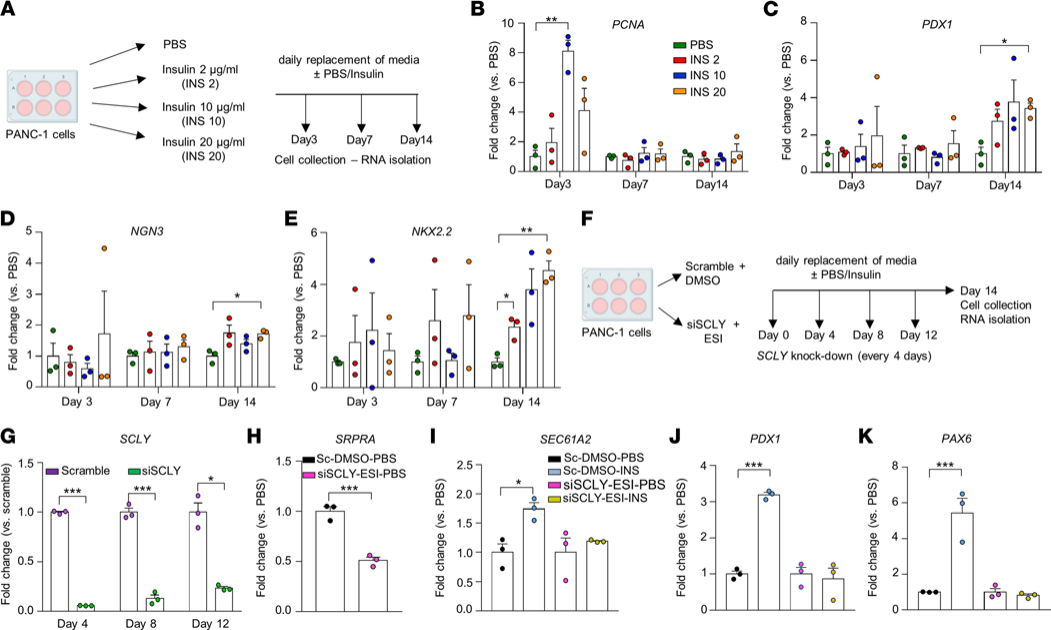
The “selenocysteine synthesis” and the “SRP-dependent co-translational protein targeting to the membrane” pathways modulate endocrine cell gene expression in ductal cells treated with insulin. (**A**) Scheme of the insulin treatment optimization experiment. (**B**–**E**) Expression levels of *PCNA* (**B**), *NGN*-3 (**C**), *PDX1* (**D**), and *NKX2.2* (**E**) in PANC-1 cells treated every 24 hours with either PBS (black bars) or human insulin at 2 (pink), 10 (red) or 20 μg/mL (dark red) for 3, 7, or 14 days. Data are represented as mean of fold change compared with PBS-treated cells ± SEM (*n* = 3). *P* < 0.05 was considered as significant using 2-way ANOVA following Dunnet’s multiple-comparison adjustment. (**F**) Scheme of the selenocysteine synthesis and SRP pathway inhibition experiment. (**G**) *SCLY* expression levels in scramble (green bars) and siSCLY PANC-1 cells (orange bars) at different time points. Data are represented as mean of fold change compared with scramble cells ± SEM (*n* = 3). *P* < 0.05 was considered as significant using 2-way ANOVA following Bonferroni’s multiple comparison adjustment. (**H**) *SRPRA* expression levels in scramble cells treated every 24 hours with DMSO + PBS (sc-DMSO-PBS, black) and siSCLY cells treated with ESI + PBS (siSCLY-ESI-PBS, blue). Data are represented as mean of fold change compared with PBS-treated cells ± SEM (*n* = 3). *P* < 0.05 was considered as significant using unpaired 2-tailed *t* test. (**I**–**K**) Expression levels of *SEC61A2* (**I**), *PDX1* (**J**), and *PAX6* (**K**) in sc-DMSO-PBS cells (black), sc-DMSO cells treated with insulin (sc-DMSO-INS, red), siSCLY-ESI-PBS cells (blue), or siSCLY-ESI cells treated with insulin (siSCLY-ESI-INS, purple). Data are represented as mean of fold change compared with their respective PBS-treated cells ± SEM (*n* = 3). *P* < 0.05 was considered as significant using 1-way ANOVA following Bonferroni’s multiple-comparison test. Data are expressed as mean ± SEM. *P < 0.05, **P < 0.01, and ***P < 0.001.

The pathway analyses performed in the ductal nuclear clusters highlighted “selenocysteine synthesis” and “SRP-dependent cotranslational protein targeting to the membrane signaling” as candidate pathways mediating the induction of endocrine genes in response to high insulin demand. To validate this possibility, we used the human ductal cell line (PANC-1), a widely used model that has been reported to exhibit plasticity (27, 28). We used the cell line to test its ability to respond to 14 days of stimulation with pathophysiological concentrations of insulin that was observed in the LIRKO mice to simulate the in vivo milieu of insulin resistance (29, 30) (Figure 6A). As expected, insulin (10 μg/mL) induced cell proliferation after 3 days of stimulation, as shown by the higher transcript levels of proliferating cell nuclear antigen (*PCNA*) compared with PBS-treated cells (Figure 6B). The proliferation subsided at 7 and 14 days, consistent with the observation that growth of ductal cells precedes differentiation (7). The cells treated with the highest dose of insulin (20 μg/mL) also showed a tendency toward an increase in *PCNA* expression after 3 days (Figure 6B) and would likely have induced higher cell proliferation at earlier time points. Of relevance to our hypothesis, the cells treated with insulin exhibited a dose-dependent increase in *NGN3*, *PDX1*, and *NKX2.2* expression after 14 days that reached significance for the highest dose (20 μg/mL) compared with PBS-treated samples (Figure 6, C–E).

To evaluate the significance of the interaction between the ‘selenocysteine synthesis’ and the ‘SRP-dependent cotranslational protein targeting to the membrane pathways’ in regulating endocrine cell differentiation in response to high insulin demand, we simultaneously blocked the 2 pathways in PANC-1 cells. We silenced the selenocysteine lyase (*SCLY*) gene, a key enzyme in the selenoprotein metabolism pathway (31), and also blocked the SRP pathway using eeyarestatin 1 (ESI), a pharmacological inhibitor of protein translocation into the ER mediated by the SRP receptor and SEC61α (32). We reasoned that simultaneously targeting the 2 pathways would prevent induction of endocrine gene expression that would otherwise occur if one of the pathways was still active. Scramble and siSCLY+ESI cells were then treated with or without insulin (20 μg/mL for 14 days) (Figure 6F). We confirmed that *SCLY* was silenced over the treatment period by assessing its expression levels in scramble versus siSCLY PANC-1 cells at intermediate time points (Figure 6G). Successful inhibition of the SRP pathway was evident by lower expression of SRP receptor α (*SRPRA*) in siSCLY+ESI cells compared with scramble cells (Figure 6H). Consistent with the sequencing data, insulin treatment significantly increased the expression of genes in the SRP pathway, such as SEC61 translocon subunit α 2 (*SEC61A2*), and endocrine marker genes, such as *PDX1* and *PAX6*, compared with PBS-treated cells (Figure 6, I–K). The depletion of *SCLY* and concomitant blockade of the SRP pathway prevented these effects. These data using the PANC-1 model point to the importance of the “selenocysteine synthesis” and the “SRP-dependent co-translational protein targeting to the membrane” pathways in mediating the insulin-dependent activation of the endocrine lineage in ductal cells. Further confirmation is necessary in primary ductal cells in vivo.

## Discussion

In the present study, we undertook snRNA-Seq in grafts of human islets and ducts to identify β cell sources that are triggered by alterations in ductal epithelium (neogenesis) in response to physiologic (pregnancy model) or pathophysiologic (genetically engineered insulin-resistant models) conditions. The snRNA-Seq approach provides a less biased cellular coverage, provides fewer transcriptional artifacts due to isolation protocols, and is suitable for archived frozen specimens compared with scRNA-Seq procedures (11, 33, 34). UMAP analyses of the grafts containing the transplanted human ductal aggregates revealed multiple ductal clusters consistent with the previously reported scRNA-Seq data on sorted ductal cells or exocrine components obtained from human pancreas in physiological and pathophysiological conditions (24, 35). Raw data were demultiplexed, aligned to the human genome, and collapsed according to the unique molecular identifier (UMI) and by aligning the sequence reads to the murine genome, cells containing >25% mouse-specific UMI were excluded. The detection of a subcluster of nuclei expressing *INS*, *CHGA*, *MAFA*, and *PAX6* within ductal cluster 2 in the graft samples of the pregnancy and insulin-resistant models suggests that a fraction of ductal cells is emerging to express both mature and immature β cell markers, potentially in response to the physiological or pathophysiological insulin demand. These findings are congruent with earlier studies reporting plasticity of human ductal cells in generating β-like cells in states of insulin resistance, such as pregnancy, T2D, or obesity (7, 36, 37). The presence of enriched *INS*/*GCG* double-positive cells in a subpopulation of ductal cells in the experimental models that is not detected in the grafts in control nonpregnant NSG-Lox mice suggests that the differentiation of ductal cells to β-like cells occurs via an intermediate α-like cell during the adaptive response to overt insulin resistance and is consistent with ducts and islets sharing developmental origins (38). An α-like intermediate stage has been reported in mouse models treated with GABA (39) and in pancreas obtained from insulin-resistant humans (40), signifying translational relevance.

An increase in the endocrine cell phenotype is complemented by a fading ductal cell identity reflected by downregulation of adhesion proteins, such as *MUC1*, and ductal cell–specific genes and transcription factors, such as *KRT19* and *ONECUT2*, respectively, in ductal nuclear clusters in the insulin-resistant mice. The reactivation of the Notch pathway is involved in phenotype modulation of rat pancreatic exocrine cells (22), and terminal ductal cells have been reported to harbor activated Notch signaling in adult mice (41). Consistently, we observed that the Notch pathway was downregulated in ductal cluster 2 in the combined model and correlated with a loss of ductal cell identity. The downregulation of ECM/integrin-related pathways in both ductal clusters, especially in the combined model, is consistent with dysregulation of focal adhesion machinery during transdifferentiation of pancreatic progenitors to endocrine cells (23, 42).

Analyses of the pregnancy and insulin-resistant models showed common alterations in the top pathways. For example, an upregulation of genes, such as *ATP1A1*, suggested transdifferentiation toward a β-like cell phenotype. Next, harmonizing the snRNA-Seq data sets to compare the 2 ductal nuclear clusters in all 3 experimental models with endocrine progenitor cells identified in previous scRNA-Seq analysis on human ductal cells (24) revealed clusters of nuclei that resembled the transcriptomic signature of the endocrine progenitor cell clusters of the previously annotated “reference” data set. Among the common genes, at least one-third of those that were differentially regulated were shared between the endocrine progenitor cells and the ductal clusters in insulin-resistant models and included non–β cell genes (e.g., *GCG* and *PPY*), and β cell–specific genes (e.g., *FXYD2* and *FTH1*). Moreover, the combined model was distinct from the individual models of increased insulin demand, and it showed upregulation in the spironolactone action pathway. Spironolactone is a nonselective mineralocorticoid receptor (MR) antagonist known to improve glucose and lipid metabolism (43). Studies on extracts from the tail of the pancreas showed that spironolactone inhibits phosphorylation of protein kinase B and p38MAPK pathways, which are important for cellular apoptosis (44), suggesting protection during the differentiation of ductal epithelium into endocrine cells. Taken together, these data indicate that ductal cells exposed to physiological or pathophysiological insulin resistance begin to express genes that overlap with the transcriptomic profile of endocrine progenitor cells, indicating initiation of transdifferentiation toward the endocrine lineage.

The emergence of “selenocysteine synthesis” as the top upregulated pathway in the ductal clusters is teleologically relevant since selenium, an antiinflammatory and antioxidant molecule (45), would act to protect vulnerable cells from stress especially during high demand for insulin. These effects are especially relevant in pregnancy since reactive oxygen species (ROS) appears in pancreatic cells at E14–E18.5 when neurogenin 3 (Ngn3) expression rises in the endocrine progenitors (46–49). In addition, previous studies have linked this pathway to glucose homeostasis and insulin production in rodents and humans. In particular, (a) SNPs found in the *SCLY* genetic locus were associated with insulin resistance in individuals of Mexican-American descent (50); (b) the whole body *SCLY*-KO mouse model manifested impaired glucose tolerance and metabolic syndrome (51), and such a phenotype was worsened upon challenge with high-fat diet (52); and (c) selenium increased insulin expression and secretion in mouse β cell lines (MIN6 cells) and rat pancreatic islets (53).

A second pathway that was upregulated in all models of insulin resistance was associated with genes in the “SRP-dependent cotranslational protein targeting to the membrane” pathway, which participates in the insulin biosynthesis process (54) and potentially represents the formation of secretory insulin vesicles in emerging β cells. It is notable that the “selenocysteine synthesis” and the “SRP-dependent co-translational protein targeting to the membrane” pathways were also upregulated in β cells in islets from patients with T2D (Figure 5A). However, considering neither the insulin resistance score nor duration of diabetes of the T2D donors were reported, we are unable to directly infer whether the transcriptomic signature of the β cells reflects a compensating versus a failing β cell profile or a mix of both in response to insulin resistance. Notwithstanding, these pathways warrant further investigation to gain insights into ductal progenitors that are critical for β cell adaptation in individuals susceptible to develop diabetes. Taken together with our data from PANC-1 cells, these data suggest that the selenocysteine metabolism pathways plays a fundamental role in coordinating the generation of insulin-producing cells from ductal progenitors to compensate for the high insulin demand. However, the precise mechanisms by which these pathways initiate and regulate the expression of endocrine cell genes in murine versus human ductal cells, especially in the context of adaptive responses in vivo, will require further investigation (55).

**Figure 7:**
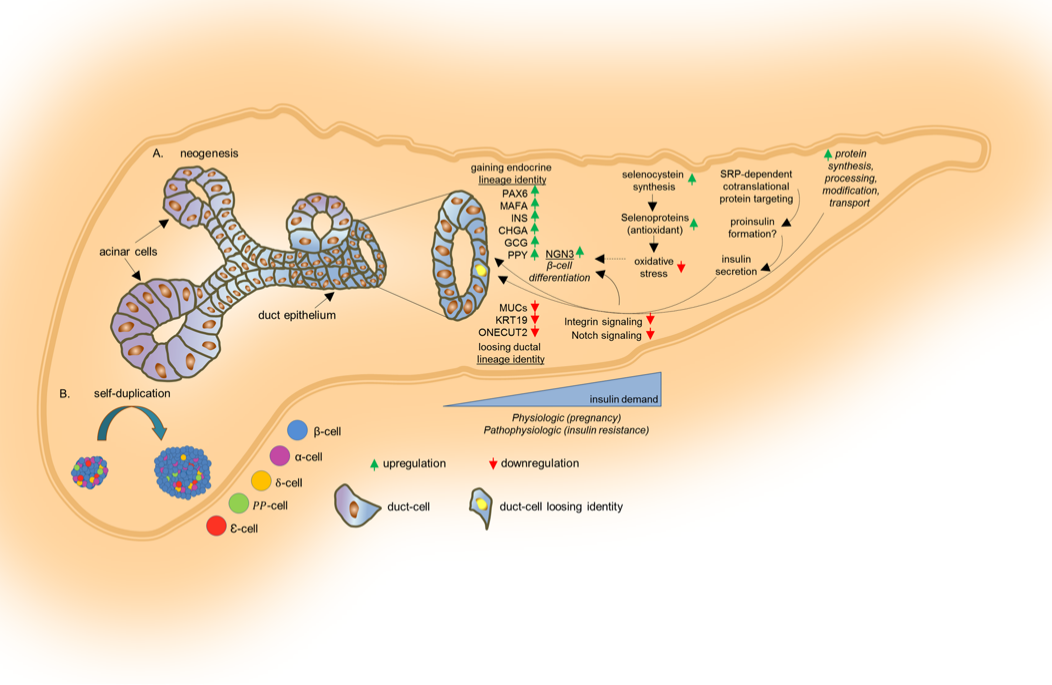
Working model summarizing the transcriptomic modifications in ductal cells in response to increased insulin demand. (**A** and **B**) Physiological (pregnancy) and pathological (insulin-resistant) conditions result in increased β cell mass due to neogenesis (**A**) and/or β cell regeneration (**B**). Ductal epithelial cells contribute in generating new β cells to compensate for the high insulin demand. Increasing levels of insulin resistance lead to the elevated expression of endocrine cell markers, including *PAX6*, *MAFA*, *INS*, and *GCG*; simultaneously, the expression of ductal-specific genes, such as *MUC1*, *ONECUT2*, and *KRT19* is reduced. Such transcriptomic changes are likely due to the increased expression of selenocysteine proteins that potentially regulate the redox state in the differentiating cells, leading to the increased expression of *NGN3*. At the same time, high insulin conditions cause downregulation of integrin signaling and the Notch pathway, ultimately increasing *NGN3* gene expression.

In conclusion, we report a potentially novel transcriptomic approach (Figure 7) using snRNA-Seq to define the signatures of human ductal cells that acquire β cell identity during the adaptive compensatory response to pregnancy and insulin resistance in humanized in vivo models.

## Methods

### Mice.

Mice were housed on a 12-hour light/12-hour dark cycle with water and food ad libitum. *Alb-CreInsR*^fl/fl^ (LIRKO) mice were a gift from C.R. Kahn (Joslin Diabetes Center). NSG mice were purchased from The Jackson Laboratory. Ten- to 12-week-old (7) female immunodeficient NSG-Lox and NSG-LIRKO mice (with or without pregnancy) were used for generating the humanized insulin-resistant mouse models as described previously (7). Male mice were used only as breeders. Pregnancy was confirmed by the presence of a vaginal plug and designated day 0.5 of gestation (G0.5).

### Human islet and duct transplantation studies.

Upon receipt, human islets and human ductal aggregates isolated from nondiabetic donors (*n* = 4) were cultured overnight in Miami Media 1A (Cellgro). Hand-picked and size-matched islets (1000 IEQ) were transplanted together with 100 human ductal aggregates (cluster of ductal cells) under the kidney capsule of both NSG-Lox and NSG-LIRKO mice as described previously (56, 57). After allowing 10 days after transplantation for islet engraftment, mice were either maintained in a nonpregnant state or allowed to breed to become pregnant. Human islet and duct grafts were removed on pregnancy day 15.5 and snap-frozen for further analysis.

### Isolation of nuclei from frozen engrafted samples.

Isolation of nuclei from frozen transplanted specimen was performed as previously reported (11). Briefly, frozen grafts homogenized in Nuclei EZ lysis buffer (NUC-101, MilliporeSigma). Following several steps of washing in 1× DPBS and centrifugations performed at 500*g* for 5 minutes at 4°C, nuclear samples were counted using a cell counter using 0.4% trypan blue stain. The average number of total nuclei obtained from one-half graft was approximately 8.5 × 10^5^ nuclei (1.7 × 10^6^ cells/mL) with 5–10 μm size and 93.3% ± 1.1% dead cell rate (*n* = 31 samples across 3 independent experiments). The number of nuclei was adjusted to 1000 nuclei/μL with suspension buffer, and 10,000 nuclei were immediately used for generation of gel beads in emulsion (GEMs) and barcoding. Leftover nuclei were saved for future analysis.

### snRNA-Seq.

GEMs were generated using the Chromium 3′ Single Cell Library Kit (v2, 10X Genomics) according to the manufacturer’s instructions. Briefly, 10,000 nuclei were combined with Single Cell Master Mix and encapsulated into the barcoded Gel Beads through the Chromium Controller. After GEM–reverse transcription incubation, cDNA samples were recovered, purified, and amplified through a cDNA Amplification Reaction. Quality controls on amplified cDNA samples were carried out through using a High Sensitivity DNA Kit (Agilent) on a 2100 BioAnalizer (Agilent) platform. Libraries were then constructed following fragmentation and adaptor ligation and sample index incorporation. Finally, purified libraries were run on 2100 BioAnalizer (Agilent) using a High Sensitivity DNA Kit (Agilent) to evaluate the quality of the ~400 bp fragments. The final single-nucleus libraries were sequenced using a coverage of 500,000 pair-ended reads targeted per nucleus, on a HiSeq platform (Illumina).

### Analyses of snRNA-Seq data.

The raw snRNA-Seq data were analyzed using previously published analytic pipelines (11). Briefly, raw data were initially demultiplexed, aligned to the human-mouse combined reference genome, and collapsed according to the unique motif identifiers (UMI) by using CellRanger (v2.2.0). Quality controls were computed using the R package Scater including library sizes, number of expressed genes, and proportion of UMIs assigned to mitochondrial genes. With this approach, we removed low-quality nuclei with a small library size, nonbarcoded reads, and cells with a proportion of mitochondrial genes > 20%. Low-abundant genes with average counts < 0.01 were also excluded. CellBender was used to remove ambient RNA contamination (58), and DoubletFinder (12) was applied to remove doublets and multiplets (12), after normalizating the data using sctransform R package (59). Finally, by aligning the sequenced reads to the murine genome (GRCm38), we excluded cells containing >25% mouse-specific UMI. By using Seurat, we generated UMAP plots, allowing identification for clusters and marker genes per cluster. Cell types were classified according to the expression of the pancreatic cell marker genes, as previously described. To discover the differential expressed genes in the 3 experimental models — pregnant NSG-Lox versus nonpregnant NSG-Lox (pregnancy model), nonpregnant NSG-LIRKO versus nonpregnant NSG-Lox (insulin-resistant model), and pregnant NSG-LIRKO versus nonpregnant NSG-Lox (combined model) — we used edgeR package following empirical Bayes quasi likelihood F-tests for comparisons in the several cell types (60). Genes reporting *P* < 0.05 in the pregnancy, insulin-resistant and combined models were considered significantly upregulated or downregulated, respectively. Pathway analysis was performed considering the most upregulated/downregulated genes in all the investigated models within the ductal clusters, using ConsensusPathDB (61).

The snRNA-Seq data included in this study have been deposited in NCBI Gene Expression Omnibus (GEO; accession no. GSE207393; https://www.ncbi.nlm.nih.gov/geo/).

### Reanalysis of published scRNA-Seq data sets.

The public available scRNA-Seq GSE131886 (24) was reanalyzed using the harmonization pipelines (11, 62). With this approach, we generated a reference data set that was projected onto our snRNA-Seq data set to identify common transcriptomic signatures. Focusing on the cluster of cells previously identified as endocrine-progenitor cells in GSE131886 (i.e., *SPP1*^+^ and *TFF1*^+^ cells), we generated lists of cell-specific genes using the R-package limma (63). Such differentially regulated genes (*P* < 0.05) were intersected with the genes differentially regulated in ductal cluster 2 or 4 in pregnant NSG-Lox versus nonpregnant NSG-Lox (pregnancy model), nonpregnant NSG-LIRKO versus nonpregnant NSG-Lox (insulin-resistant model), or pregnant NSG-LIRKO versus nonpregnant NSG-Lox (combined model).

The publicly available scRNA-Seq data set GSE81608 (26) was reanalyzed to reveal the differentially regulated genes in T2D versus control β cells, using the edgeR package (60). The significantly differentially regulated genes (*P* < 0.05) were used in the ConsensusPathDB resource to perform pathway analysis.

To determine the correlations of differentially regulated genes in the Seleno Aminoacid Metabolism (KEGG) and the SRP-dependent cotranslational protein pathways between T2D versus control β cells and the ductal clusters in all the models, we performed linear regression analyses by transforming the *P* values into signed *Z* scores using normal quantile function (qnorm). Linear regression was performed using natural log functions of the *Z* scores.

### Immunostaining and microscopy.

Paraffin-embedded human duct/islet graft sections were processed for IHC as previously described (7). Briefly, we used specific antibodies to target CK19 (Abcam, ab7754, 1:200), insulin (Abcam, ab7842, 1:500), and glucagon (MilliporeSigma, G2654, 1:10,000). Secondary antibodies against the respective host species and conjugated with AlexaFluor 350, 488, or 594 (The Jackson Laboratories) were used to reveal polyhormonal cells. Images were acquired by confocal microscopy using the Zeiss LSM 980 with Airyscan 2 (Zeiss) or Zeiss Axio Imager M1 at 20× magnification. Orthographic projections were generated by using the Zeiss Zen Black Software.

### Cell culture.

PANC-1 cells (ATCC) at passage number 10 were maintained in 1× DMEM (Corning) supplemented with glucose at 4.5 g/L, 10% FBS (Thermo Fisher Scientific), and 1% penicillin/streptomycin (Corning). Insulin treatment was performed by adding 1× PBS or human insulin (MilliporeSigma) at the indicated concentrations every day in the growth medium for 3, 7, or 14 days. To silence the SCLY gene, we used either the ON-TARGETplus Non-targeting (D-001810-10-20, scramble) or SCLY-specific (L-017175-01-0010, siSCLY) small interference RNA pools (Horizon Discovery) at 10 nM. Knock-down experiments were performed every 4 days using lipofectamine RNAiMax (Thermo Fisher Scientific), as previously reported (64). To block the SRP pathway, we treated cells daily with either DMSO (0.1%) or ESI (Tocris) at 1 μg/mL for 14 days. At the end of the experiment, medium was removed and cells were collected for RNA isolation.

### RNA isolation and real-time PCR.

Cells were lysed in TRIzol (Thermo Fisher Scientific), and total RNA was extracted following incubation with chloroform (MilliporeSigma) according to the manufacturer’s instructions. Aqueous phases were purified following incubation with 70% ethanol at 1:1 ratio. The mixtures were then run through RNeasy mini kit columns (Qiagen) to concentrate and isolate high-quality RNA. Following quantification using Nanodrop One spectrophotomer (Thermo Fisher Scientific), cDNA was produced using high-capacity cDNA synthesis kit (Applied Biosystems) according to manufacturer’s instructions. cDNA was amplified using specific oligonucleotides (Supplemental Table 9) using the ABI 7900 system (Applied Biosystems), and gene expression was analyzed using the ΔΔCT method, following normalization on TATA-box binding protein (*TBP*) transcript levels.

### Statistics.

All data are expressed as ± SEM. Statistical significance was determined by 2-way ANOVA test following Bonferroni’s multiple-comparison test and 1-way ANOVA test following Bonferroni’s multiple-comparison test (Graph Pad Prism 7). *P* value of less than 0.05 was considered a significant difference. To correlate the expression levels of genes in the candidate pathways, linear regression analysis on the normalized *Z* scores of gene expression levels was performed, and the significance was tested by F-test. For the pathway analyses performed via ConsensusPathDB, the *P* value was calculated according to the hypergeometric test based on the number of physical entities present in both the predefined set and user-specified list of physical entities (61).

### Study approval.

All mouse experiments were conducted at Joslin Diabetes Center with approval of its IACUC and were in accordance with NIH guidelines. Human islets and ductal aggregates were obtained from the Prodo Laboratories (Supplemental Table 1). All studies and protocols used were approved by the Joslin Diabetes Center’s Committee on Human Studies (CHS#5-05).

## Author contributions

ED conceived the idea, designed and performed the experiments, analyzed the data, and wrote the manuscript. GB researched data and wrote the manuscript. SK researched data and provided technical support and/or critical discussions of the manuscript. DD generated the confocal and fluorescence microscopy data. JH performed the immunostaining experiments. RNK conceived the idea, designed the experiments, directed the project, and wrote the manuscript. All the authors have reviewed the manuscript.

## Supplementary Material

Supplemental data

## Version 1 07/12/2022

In-Press Preview

## Version 2 08/22/2022

Electronic publication
