# Supplementary material for: Single-nucleus RNA-Seq reveals singular gene signatures of human ductal cells during adaptation to insulin resistance
Source: JCI Insight. 2022 Aug 22;7(16):e153877. doi: 10.1172/jci.insight.153877 (PMC9462484; doi:10.1172/jci.insight.153877)
Supplement: Supplemental data [file jciinsight-7-153877-s008.pdf]

Single-nucleus RNA-sequencing reveals singular gene signatures of human ductal cells during adaptation to insulin resistance

Ercument Dirice[1,2,3,*], Giorgio Basile[1,2,*], Sevim Kahraman[1,2], Danielle Diegisser[3], Jiang Hu[1,2]

Rohit N. Kulkarni[1,2,4]

[1] Islet Cell and Regenerative Biology, Joslin Diabetes Center, Boston, Massachusetts, USA.

[2]Department of Medicine, Beth Israel Deaconess Medical Center, Harvard Medical School, Boston, Massachusetts, USA.

[3]Department of Pharmacology, New York Medical College, Valhalla, New York, USA.

[4]Harvard Stem Cell Institute, Boston Massachusetts, USA.

*, these authors contributed equally.

The authors have declared that no conflict of interest exists.

Correspondence to:

Rohit N. Kulkarni M.D. Ph.D,

Islet Cell and Regenerative Biology, Joslin Diabetes Center

One Joslin Place, Boston, 02215 MA

Tel: +1-617-309-3460 – Fax: +1-617-309-3476

Email: Rohit.Kulkarni@joslin.harvard.edu

Supplemental Material

Supplemental Figures

Supplemental Figure 1

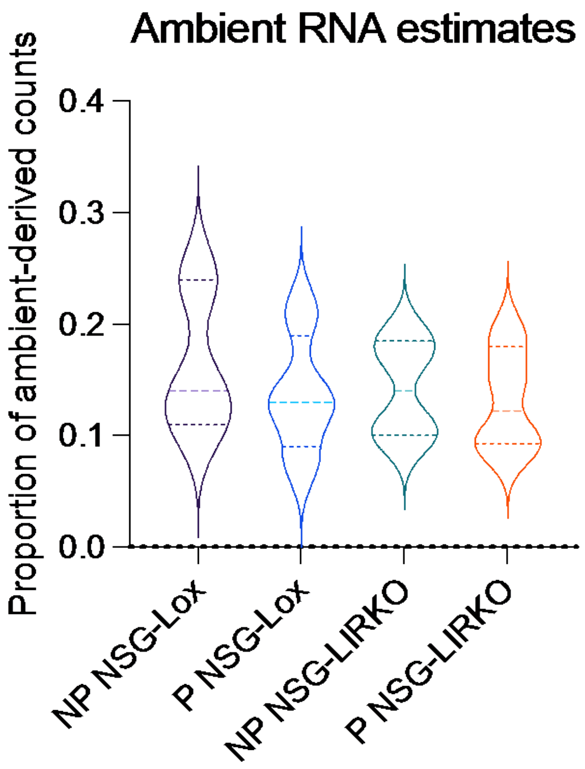

Supplemental Figure 1: Estimation of ambient RNA levels in human grafts. Violin plot of the proportions of ambient RNA contamination in droplets from human islets and ductal cells transplanted in non-pregnant NSG-Lox (NP NSG-Lox, purple), pregnant NSG-Lox (P NSG-Lox, blue), non-pregnant NSG-LIRKO (NP NSG-LIRKO), and pregnant NSG-LIRKO (P NSG-LIRKO). N=4/group.

Supplemental Figure 2

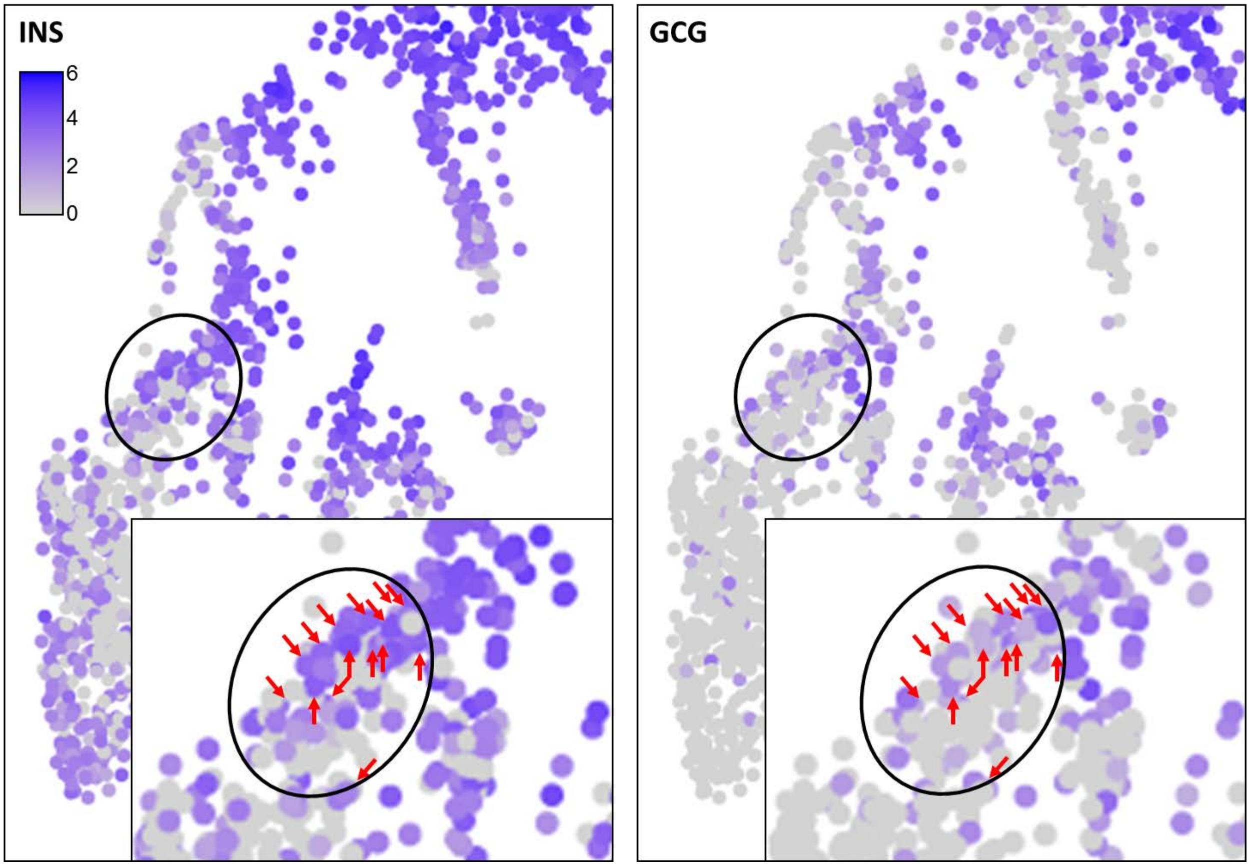

Supplemental Figure 2: A specific ductal sub-population is enriched in insulin/glucagon double positive nuclei. Image shows magnified area from Figure 2B (solid circle within the dotted circle). Further magnified area shows red arrows pointing cells that express both insulin and glucagon within the ductal clusters in given UMAPs.

Supplemental Figure 3

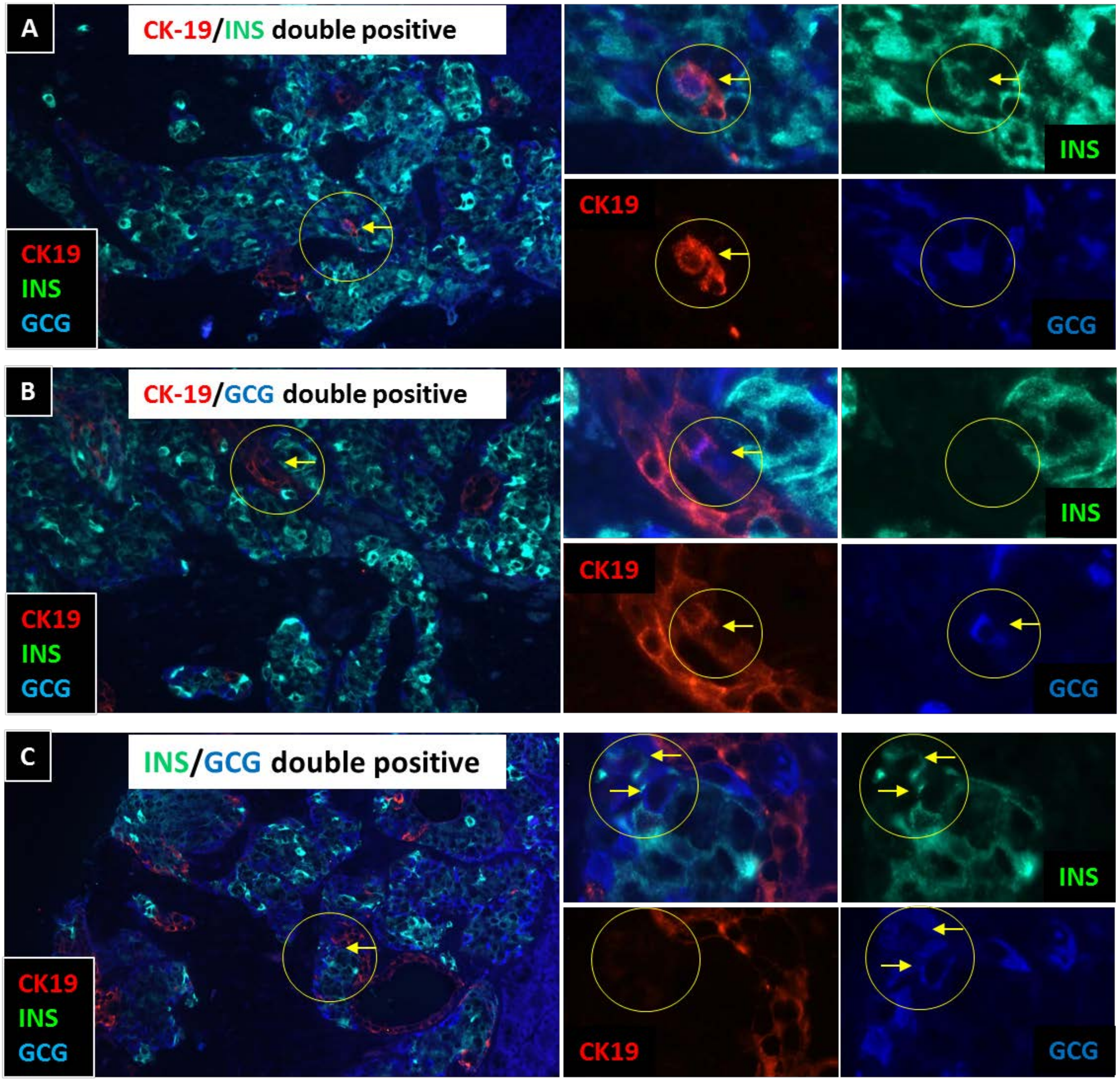

Supplemental Figure 3: Mature β- and α-cell markers are co-expressed in human ductal cells. (A-C) Representative fluorescence images of human islet and duct graft samples immunostained for CK19 (red), insulin (green), and, glucagon (blue) showing (A) CK19/INS, (B) CK19/GCG, and (C) INS/CGC double expressing cells. Yellow circles and arrows show cells expressing both markers.

Supplemental Figure 4

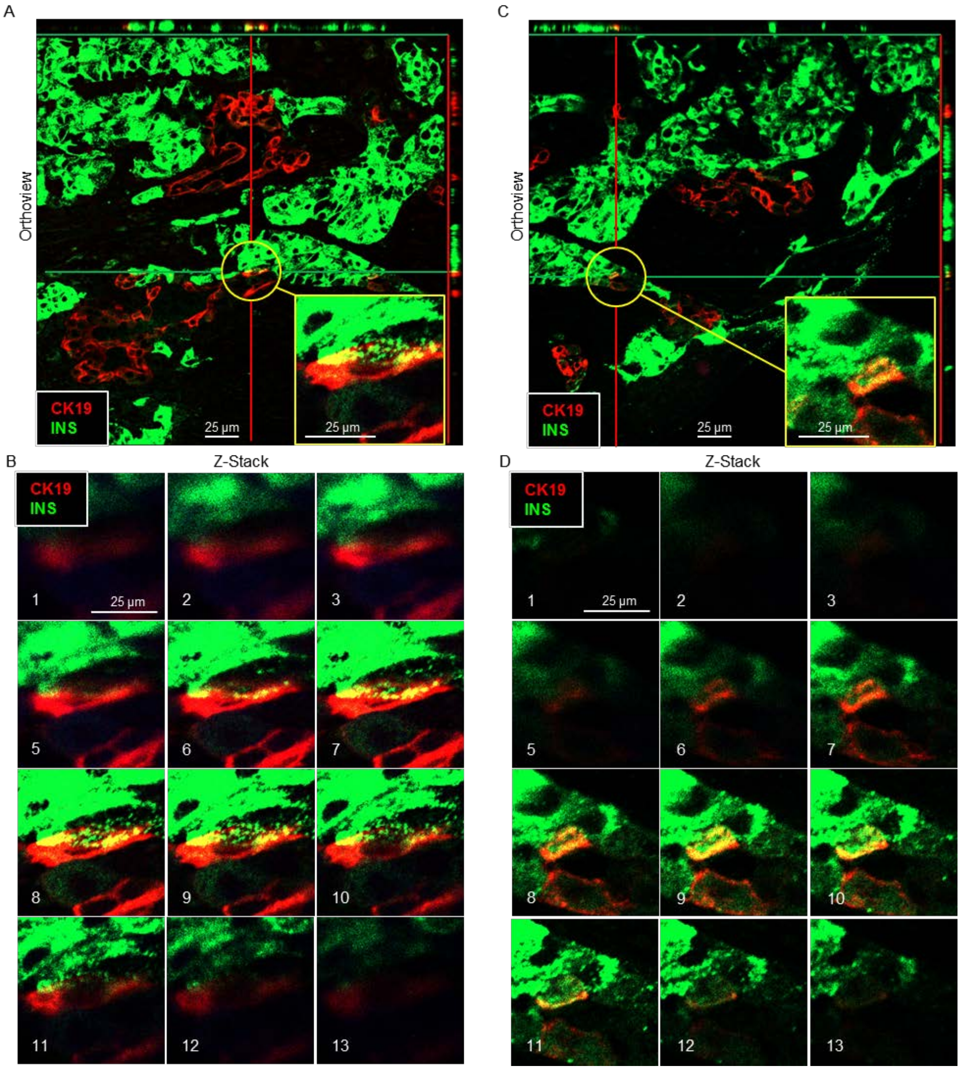

Supplemental Figure 4: CK19 and insulin is co-expressed in human duct and islet grafts samples. (A and C) Representative confocal images showing orthographic views of human islet and duct graft samples immunostained for CK19 (red) and insulin (green). Co-expression of CK19 and insulin is shown in yellow within the yellow circle. Inset in the bottom right shows the magnified image of CK19 and insulin co-expressing cells. (B and D) Images 1-13 are single slices taken from the z-stack in *A* and *C* showing different focal planes co-expressing CK19 and insulin. Scale bar: 25 µm.

Supplemental Figure 5

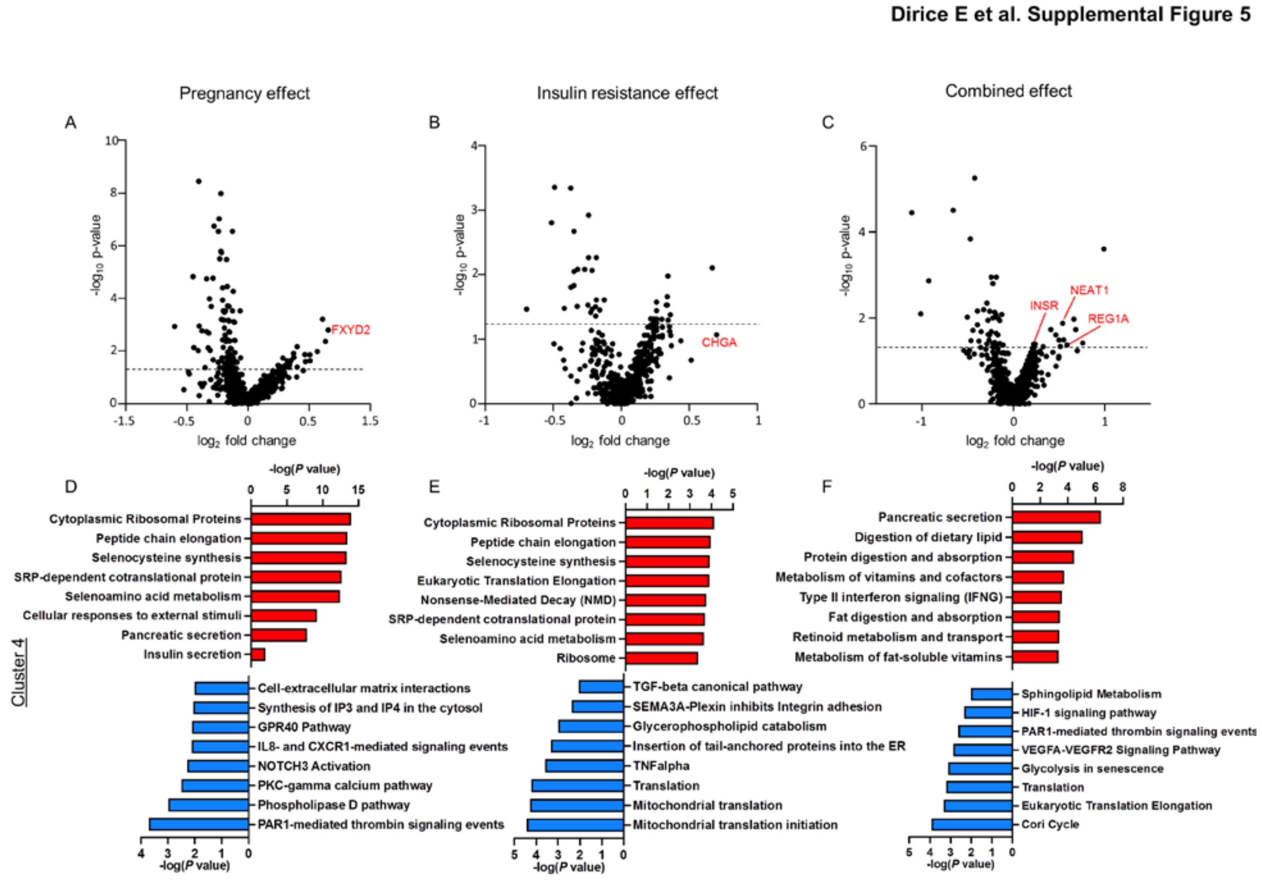

Supplemental Figure 5: Differentially regulated genes and pathways in ductal cluster #4 in pregnancy, insulin resistant or combined models. (**A-C**) Volcano plots showing the distribution of differential expression of transcripts defined as a function of fold change within the (**A**) pregnancy (P NSG-Lox vs. NP NSG-Lox), (**B**) insulin resistant (NP NSG-LIRKO vs. NP NSG-Lox), or (**C**) combined (P NSG-LIRKO vs. NP NSG-Lox) models and *P* value for ductal cluster #4. (**D-F**) Selected pathways in ductal cluster #4 differentially regulated in (**D**) pregnancy (P NSG-Lox vs. NP NSG-Lox), (**E**) insulin resistant (NP NSG-LIRKO vs. NP NSG-Lox), or (**F**) combined (P NSG-LIRKO vs. NP NSG-Lox) models. Upregulated pathways are shown in red, downregulated pathways are shown in blue.

Supplemental Tables

Supplemental Table 1. Clinical characteristics of the human islet and duct donors.

M; Male, F; Female, AA; W; White, BMI; Body Mass Index.

Supplemental Table 2: Selected differentially regulated pathways in the ductal cluster #2 of pregnant NSG-Lox mice compared to non-pregnant NSG-Lox mice (Pregnancy model). List of selected pathways analyzed by ConsensusPathDB. The p-value is calculated according to the hypergeometric test based on the number of physical entities present in both the predefined set and user-specified list of physical entities. Reference is cited in Methods.

Supplemental Table 3. Selected differentially regulated pathways in the ductal cluster #2 of non-pregnant NSG-LIRKO mice compared to non-pregnant NSG-Lox mice (Insulin Resistant model). List of selected pathways analyzed by ConsensusPathDB. The P value is calculated according to the hypergeometric test based on the number of physical entities present in both the predefined set and user-specified list of physical entities. Reference is cited in Methods.

Supplemental Table 4. Selected differentially regulated pathways in the ductal cluster #2 of pregnant NSG-LIRKO mice compared to non-pregnant NSG-Lox mice (Combined model). List of selected pathways analyzed by ConsensusPathDB. The P value is calculated according to the hypergeometric test based on the number of physical entities present in both the predefined set and user-specified list of physical entities. Reference is cited in Methods.

Supplemental Table 5. Selected differentially regulated pathways in the ductal cluster #4 of pregnant NSG-Lox mice compared to non-pregnant NSG-Lox mice (Pregnancy model). List of selected pathways analyzed by ConsensusPathDB. The P value is calculated according to the hypergeometric test based on the number of physical entities present in both the predefined set and user-specified list of physical entities. Reference is cited in Methods.

Supplemental Table 6: Selected differentially regulated pathways in the ductal cluster #4 of non-pregnant NSG-LIRKO mice compared to non-pregnant NSG-Lox mice (Insulin Resistant model). List of selected pathways analyzed by ConsensusPathDB. The P value is calculated according to the hypergeometric test based on the number of physical entities present in both the predefined set and user-specified list of physical entities. Reference is cited in Methods.

Supplemental Table 7. Selected differentially regulated pathways in the ductal cluster #4 of pregnant NSG-LIRKO mice compared to non-pregnant NSG-Lox mice (Combined model). List of selected pathways analyzed by ConsensusPathDB. The P value is calculated according to the hypergeometric test based on the number of physical entities present in both the predefined set and user-specified list of physical entities. Reference is cited in Methods.

Supplemental Table 8. Selected differentially regulated pathways in type 2 diabetic human beta cells compared to non-diabetic cases (Publicly Available dataset, GSE81608). The P value is calculated according to the hypergeometric test based on the number of physical entities

present in both the predefined set and user-specified list of physical entities. Reference is cited in Methods.

Supplemental Table 9. Sequences of oligonucleotides used for real time PCR.

Supplemental Table 1. Clinical characteristics of the human islet and duct donors.

**Human Islet + Duct Transplantation** (ducts were isolated from same donor)							
**Figure**	**Source and Case ID**	**Gender**	**Age**	**Ethnicity**	**BMI**	**Purity (%)**	**Viability (%)**
3-Jan	Prodo, HP17272	M	48	W	23.9	90	95
	Prodo, HP17281	M	55	W	30.1	90	95
	Prodo, HP-17307	M	55	W	27.1	90	95
	Prodo, HP-17328	F	48	W	25	90	95

M; Male, F; Female, AA; W; White, BMI; Body Mass Index.

Supplemental Table 2. Selected differentially regulated pathways in the ductal cluster #2 of pregnant NSG-Lox mice compared to non-pregnant NSG-Lox mice (Pregnancy model)

List of selected pathways analyzed by ConsensusPathDB.
The p-value is calculated according to the hypergeometric test based on the number of physical entities present in both the predefined set and user-specified list of physical entities. Reference is cited in Methods.

UPREGULATED pathways	set size	candidates contained	p value	q value	Pathways database
SRP-dependent cotranslational protein targeting to membrane	123	8 (6.5%)	2.55E-11	6.46E-09	Reactome
Cytoplasmic Ribosomal Proteins	88	7 (8.0%)	1.28E-10	1.62E-08	Wikipathways
Peptide chain elongation	100	7 (7.0%)	3.19E-10	1.63E-08	Reactome
Eukaryotic Translation Termination	103	7 (6.8%)	3.94E-10	1.63E-08	Reactome
Selenocysteine synthesis	103	7 (6.8%)	3.94E-10	1.63E-08	Reactome
Nonsense Mediated Decay (NMD) independent of the Exon Junction Complex (EJC)	105	7 (6.7%)	4.52E-10	1.63E-08	Reactome
Eukaryotic Translation Elongation	105	7 (6.7%)	4.52E-10	1.63E-08	Reactome
Response of EIF2AK4 (GCN2) to amino acid deficiency	111	7 (6.3%)	6.69E-10	2.12E-08	Reactome
Formation of a pool of free 40S subunits	113	7 (6.2%)	7.60E-10	2.14E-08	Reactome
Nonsense Mediated Decay (NMD) enhanced by the Exon Junction Complex (EJC)	117	7 (6.0%)	9.71E-10	2.23E-08	Reactome
Nonsense-Mediated Decay (NMD)	117	7 (6.0%)	9.71E-10	2.23E-08	Reactome
L13a-mediated translational silencing of Ceruloplasmin expression	123	7 (5.7%)	1.38E-09	2.85E-08	Reactome
GTP hydrolysis and joining of the 60S ribosomal subunit	124	7 (5.6%)	1.46E-09	2.85E-08	Reactome
Selenoamino acid metabolism	128	7 (5.5%)	1.83E-09	3.30E-08	Reactome
Cap-dependent Translation Initiation	131	7 (5.3%)	2.15E-09	3.40E-08	Reactome
Eukaryotic Translation Initiation	131	7 (5.3%)	2.15E-09	3.40E-08	Reactome
Cellular response to starvation	145	7 (4.8%)	4.38E-09	6.52E-08	Reactome
Ribosome - Homo sapiens (human)	158	7 (4.4%)	7.97E-09	1.12E-07	KEGG
Metabolism	1954	16 (0.8%)	8.59E-09	1.14E-07	Reactome
Cellular responses to stress	554	10 (1.8%)	1.50E-08	1.90E-07	Reactome
Cellular responses to external stimuli	569	10 (1.8%)	1.94E-08	2.33E-07	Reactome
Translation	307	8 (2.6%)	3.63E-08	4.18E-07	Reactome
Formation of the ternary complex, and subsequently, the 43S complex	59	5 (8.5%)	5.48E-08	6.03E-07	Reactome
Metabolism of amino acids and derivatives	339	8 (2.4%)	7.83E-08	8.25E-07	Reactome
Ribosomal scanning and start codon recognition	66	5 (7.6%)	9.70E-08	9.44E-07	Reactome
Translation initiation complex formation	66	5 (7.6%)	9.70E-08	9.44E-07	Reactome
Activation of the mRNA upon binding of the cap-binding complex and eIFs, and subsequent bir	67	5 (7.5%)	1.05E-07	9.81E-07	Reactome
Coronavirus disease - COVID-19 - Homo sapiens (human)	232	7 (3.0%)	1.10E-07	9.90E-07	KEGG
Spironolactone Action Pathway	33	3 (9.1%)	2.64E-05	0.000117	SMPDB
Eplerenone Action Pathway	33	3 (9.1%)	2.64E-05	0.000117	SMPDB
Triamterene Action Pathway	33	3 (9.1%)	2.64E-05	0.000117	SMPDB
Amiloride Action Pathway	33	3 (9.1%)	2.64E-05	0.000117	SMPDB
Torsemide Action Pathway	33	3 (9.1%)	2.64E-05	0.000117	SMPDB
Iminoglycinuria	33	3 (9.1%)	2.64E-05	0.000117	SMPDB
Lysinuric Protein Intolerance	33	3 (9.1%)	2.64E-05	0.000117	SMPDB
Blue diaper syndrome	33	3 (9.1%)	2.64E-05	0.000117	SMPDB
Lysinuric protein intolerance (LPI)	33	3 (9.1%)	2.64E-05	0.000117	SMPDB
Cystinuria	33	3 (9.1%)	2.64E-05	0.000117	SMPDB
Hartnup Disorder	33	3 (9.1%)	2.64E-05	0.000117	SMPDB
Glucose Transporter Defect (SGLT2)	33	3 (9.1%)	2.64E-05	0.000117	SMPDB
Kidney Function	33	3 (9.1%)	2.64E-05	0.000117	SMPDB
Glucose Transporter Defect (SGLT2)	33	3 (9.1%)	2.64E-05	0.000117	SMPDB
Quinethazone Action Pathway	33	3 (9.1%)	2.64E-05	0.000117	SMPDB
Bendroflumethiazide Action Pathway	33	3 (9.1%)	2.64E-05	0.000117	SMPDB
Chlorthalidone Action Pathway	33	3 (9.1%)	2.64E-05	0.000117	SMPDB
Trichlormethiazide Action Pathway	33	3 (9.1%)	2.64E-05	0.000117	SMPDB
Indapamide Action Pathway	33	3 (9.1%)	2.64E-05	0.000117	SMPDB
Metolazone Action Pathway	33	3 (9.1%)	2.64E-05	0.000117	SMPDB
Hydrochlorothiazide Action Pathway	33	3 (9.1%)	2.64E-05	0.000117	SMPDB
Cyclothiazide Action Pathway	33	3 (9.1%)	2.64E-05	0.000117	SMPDB
Hydroflumethiazide Action Pathway	33	3 (9.1%)	2.64E-05	0.000117	SMPDB
Bumetanide Action Pathway	33	3 (9.1%)	2.64E-05	0.000117	SMPDB
Ethacrynic Acid Action Pathway	33	3 (9.1%)	2.64E-05	0.000117	SMPDB
Furosemide Action Pathway	33	3 (9.1%)	2.64E-05	0.000117	SMPDB
Polythiazide Action Pathway	33	3 (9.1%)	2.64E-05	0.000117	SMPDB
Methyclothiazide Action Pathway	33	3 (9.1%)	2.64E-05	0.000117	SMPDB
Chlorothiazide Action Pathway	33	3 (9.1%)	2.64E-05	0.000117	SMPDB
Metabolism of RNA	584	7 (1.2%)	5.07E-05	0.000221	Reactome
Proximal tubule transport	57	3 (5.3%)	0.000138	0.00059	Wikipathways
Lactose Degradation	11	2 (18.2%)	0.000167	0.000691	SMPDB
Lactose Intolerance	11	2 (18.2%)	0.000167	0.000691	SMPDB
Trehalose Degradation	12	2 (16.7%)	0.0002	0.000815	SMPDB
Thyroid hormone synthesis - Homo sapiens (human)	75	3 (4.0%)	0.000311	0.00125	KEGG
Potential therapeutics for SARS	80	3 (3.8%)	0.000376	0.00149	Reactome
Formation of ATP by chemiosmotic coupling	18	2 (11.1%)	0.00046	0.00176	Reactome
Cristae formation	18	2 (11.1%)	0.00046	0.00176	Reactome
Insulin secretion - Homo sapiens (human)	86	3 (3.5%)	0.000465	0.00176	KEGG
Cardiac muscle contraction - Homo sapiens (human)	87	3 (3.4%)	0.000481	0.00179	KEGG
Purine metabolism	222	4 (1.8%)	0.000595	0.00218	EHMN
Chaperone Mediated Autophagy	22	2 (9.1%)	0.000691	0.00248	Reactome
Thermogenesis - Homo sapiens (human)	232	4 (1.7%)	0.000702	0.00248	KEGG
Proximal tubule bicarbonate reclamation - Homo sapiens (human)	23	2 (8.7%)	0.000756	0.00248	KEGG
Response of Mtb to phagocytosis	23	2 (8.7%)	0.000756	0.00248	Reactome
Pancreatic secretion - Homo sapiens (human)	102	3 (2.9%)	0.000766	0.00248	KEGG
Electron Transport Chain (OXPHOS system in mitochondria)	103	3 (2.9%)	0.000788	0.00248	Wikipathways
Protein digestion and absorption - Homo sapiens (human)	103	3 (2.9%)	0.000788	0.00248	KEGG
HSF1 activation	25	2 (8.0%)	0.000895	0.00248	Reactome
SARS-CoV Infections	111	3 (2.7%)	0.000979	0.00248	Reactome
Respiratory electron transport, ATP synthesis by chemiosmotic coupling, and heat production l	113	3 (2.7%)	0.00103	0.00248	Reactome
Infection with Mycobacterium tuberculosis	27	2 (7.4%)	0.00104	0.00248	Reactome
Metabolism of proteins	1935	10 (0.5%)	0.00106	0.00248	Reactome
Levomethadyl Acetate Action Action Pathway	29	2 (6.9%)	0.00121	0.00248	SMPDB
Levallorphan Action Pathway	29	2 (6.9%)	0.00121	0.00248	SMPDB
Dimethylthiambutene Action Pathway	29	2 (6.9%)	0.00121	0.00248	SMPDB
Ethylmorphine Action Pathway	29	2 (6.9%)	0.00121	0.00248	SMPDB

Pentazocine Action Pathway	29	2 (6.9%)	0.00121	0.00248	**SMPDB**
Naltrexone Action Pathway	29	2 (6.9%)	0.00121	0.00248	**SMPDB**
Buprenorphine Action Pathway	29	2 (6.9%)	0.00121	0.00248	**SMPDB**
Alvimopan Action Pathway	29	2 (6.9%)	0.00121	0.00248	**SMPDB**
Naloxone Action Pathway	29	2 (6.9%)	0.00121	0.00248	**SMPDB**
Dihydromorphine Action Pathway	29	2 (6.9%)	0.00121	0.00248	**SMPDB**
Ketobemidone Action Pathway	29	2 (6.9%)	0.00121	0.00248	**SMPDB**
Levorphanol Action Pathway	29	2 (6.9%)	0.00121	0.00248	**SMPDB**
Propoxyphene Action Pathway	29	2 (6.9%)	0.00121	0.00248	**SMPDB**
Tramadol Action Action Pathway	29	2 (6.9%)	0.00121	0.00248	**SMPDB**
Diphenoxylate Action Pathway	29	2 (6.9%)	0.00121	0.00248	**SMPDB**
Anileridine Action Pathway	29	2 (6.9%)	0.00121	0.00248	**SMPDB**
Oxycodone Action Pathway	29	2 (6.9%)	0.00121	0.00248	**SMPDB**
Alfentanil Action Pathway	29	2 (6.9%)	0.00121	0.00248	**SMPDB**
Oxymorphone Action Pathway	29	2 (6.9%)	0.00121	0.00248	**SMPDB**
Hydrocodone Action Pathway	29	2 (6.9%)	0.00121	0.00248	**SMPDB**
Hydromorphone Action Pathway	29	2 (6.9%)	0.00121	0.00248	**SMPDB**
Sufentanil Action Pathway	29	2 (6.9%)	0.00121	0.00248	**SMPDB**
Remifentanil Action Pathway	29	2 (6.9%)	0.00121	0.00248	**SMPDB**
Fentanyl Action Pathway	29	2 (6.9%)	0.00121	0.00248	**SMPDB**
Carfentanil Action Pathway	29	2 (6.9%)	0.00121	0.00248	**SMPDB**
3-Methylthiofentanyl Action Pathway	29	2 (6.9%)	0.00121	0.00248	**SMPDB**
Methadyl Acetate Action Pathway	29	2 (6.9%)	0.00121	0.00248	**SMPDB**
Dezocine Action Pathway	29	2 (6.9%)	0.00121	0.00248	**SMPDB**
Mepivacaine Action Pathway	29	2 (6.9%)	0.00121	0.00248	**SMPDB**
Chloroprocaine Action Pathway	29	2 (6.9%)	0.00121	0.00248	**SMPDB**
Dibucaine Action Pathway	29	2 (6.9%)	0.00121	0.00248	**SMPDB**
Levobupivacaine Action Pathway	29	2 (6.9%)	0.00121	0.00248	**SMPDB**
Benzocaine Action Pathway	29	2 (6.9%)	0.00121	0.00248	**SMPDB**
Bupivacaine Action Pathway	29	2 (6.9%)	0.00121	0.00248	**SMPDB**
Oxybuprocaine Action Pathway	29	2 (6.9%)	0.00121	0.00248	**SMPDB**
Prilocaine Action Pathway	29	2 (6.9%)	0.00121	0.00248	**SMPDB**
Procaine Action Pathway	29	2 (6.9%)	0.00121	0.00248	**SMPDB**
Proparacaine Action Pathway	29	2 (6.9%)	0.00121	0.00248	**SMPDB**
Ropivacaine Action Pathway	29	2 (6.9%)	0.00121	0.00248	**SMPDB**
Cocaine Action Pathway	29	2 (6.9%)	0.00121	0.00248	**SMPDB**
Escitalopram Action Pathway	29	2 (6.9%)	0.00121	0.00248	**SMPDB**
Attenuation phase	29	2 (6.9%)	0.00121	0.00248	**Reactome**
Nalbuphine Action Pathway	30	2 (6.7%)	0.00129	0.00261	**SMPDB**
Desipramine Action Pathway	30	2 (6.7%)	0.00129	0.00261	**SMPDB**
Lidocaine (Local Anaesthetic) Action Pathway	31	2 (6.5%)	0.00138	0.00277	**SMPDB**
Heroin Action Pathway	32	2 (6.2%)	0.00147	0.0029	**SMPDB**
Codeine Action Pathway	32	2 (6.2%)	0.00147	0.0029	**SMPDB**
Imipramine Action Pathway	33	2 (6.1%)	0.00156	0.00306	**SMPDB**
Oxidative phosphorylation - Homo sapiens (human)	133	3 (2.3%)	0.00165	0.0032	**KEGG**
Fluoxetine Action Pathway	34	2 (5.9%)	0.00166	0.0032	**SMPDB**
Infectious disease	750	6 (0.8%)	0.00168	0.00322	**Reactome**
Nicotine Action Pathway	36	2 (5.6%)	0.00186	0.00348	**SMPDB**
Methadone Action Pathway	36	2 (5.6%)	0.00186	0.00348	**SMPDB**
Citalopram Action Pathway	36	2 (5.6%)	0.00186	0.00348	**SMPDB**
Aldosterone-regulated sodium reabsorption - Homo sapiens (human)	37	2 (5.4%)	0.00196	0.00362	**KEGG**
HSF1-dependent transactivation	37	2 (5.4%)	0.00196	0.00362	**Reactome**
adenosine ribonucleotides *de novo* biosynthesis	38	2 (5.3%)	0.00207	0.00379	**HumanCyc**
Ion homeostasis	44	2 (4.5%)	0.00276	0.005	**Reactome**
Morphine Action Pathway	44	2 (4.5%)	0.00276	0.005	**SMPDB**
Retinoid metabolism and transport	45	2 (4.4%)	0.00289	0.00515	**Reactome**
The citric acid (TCA) cycle and respiratory electron transport	162	3 (1.9%)	0.00289	0.00515	**Reactome**
Ion channel transport	165	3 (1.8%)	0.00305	0.00539	**Reactome**
Carbohydrate digestion and absorption - Homo sapiens (human)	47	2 (4.3%)	0.00315	0.00549	**KEGG**
Ion transport by P-type ATPases	47	2 (4.3%)	0.00315	0.00549	**Reactome**
Metabolism of fat-soluble vitamins	49	2 (4.1%)	0.00342	0.00592	**Reactome**
Endocrine and other factor-regulated calcium reabsorption - Homo sapiens (human)	53	2 (3.8%)	0.00399	0.00674	**KEGG**
Mitochondrial biogenesis	53	2 (3.8%)	0.00399	0.00674	**Reactome**
Mineral absorption - Homo sapiens (human)	59	2 (3.4%)	0.00492	0.00674	**KEGG**
superpathway of purine nucleotide salvage	59	2 (3.4%)	0.00492	0.00674	**HumanCyc**
purine nucleotides *de novo* biosynthesis	59	2 (3.4%)	0.00492	0.00674	**HumanCyc**
Bopindolol Action Pathway	61	2 (3.3%)	0.00525	0.00674	**SMPDB**
Timolol Action Pathway	61	2 (3.3%)	0.00525	0.00674	**SMPDB**
Carteolol Action Pathway	61	2 (3.3%)	0.00525	0.00674	**SMPDB**
Bevantolol Action Pathway	61	2 (3.3%)	0.00525	0.00674	**SMPDB**
Practolol Action Pathway	61	2 (3.3%)	0.00525	0.00674	**SMPDB**
Dobutamine Action Pathway	61	2 (3.3%)	0.00525	0.00674	**SMPDB**
Isoprenaline Action Pathway	61	2 (3.3%)	0.00525	0.00674	**SMPDB**
Arbutamine Action Pathway	61	2 (3.3%)	0.00525	0.00674	**SMPDB**
Levobunolol Action Pathway	61	2 (3.3%)	0.00525	0.00674	**SMPDB**
Metipranolol Action Pathway	61	2 (3.3%)	0.00525	0.00674	**SMPDB**
Sotalol Action Pathway	61	2 (3.3%)	0.00525	0.00674	**SMPDB**
Epinephrine Action Pathway	61	2 (3.3%)	0.00525	0.00674	**SMPDB**
Betaxolol Action Pathway	61	2 (3.3%)	0.00525	0.00674	**SMPDB**
Atenolol Action Pathway	61	2 (3.3%)	0.00525	0.00674	**SMPDB**
Alprenolol Action Pathway	61	2 (3.3%)	0.00525	0.00674	**SMPDB**
Acebutolol Action Pathway	61	2 (3.3%)	0.00525	0.00674	**SMPDB**
Propranolol Action Pathway	61	2 (3.3%)	0.00525	0.00674	**SMPDB**
Pindolol Action Pathway	61	2 (3.3%)	0.00525	0.00674	**SMPDB**
Penbutolol Action Pathway	61	2 (3.3%)	0.00525	0.00674	**SMPDB**
Oxprenolol Action Pathway	61	2 (3.3%)	0.00525	0.00674	**SMPDB**
Metoprolol Action Pathway	61	2 (3.3%)	0.00525	0.00674	**SMPDB**
Esmolol Action Pathway	61	2 (3.3%)	0.00525	0.00674	**SMPDB**
Bisoprolol Action Pathway	61	2 (3.3%)	0.00525	0.00674	**SMPDB**
Bupranolol Action Pathway	61	2 (3.3%)	0.00525	0.00674	**SMPDB**
Nebivolol Action Pathway	61	2 (3.3%)	0.00525	0.00674	**SMPDB**

Nadolol Action Pathway	61	2 (3.3%)	0.00525	0.00674	**SMPDB**
Muscle/Heart Contraction	61	2 (3.3%)	0.00525	0.00674	**SMPDB**
Diltiazem Action Pathway	61	2 (3.3%)	0.00525	0.00674	**SMPDB**
Amlodipine Action Pathway	61	2 (3.3%)	0.00525	0.00674	**SMPDB**
Verapamil Action Pathway	61	2 (3.3%)	0.00525	0.00674	**SMPDB**
Nitrendipine Action Pathway	61	2 (3.3%)	0.00525	0.00674	**SMPDB**
Nisoldipine Action Pathway	61	2 (3.3%)	0.00525	0.00674	**SMPDB**
Nimodipine Action Pathway	61	2 (3.3%)	0.00525	0.00674	**SMPDB**
Isradipine Action Pathway	61	2 (3.3%)	0.00525	0.00674	**SMPDB**
Nifedipine Action Pathway	61	2 (3.3%)	0.00525	0.00674	**SMPDB**
Felodipine Action Pathway	61	2 (3.3%)	0.00525	0.00674	**SMPDB**
Quinidine Action Pathway	61	2 (3.3%)	0.00525	0.00674	**SMPDB**
Procainamide (Antiarrhythmic) Action Pathway	61	2 (3.3%)	0.00525	0.00674	**SMPDB**
Disopyramide Action Pathway	61	2 (3.3%)	0.00525	0.00674	**SMPDB**
Fosphenytoin (Antiarrhythmic) Action Pathway	61	2 (3.3%)	0.00525	0.00674	**SMPDB**
Mexiletine Action Pathway	61	2 (3.3%)	0.00525	0.00674	**SMPDB**
Tocainide Action Pathway	61	2 (3.3%)	0.00525	0.00674	**SMPDB**
Flecainide Action Pathway	61	2 (3.3%)	0.00525	0.00674	**SMPDB**
Amiodarone Action Pathway	61	2 (3.3%)	0.00525	0.00674	**SMPDB**
Ibutilide Action Pathway	61	2 (3.3%)	0.00525	0.00674	**SMPDB**
Oxidative phosphorylation	61	2 (3.3%)	0.00525	0.00674	**Wikipathways**
Carvedilol Action Pathway	62	2 (3.2%)	0.00542	0.00689	**SMPDB**
Labetalol Action Pathway	62	2 (3.2%)	0.00542	0.00689	**SMPDB**
Lidocaine (Antiarrhythmic) Action Pathway	63	2 (3.2%)	0.00559	0.00707	**SMPDB**
Cytoprotection by HMOX1	65	2 (3.1%)	0.00594	0.00748	**Reactome**
cAMP signaling pathway - Homo sapiens (human)	216	3 (1.4%)	0.00647	0.0081	**KEGG**
EGFR1	455	4 (0.9%)	0.00799	0.00996	**NetPath**
Gastric acid secretion - Homo sapiens (human)	76	2 (2.6%)	0.00804	0.00997	**KEGG**
Disease	1029	6 (0.6%)	0.00808	0.00997	**Reactome**
Phenytoin (Antiarrhythmic) Action Pathway	82	2 (2.4%)	0.00931	0.0114	**SMPDB**
Parkinson disease - Homo sapiens (human)	249	3 (1.2%)	0.00955	0.0117	**KEGG**

DOWNGULATED pathways	set size	candidates contained	p value	q value	Pathways database
RHOT2 GTPase cycle	7	3 (42.9%)	0.00217	0.513	**Reactome**
Miro GTPase Cycle	8	3 (37.5%)	0.00336	0.513	**Reactome**
S Phase	104	11 (10.6%)	0.00374	0.513	**Reactome**
Synthesis of DNA	76	9 (11.8%)	0.00398	0.513	**Reactome**
Translesion synthesis by POLK	17	4 (23.5%)	0.00446	0.513	**Reactome**
fatty acid β-oxidation (unsaturated, odd number)	3	2 (66.7%)	0.00497	0.513	**HumanCyc**
DNA Replication	81	9 (11.1%)	0.00608	0.513	**Reactome**
Dual Incision in GG-NER	41	6 (14.6%)	0.00635	0.513	**Reactome**
Global Genome Nucleotide Excision Repair (GG-NER)	70	8 (11.4%)	0.00801	0.513	**Reactome**
Nucleotide Excision Repair	43	6 (14.0%)	0.00804	0.513	**Wikipathways**
Post-translational protein modification	1425	77 (5.4%)	0.00808	0.513	**Reactome**
p53 transcriptional gene network	72	8 (11.1%)	0.00946	0.513	**Wikipathways**
Astrocytic Glutamate-Glutamine Uptake And Metabolism	4	2 (50.0%)	0.00967	0.513	**Reactome**
Neurotransmitter uptake and metabolism In glial cells	4	2 (50.0%)	0.00967	0.513	**Reactome**
Conversion from APC/C:Cdc20 to APC/C:Cdh1 in late anaphase	21	4 (19.0%)	0.00985	0.513	**Reactome**
Phosphorylation of the APC/C	21	4 (19.0%)	0.00985	0.513	**Reactome**

Supplemental Table 3. Selected differentially regulated pathways in the ductal cluster #2 of non-pregnant NSG-LIRKO mice compared to non-pregnant NSG-Lox mice (Insulin Resistant model)

List of selected pathways analyzed by ConsensusPathDB.
The p-value is calculated according to the hypergeometric test based on the number of physical entities present in both the predefined set and user-specified list of physical entities. Reference is cited in Methods.

UPREGULATED pathways	set size	candidates contained	p value	q value	Pathways database
Peptide chain elongation	100	6 (6.0%)	7.25E-09	1.03E-06	Reactome
Eukaryotic Translation Elongation	105	6 (5.7%)	9.74E-09	1.03E-06	Reactome
Cytoplasmic Ribosomal Proteins	88	5 (5.7%)	2.01E-07	1.42E-05	Wikipathways
Eukaryotic Translation Termination	103	5 (4.9%)	4.43E-07	1.72E-05	Reactome
Selenocysteine synthesis	103	5 (4.9%)	4.43E-07	1.72E-05	Reactome
Nonsense Mediated Decay (NMD) independent of the Exon Junction Complex (EJC)	105	5 (4.8%)	4.88E-07	1.72E-05	Reactome
Response of EIF2AK4 (GCN2) to amino acid deficiency	111	5 (4.5%)	6.44E-07	1.77E-05	Reactome
Formation of a pool of free 40S subunits	113	5 (4.4%)	7.04E-07	1.77E-05	Reactome
Nonsense Mediated Decay (NMD) enhanced by the Exon Junction Complex (EJC)	117	5 (4.3%)	8.37E-07	1.77E-05	Reactome
Nonsense-Mediated Decay (NMD)	117	5 (4.3%)	8.37E-07	1.77E-05	Reactome
L13a-mediated translational silencing of Ceruloplasmin expression	123	5 (4.1%)	1.07E-06	1.82E-05	Reactome
SRP-dependent cotranslational protein targeting to membrane	123	5 (4.1%)	1.07E-06	1.82E-05	Reactome
GTP hydrolysis and joining of the 60S ribosomal subunit	124	5 (4.0%)	1.12E-06	1.82E-05	Reactome
Selenoamino acid metabolism	128	5 (3.9%)	1.31E-06	1.94E-05	Reactome
Cap-dependent Translation Initiation	131	5 (3.8%)	1.47E-06	1.94E-05	Reactome
Eukaryotic Translation Initiation	131	5 (3.8%)	1.47E-06	1.94E-05	Reactome
Cellular response to starvation	145	5 (3.4%)	2.42E-06	3.02E-05	Reactome
Ribosome - Homo sapiens (human)	158	5 (3.2%)	3.69E-06	4.35E-05	KEGG
Translation	307	6 (2.0%)	5.51E-06	6.15E-05	Reactome
Metabolism of amino acids and derivatives	339	6 (1.8%)	9.73E-06	7.54E-05	Reactome
Spironolactone Action Pathway	33	3 (9.1%)	1.74E-05	7.54E-05	SMPDB
Eplerenone Action Pathway	33	3 (9.1%)	1.74E-05	7.54E-05	SMPDB
Triamterene Action Pathway	33	3 (9.1%)	1.74E-05	7.54E-05	SMPDB
Amiloride Action Pathway	33	3 (9.1%)	1.74E-05	7.54E-05	SMPDB
Torsemide Action Pathway	33	3 (9.1%)	1.74E-05	7.54E-05	SMPDB
Iminoglycinuria	33	3 (9.1%)	1.74E-05	7.54E-05	SMPDB
Lysinuric Protein Intolerance	33	3 (9.1%)	1.74E-05	7.54E-05	SMPDB
Blue diaper syndrome	33	3 (9.1%)	1.74E-05	7.54E-05	SMPDB
Lysinuric protein intolerance (LPI)	33	3 (9.1%)	1.74E-05	7.54E-05	SMPDB
Cystinuria	33	3 (9.1%)	1.74E-05	7.54E-05	SMPDB
Hartnup Disorder	33	3 (9.1%)	1.74E-05	7.54E-05	SMPDB
Glucose Transporter Defect (SGLT2)	33	3 (9.1%)	1.74E-05	7.54E-05	SMPDB
Kidney Function	33	3 (9.1%)	1.74E-05	7.54E-05	SMPDB
Glucose Transporter Defect (SGLT2)	33	3 (9.1%)	1.74E-05	7.54E-05	SMPDB
Quinethazone Action Pathway	33	3 (9.1%)	1.74E-05	7.54E-05	SMPDB
Bendroflumethiazide Action Pathway	33	3 (9.1%)	1.74E-05	7.54E-05	SMPDB
Chlorthalidone Action Pathway	33	3 (9.1%)	1.74E-05	7.54E-05	SMPDB
Trichlormethiazide Action Pathway	33	3 (9.1%)	1.74E-05	7.54E-05	SMPDB
Indapamide Action Pathway	33	3 (9.1%)	1.74E-05	7.54E-05	SMPDB
Metolazone Action Pathway	33	3 (9.1%)	1.74E-05	7.54E-05	SMPDB
Hydrochlorothiazide Action Pathway	33	3 (9.1%)	1.74E-05	7.54E-05	SMPDB
Cyclothiazide Action Pathway	33	3 (9.1%)	1.74E-05	7.54E-05	SMPDB
Hydroflumethiazide Action Pathway	33	3 (9.1%)	1.74E-05	7.54E-05	SMPDB
Bumetanide Action Pathway	33	3 (9.1%)	1.74E-05	7.54E-05	SMPDB
Ethacrynic Acid Action Pathway	33	3 (9.1%)	1.74E-05	7.54E-05	SMPDB
Furosemide Action Pathway	33	3 (9.1%)	1.74E-05	7.54E-05	SMPDB
Polythiazide Action Pathway	33	3 (9.1%)	1.74E-05	7.54E-05	SMPDB
Methyclothiazide Action Pathway	33	3 (9.1%)	1.74E-05	7.54E-05	SMPDB
Chlorothiazide Action Pathway	33	3 (9.1%)	1.74E-05	7.54E-05	SMPDB
Coronavirus disease - COVID-19 - Homo sapiens (human)	232	5 (2.2%)	2.34E-05	9.92E-05	KEGG
Proximal tubule transport	57	3 (5.3%)	9.12E-05	0.000379	Wikipathways
Lactose Degradation	11	2 (18.2%)	0.000127	0.000508	SMPDB
Lactose Intolerance	11	2 (18.2%)	0.000127	0.000508	SMPDB
Cellular responses to stress	554	6 (1.1%)	0.000151	0.000587	Reactome
Trehalose Degradation	12	2 (16.7%)	0.000152	0.000587	SMPDB
Cellular responses to external stimuli	569	6 (1.1%)	0.000175	0.000663	Reactome
Potential therapeutics for SARS	80	3 (3.8%)	0.00025	0.000931	Reactome
Insulin secretion - Homo sapiens (human)	86	3 (3.5%)	0.00031	0.00112	KEGG
Metabolism	1954	10 (0.5%)	0.000311	0.00112	Reactome
Protein digestion and absorption - Homo sapiens (human)	103	3 (2.9%)	0.000527	0.00183	KEGG
Chaperone Mediated Autophagy	22	2 (9.1%)	0.000528	0.00183	Reactome
Proximal tubule bicarbonate reclamation - Homo sapiens (human)	23	2 (8.7%)	0.000577	0.00188	KEGG
SARS-CoV Infections	111	3 (2.7%)	0.000655	0.00188	Reactome
Levomethadyl Acetate Action Action Pathway	29	2 (6.9%)	0.000921	0.00188	SMPDB
Levallorphan Action Pathway	29	2 (6.9%)	0.000921	0.00188	SMPDB
Dimethylthiambutene Action Pathway	29	2 (6.9%)	0.000921	0.00188	SMPDB
Ethylmorphine Action Pathway	29	2 (6.9%)	0.000921	0.00188	SMPDB
Pentazocine Action Pathway	29	2 (6.9%)	0.000921	0.00188	SMPDB
Naltrexone Action Pathway	29	2 (6.9%)	0.000921	0.00188	SMPDB
Buprenorphine Action Pathway	29	2 (6.9%)	0.000921	0.00188	SMPDB
Alvimopan Action Pathway	29	2 (6.9%)	0.000921	0.00188	SMPDB
Naloxone Action Pathway	29	2 (6.9%)	0.000921	0.00188	SMPDB
Dihydromorphine Action Pathway	29	2 (6.9%)	0.000921	0.00188	SMPDB
Ketobemidone Action Pathway	29	2 (6.9%)	0.000921	0.00188	SMPDB
Levorphanol Action Pathway	29	2 (6.9%)	0.000921	0.00188	SMPDB
Propoxyphene Action Pathway	29	2 (6.9%)	0.000921	0.00188	SMPDB
Tramadol Action Action Pathway	29	2 (6.9%)	0.000921	0.00188	SMPDB
Diphenoxylate Action Pathway	29	2 (6.9%)	0.000921	0.00188	SMPDB
Anileridine Action Pathway	29	2 (6.9%)	0.000921	0.00188	SMPDB
Oxycodone Action Pathway	29	2 (6.9%)	0.000921	0.00188	SMPDB
Alfentanil Action Pathway	29	2 (6.9%)	0.000921	0.00188	SMPDB
Oxymorphone Action Pathway	29	2 (6.9%)	0.000921	0.00188	SMPDB
Hydrocodone Action Pathway	29	2 (6.9%)	0.000921	0.00188	SMPDB
Hydromorphone Action Pathway	29	2 (6.9%)	0.000921	0.00188	SMPDB
Sufentanil Action Pathway	29	2 (6.9%)	0.000921	0.00188	SMPDB
Remifentanil Action Pathway	29	2 (6.9%)	0.000921	0.00188	SMPDB

Fentanyl Action Pathway	29	2 (6.9%)	0.000921	0.00188	**SMPDB**
Carfentanil Action Pathway	29	2 (6.9%)	0.000921	0.00188	**SMPDB**
3-Methylthiofentanyl Action Pathway	29	2 (6.9%)	0.000921	0.00188	**SMPDB**
Methadyl Acetate Action Pathway	29	2 (6.9%)	0.000921	0.00188	**SMPDB**
Dezocine Action Pathway	29	2 (6.9%)	0.000921	0.00188	**SMPDB**
Mepivacaine Action Pathway	29	2 (6.9%)	0.000921	0.00188	**SMPDB**
Chloroprocaine Action Pathway	29	2 (6.9%)	0.000921	0.00188	**SMPDB**
Dibucaine Action Pathway	29	2 (6.9%)	0.000921	0.00188	**SMPDB**
Levobupivacaine Action Pathway	29	2 (6.9%)	0.000921	0.00188	**SMPDB**
Benzocaine Action Pathway	29	2 (6.9%)	0.000921	0.00188	**SMPDB**
Bupivacaine Action Pathway	29	2 (6.9%)	0.000921	0.00188	**SMPDB**
Oxybuprocaine Action Pathway	29	2 (6.9%)	0.000921	0.00188	**SMPDB**
Prilocaine Action Pathway	29	2 (6.9%)	0.000921	0.00188	**SMPDB**
Procaine Action Pathway	29	2 (6.9%)	0.000921	0.00188	**SMPDB**
Proparacaine Action Pathway	29	2 (6.9%)	0.000921	0.00188	**SMPDB**
Ropivacaine Action Pathway	29	2 (6.9%)	0.000921	0.00188	**SMPDB**
Cocaine Action Pathway	29	2 (6.9%)	0.000921	0.00188	**SMPDB**
Escitalopram Action Pathway	29	2 (6.9%)	0.000921	0.00188	**SMPDB**
Nalbuphine Action Pathway	30	2 (6.7%)	0.000986	0.00197	**SMPDB**
Desipramine Action Pathway	30	2 (6.7%)	0.000986	0.00197	**SMPDB**
Lidocaine (Local Anaesthetic) Action Pathway	31	2 (6.5%)	0.00105	0.00209	**SMPDB**
Heroin Action Pathway	32	2 (6.2%)	0.00112	0.00218	**SMPDB**
Codeine Action Pathway	32	2 (6.2%)	0.00112	0.00218	**SMPDB**
Imipramine Action Pathway	33	2 (6.1%)	0.00119	0.0023	**SMPDB**
Fluoxetine Action Pathway	34	2 (5.9%)	0.00127	0.00242	**SMPDB**
Nicotine Action Pathway	36	2 (5.6%)	0.00142	0.00264	**SMPDB**
Methadone Action Pathway	36	2 (5.6%)	0.00142	0.00264	**SMPDB**
Citalopram Action Pathway	36	2 (5.6%)	0.00142	0.00264	**SMPDB**
Metabolism of proteins	1935	9 (0.5%)	0.00149	0.00274	**Reactome**
Aldosterone-regulated sodium reabsorption - Homo sapiens (human)	37	2 (5.4%)	0.0015	0.00274	**KEGG**
Metabolism of RNA	584	5 (0.9%)	0.00173	0.00313	**Reactome**
Ion homeostasis	44	2 (4.5%)	0.00212	0.00377	**Reactome**
Morphine Action Pathway	44	2 (4.5%)	0.00212	0.00377	**SMPDB**
Carbohydrate digestion and absorption - Homo sapiens (human)	47	2 (4.3%)	0.00241	0.00423	**KEGG**
Ion transport by P-type ATPases	47	2 (4.3%)	0.00241	0.00423	**Reactome**
Endocrine and other factor-regulated calcium reabsorption - Homo sapiens (human)	53	2 (3.8%)	0.00306	0.00505	**KEGG**
Mineral absorption - Homo sapiens (human)	59	2 (3.4%)	0.00377	0.00505	**KEGG**
Formation of the ternary complex, and subsequently, the 43S complex	59	2 (3.4%)	0.00377	0.00505	**Reactome**
Bopindolol Action Pathway	61	2 (3.3%)	0.00403	0.00505	**SMPDB**
Timolol Action Pathway	61	2 (3.3%)	0.00403	0.00505	**SMPDB**
Carteolol Action Pathway	61	2 (3.3%)	0.00403	0.00505	**SMPDB**
Bevantolol Action Pathway	61	2 (3.3%)	0.00403	0.00505	**SMPDB**
Practolol Action Pathway	61	2 (3.3%)	0.00403	0.00505	**SMPDB**
Dobutamine Action Pathway	61	2 (3.3%)	0.00403	0.00505	**SMPDB**
Isoprenaline Action Pathway	61	2 (3.3%)	0.00403	0.00505	**SMPDB**
Arbutamine Action Pathway	61	2 (3.3%)	0.00403	0.00505	**SMPDB**
Levobunolol Action Pathway	61	2 (3.3%)	0.00403	0.00505	**SMPDB**
Metipranolol Action Pathway	61	2 (3.3%)	0.00403	0.00505	**SMPDB**
Sotalol Action Pathway	61	2 (3.3%)	0.00403	0.00505	**SMPDB**
Epinephrine Action Pathway	61	2 (3.3%)	0.00403	0.00505	**SMPDB**
Betaxolol Action Pathway	61	2 (3.3%)	0.00403	0.00505	**SMPDB**
Atenolol Action Pathway	61	2 (3.3%)	0.00403	0.00505	**SMPDB**
Alprenolol Action Pathway	61	2 (3.3%)	0.00403	0.00505	**SMPDB**
Acebutolol Action Pathway	61	2 (3.3%)	0.00403	0.00505	**SMPDB**
Propranolol Action Pathway	61	2 (3.3%)	0.00403	0.00505	**SMPDB**
Pindolol Action Pathway	61	2 (3.3%)	0.00403	0.00505	**SMPDB**
Penbutolol Action Pathway	61	2 (3.3%)	0.00403	0.00505	**SMPDB**
Oxprenolol Action Pathway	61	2 (3.3%)	0.00403	0.00505	**SMPDB**
Metoprolol Action Pathway	61	2 (3.3%)	0.00403	0.00505	**SMPDB**
Esmolol Action Pathway	61	2 (3.3%)	0.00403	0.00505	**SMPDB**
Bisoprolol Action Pathway	61	2 (3.3%)	0.00403	0.00505	**SMPDB**
Bupranolol Action Pathway	61	2 (3.3%)	0.00403	0.00505	**SMPDB**
Nebivolol Action Pathway	61	2 (3.3%)	0.00403	0.00505	**SMPDB**
Nadolol Action Pathway	61	2 (3.3%)	0.00403	0.00505	**SMPDB**
Muscle/Heart Contraction	61	2 (3.3%)	0.00403	0.00505	**SMPDB**
Diltiazem Action Pathway	61	2 (3.3%)	0.00403	0.00505	**SMPDB**
Amlodipine Action Pathway	61	2 (3.3%)	0.00403	0.00505	**SMPDB**
Verapamil Action Pathway	61	2 (3.3%)	0.00403	0.00505	**SMPDB**
Nitrendipine Action Pathway	61	2 (3.3%)	0.00403	0.00505	**SMPDB**
Nisoldipine Action Pathway	61	2 (3.3%)	0.00403	0.00505	**SMPDB**
Nimodipine Action Pathway	61	2 (3.3%)	0.00403	0.00505	**SMPDB**
Isradipine Action Pathway	61	2 (3.3%)	0.00403	0.00505	**SMPDB**
Nifedipine Action Pathway	61	2 (3.3%)	0.00403	0.00505	**SMPDB**
Felodipine Action Pathway	61	2 (3.3%)	0.00403	0.00505	**SMPDB**
Quinidine Action Pathway	61	2 (3.3%)	0.00403	0.00505	**SMPDB**
Procainamide (Antiarrhythmic) Action Pathway	61	2 (3.3%)	0.00403	0.00505	**SMPDB**
Disopyramide Action Pathway	61	2 (3.3%)	0.00403	0.00505	**SMPDB**
Fosphenytoin (Antiarrhythmic) Action Pathway	61	2 (3.3%)	0.00403	0.00505	**SMPDB**
Mexiletine Action Pathway	61	2 (3.3%)	0.00403	0.00505	**SMPDB**
Tocainide Action Pathway	61	2 (3.3%)	0.00403	0.00505	**SMPDB**
Flecainide Action Pathway	61	2 (3.3%)	0.00403	0.00505	**SMPDB**
Amiodarone Action Pathway	61	2 (3.3%)	0.00403	0.00505	**SMPDB**
Ibutilide Action Pathway	61	2 (3.3%)	0.00403	0.00505	**SMPDB**
Carvedilol Action Pathway	62	2 (3.2%)	0.00416	0.00516	**SMPDB**
Labetalol Action Pathway	62	2 (3.2%)	0.00416	0.00516	**SMPDB**
Lidocaine (Antiarrhythmic) Action Pathway	63	2 (3.2%)	0.00429	0.00529	**SMPDB**
cAMP signaling pathway - Homo sapiens (human)	216	3 (1.4%)	0.0044	0.0054	**KEGG**
Ribosomal scanning and start codon recognition	66	2 (3.0%)	0.0047	0.00569	**Reactome**
Translation initiation complex formation	66	2 (3.0%)	0.0047	0.00569	**Reactome**
Activation of the mRNA upon binding of the cap-binding complex and eIFs, and subsequent binding	67	2 (3.0%)	0.00484	0.00583	**Reactome**
Thyroid hormone synthesis - Homo sapiens (human)	75	2 (2.7%)	0.00603	0.00722	**KEGG**
Gastric acid secretion - Homo sapiens (human)	76	2 (2.6%)	0.00619	0.00737	**KEGG**
Phenytoin (Antiarrhythmic) Action Pathway	82	2 (2.4%)	0.00717	0.00849	**SMPDB**

Cardiac muscle contraction - Homo sapiens (human)	87	2 (2.3%)	0.00804	0.00947	**KEGG**
Bile secretion - Homo sapiens (human)	90	2 (2.2%)	0.00858	0.0101	**KEGG**
Salivary secretion - Homo sapiens (human)	93	2 (2.2%)	0.00914	0.0106	**KEGG**

**DOWNGULATED pathways**	**set size**	**candidates contained**	**p value**	**q value**	**Pathways database**
Miscellaneous transport and binding events	24	7 (29.2%)	1.64E-05	0.0249	**Reactome**
Phenylalanine and tyrosine metabolism	13	5 (38.5%)	6.32E-05	0.0481	**Reactome**
Tyrosine catabolism	5	3 (60.0%)	0.000454	0.079	**Reactome**
tyrosine degradation	5	3 (60.0%)	0.000454	0.079	**HumanCyc**
Synthesis of GDP-mannose	5	3 (60.0%)	0.000454	0.079	**Reactome**
Phenylalanine and Tyrosine Metabolism	11	4 (36.4%)	0.000467	0.079	**SMPDB**
Phenylketonuria	11	4 (36.4%)	0.000467	0.079	**SMPDB**
Tyrosinemia Type 3 (TYRO3)	11	4 (36.4%)	0.000467	0.079	**SMPDB**
Tyrosinemia Type 2 (or Richner-Hanhart syndrome)	11	4 (36.4%)	0.000467	0.079	**SMPDB**
GDP-mannose biosynthesis	6	3 (50.0%)	0.000883	0.134	**HumanCyc**
RNA Polymerase I Promoter Opening	2	2 (100.0%)	0.00132	0.183	**Reactome**
Amino sugar and nucleotide sugar metabolism - Homo sapiens (human)	48	7 (14.6%)	0.00164	0.201	**KEGG**
Glycosphingolipid biosynthesis - globo and isoglobo series - Homo sapiens (human)	15	4 (26.7%)	0.00172	0.201	**KEGG**
Metabolism of RNA	584	35 (6.0%)	0.00255	0.218	**Reactome**
Apoptosis-related network due to altered Notch3 in ovarian cancer	53	7 (13.2%)	0.00293	0.218	**Wikipathways**
Sepiapterin reductase deficiency	9	3 (33.3%)	0.00342	0.218	**SMPDB**
Segawa syndrome	9	3 (33.3%)	0.00342	0.218	**SMPDB**
Pterine Biosynthesis	9	3 (33.3%)	0.00342	0.218	**SMPDB**
Dopa-responsive dystonia	9	3 (33.3%)	0.00342	0.218	**SMPDB**
Hyperphenylalaniemia due to guanosine triphosphate cyclohydrolase deficiency	9	3 (33.3%)	0.00342	0.218	**SMPDB**
Hyperphenylalaninemia due to 6-pyruvoyltetrahydropterin synthase deficiency (ptps)	9	3 (33.3%)	0.00342	0.218	**SMPDB**
Hyperphenylalaninemia due to dhpr-deficiency	9	3 (33.3%)	0.00342	0.218	**SMPDB**
Glycosphingolipid biosynthesis - globoseries	18	4 (22.2%)	0.00353	0.218	**EHMN**
Loss of Nlp from mitotic centrosomes	70	8 (11.4%)	0.00379	0.218	**Reactome**
Loss of proteins required for interphase microtubule organization from the centrosome	70	8 (11.4%)	0.00379	0.218	**Reactome**
Chylomicron clearance	3	2 (66.7%)	0.00387	0.218	**Reactome**
Intestinal hexose absorption	3	2 (66.7%)	0.00387	0.218	**Reactome**
Gene expression (Transcription)	1439	71 (4.9%)	0.00457	0.234	**Reactome**
Regulation of PLK1 Activity at G2/M Transition	88	9 (10.2%)	0.00465	0.234	**Reactome**
AURKA Activation by TPX2	73	8 (11.0%)	0.00491	0.234	**Reactome**
TP53 Regulates Transcription of Cell Death Genes	44	6 (13.6%)	0.00492	0.234	**Reactome**
Galactose metabolism	44	6 (13.6%)	0.00492	0.234	**EHMN**
Interconversion of nucleotide di- and triphosphates	32	5 (15.6%)	0.00559	0.258	**Reactome**
Autosomal recessive Osteopetrosis pathways	11	3 (27.3%)	0.00636	0.278	**Wikipathways**
Prion disease pathway	33	5 (15.2%)	0.0064	0.278	**Wikipathways**
Transcriptional regulation of white adipocyte differentiation	47	6 (12.8%)	0.00683	0.288	**Reactome**
Fc-epsilon receptor I signaling in mast cells	62	7 (11.3%)	0.00708	0.288	**PID**
superpathway of pyrimidine deoxyribonucleotides *de novo* biosynthesis	22	4 (18.2%)	0.00753	0.288	**HumanCyc**
Intestinal absorption	4	2 (50.0%)	0.00756	0.288	**Reactome**
Ceramide signaling pathway	48	6 (12.5%)	0.00757	0.288	**PID**
TP53 Regulates Transcription of Caspase Activators and Caspases	12	3 (25.0%)	0.00825	0.292	**Reactome**
Tetrahydrobiopterin (BH4) synthesis, recycling, salvage and regulation	12	3 (25.0%)	0.00825	0.292	**Reactome**
Trihydroxycoprostanoyl-CoA beta-oxidation	12	3 (25.0%)	0.00825	0.292	**EHMN**
Anchoring of the basal body to the plasma membrane	97	9 (9.3%)	0.00875	0.299	**Reactome**
Renin-angiotensin system - Homo sapiens (human)	23	4 (17.4%)	0.00886	0.299	**KEGG**
Recruitment of NuMA to mitotic centrosomes	81	8 (9.9%)	0.00915	0.299	**Reactome**
Nuclear import of Rev protein	36	5 (13.9%)	0.00929	0.299	**Reactome**
Hepatitis B infection	152	12 (7.9%)	0.00961	0.299	**Wikipathways**
Recruitment of mitotic centrosome proteins and complexes	82	8 (9.8%)	0.00982	0.299	**Reactome**
Centrosome maturation	82	8 (9.8%)	0.00982	0.299	**Reactome**

Supplemental Table 4. Selected differentially regulated pathways in the ductal cluster #2 of pregnant NSG-LIRKO mice compared to non-pregnant NSG-Lox mice (Combined model).

List of selected pathways analyzed by ConsensusPathDB.
The p-value is calculated according to the hypergeometric test based on the number of physical entities present in both the predefined set and user-specified list of physical entities. Reference is cited in Methods.

UPREGULATED pathways	set size	candidates contained	p value	q value	Pathways database
Spironolactone Action Pathway	33	2 (6.1%)	3.50E-05	3.86E-05	SMPDB
Eplerenone Action Pathway	33	2 (6.1%)	3.50E-05	3.86E-05	SMPDB
Triamterene Action Pathway	33	2 (6.1%)	3.50E-05	3.86E-05	SMPDB
Amiloride Action Pathway	33	2 (6.1%)	3.50E-05	3.86E-05	SMPDB
Torsemide Action Pathway	33	2 (6.1%)	3.50E-05	3.86E-05	SMPDB
Iminoglycinuria	33	2 (6.1%)	3.50E-05	3.86E-05	SMPDB
Lysinuric Protein Intolerance	33	2 (6.1%)	3.50E-05	3.86E-05	SMPDB
Blue diaper syndrome	33	2 (6.1%)	3.50E-05	3.86E-05	SMPDB
Lysinuric protein intolerance (LPI)	33	2 (6.1%)	3.50E-05	3.86E-05	SMPDB
Cystinuria	33	2 (6.1%)	3.50E-05	3.86E-05	SMPDB
Hartnup Disorder	33	2 (6.1%)	3.50E-05	3.86E-05	SMPDB
Glucose Transporter Defect (SGLT2)	33	2 (6.1%)	3.50E-05	3.86E-05	SMPDB
Kidney Function	33	2 (6.1%)	3.50E-05	3.86E-05	SMPDB
Glucose Transporter Defect (SGLT2)	33	2 (6.1%)	3.50E-05	3.86E-05	SMPDB
Quinethazone Action Pathway	33	2 (6.1%)	3.50E-05	3.86E-05	SMPDB
Bendroflumethiazide Action Pathway	33	2 (6.1%)	3.50E-05	3.86E-05	SMPDB
Chlorthalidone Action Pathway	33	2 (6.1%)	3.50E-05	3.86E-05	SMPDB
Trichlormethiazide Action Pathway	33	2 (6.1%)	3.50E-05	3.86E-05	SMPDB
Indapamide Action Pathway	33	2 (6.1%)	3.50E-05	3.86E-05	SMPDB
Metolazone Action Pathway	33	2 (6.1%)	3.50E-05	3.86E-05	SMPDB
Hydrochlorothiazide Action Pathway	33	2 (6.1%)	3.50E-05	3.86E-05	SMPDB
Cyclothiazide Action Pathway	33	2 (6.1%)	3.50E-05	3.86E-05	SMPDB
Hydroflumethiazide Action Pathway	33	2 (6.1%)	3.50E-05	3.86E-05	SMPDB
Bumetanide Action Pathway	33	2 (6.1%)	3.50E-05	3.86E-05	SMPDB
Ethacrynic Acid Action Pathway	33	2 (6.1%)	3.50E-05	3.86E-05	SMPDB
Furosemide Action Pathway	33	2 (6.1%)	3.50E-05	3.86E-05	SMPDB
Polythiazide Action Pathway	33	2 (6.1%)	3.50E-05	3.86E-05	SMPDB
Methyclothiazide Action Pathway	33	2 (6.1%)	3.50E-05	3.86E-05	SMPDB
Chlorothiazide Action Pathway	33	2 (6.1%)	3.50E-05	3.86E-05	SMPDB
Proximal tubule transport	57	2 (3.5%)	0.000106	0.000113	Wikipathways
Protein digestion and absorption - Homo sapiens (human)	103	2 (1.9%)	0.000346	0.000357	KEGG

DOWNGULATED pathways	set size	candidates contained	p value	q value	Pathways database
EGF-EGFR signaling pathway	162	18 (11.1%)	8.63E-05	0.105	Wikipathways
Cell-cell junction organization	64	10 (15.6%)	0.000211	0.105	Reactome
Beta-oxidation of pristanoyl-CoA	9	4 (44.4%)	0.000273	0.105	Reactome
Phytanic acid peroxisomal oxidation	16	5 (31.2%)	0.000306	0.105	EHMN
EGFR1	455	34 (7.5%)	0.000341	0.105	NetPath
Trihydroxycoprostanoyl-CoA beta-oxidation	12	4 (33.3%)	0.000974	0.219	EHMN
Peroxisomal lipid metabolism	30	6 (20.0%)	0.00105	0.219	Reactome
NO metabolism in cystic fibrosis	13	4 (30.8%)	0.00136	0.219	Wikipathways
Fc gamma R-mediated phagocytosis - Homo sapiens (human)	97	11 (11.5%)	0.00155	0.219	KEGG
RNA Polymerase I Promoter Opening	2	2 (100.0%)	0.0016	0.219	Reactome
Notch Signaling	45	7 (15.6%)	0.00192	0.219	Wikipathways
SUMO E3 ligases SUMOylate target proteins	177	16 (9.0%)	0.00201	0.219	Reactome
snRNP Assembly	24	5 (20.8%)	0.00228	0.219	Reactome
Metabolism of non-coding RNA	24	5 (20.8%)	0.00228	0.219	Reactome
Notch signaling pathway - Homo sapiens (human)	59	8 (13.6%)	0.0023	0.219	KEGG
angiotensin ii mediated activation of jnk pathway via pyk2 dependent signaling	35	6 (17.1%)	0.00242	0.219	BioCarta
SUMOylation of transcription cofactors	47	7 (14.9%)	0.00248	0.219	Reactome
SUMOylation	182	16 (8.8%)	0.00267	0.219	Reactome
Fibroblast growth factor-1	74	9 (12.2%)	0.0027	0.219	NetPath
Cell junction organization	89	10 (11.2%)	0.0029	0.224	Reactome
Fc-epsilon receptor I signaling in mast cells	62	8 (12.9%)	0.00316	0.225	PID
Tight junction - Homo sapiens (human)	169	15 (8.9%)	0.00327	0.225	KEGG
mechanism of gene regulation by peroxisome proliferators via ppara	52	7 (13.5%)	0.00445	0.225	BioCarta
Sepiapterin reductase deficiency	9	3 (33.3%)	0.00448	0.225	SMPDB
Segawa syndrome	9	3 (33.3%)	0.00448	0.225	SMPDB
Pterine Biosynthesis	9	3 (33.3%)	0.00448	0.225	SMPDB
Dopa-responsive dystonia	9	3 (33.3%)	0.00448	0.225	SMPDB
Hyperphenylalaniemia due to guanosine triphosphate cyclohydrolase deficiency	9	3 (33.3%)	0.00448	0.225	SMPDB
Hyperphenylalaninemia due to 6-pyruvoyltetrahydropterin synthase deficiency (ptps)	9	3 (33.3%)	0.00448	0.225	SMPDB
Hyperphenylalaninemia due to dhpr-deficiency	9	3 (33.3%)	0.00448	0.225	SMPDB
One-carbon metabolism	28	5 (17.9%)	0.00463	0.225	Wikipathways
fatty acid β-oxidation (unsaturated, odd number)	3	2 (66.7%)	0.00468	0.225	HumanCyc
Axon guidance - Homo sapiens (human)	182	15 (8.2%)	0.00649	0.278	KEGG
Global Genome Nucleotide Excision Repair (GG-NER)	70	8 (11.4%)	0.00669	0.278	Reactome
Formation of Incision Complex in GG-NER	43	6 (14.0%)	0.00694	0.278	Reactome
Adherens junctions interactions	31	5 (16.1%)	0.00725	0.278	Reactome
Tight junction interactions	31	5 (16.1%)	0.00725	0.278	Reactome
Folate metabolism	31	5 (16.1%)	0.00725	0.278	INOH
One carbon pool by folate - Homo sapiens (human)	20	4 (20.0%)	0.00739	0.278	KEGG
3-phosphoinositide degradation	20	4 (20.0%)	0.00739	0.278	HumanCyc
S1P1 pathway	20	4 (20.0%)	0.00739	0.278	PID
rho cell motility signaling pathway	32	5 (15.6%)	0.00831	0.305	BioCarta
Cholesterol biosynthesis via desmosterol	4	2 (50.0%)	0.0091	0.318	Reactome
Cholesterol biosynthesis via lathosterol	4	2 (50.0%)	0.0091	0.318	Reactome
IL6	74	8 (10.8%)	0.0093	0.318	NetPath
Prion disease pathway	33	5 (15.2%)	0.00948	0.318	Wikipathways
ErbB1 downstream signaling	106	10 (9.4%)	0.00995	0.324	PID

Supplemental Table 5. Selected differentially regulated pathways in the ductal cluster #4 of pregnant NSG-Lox mice compared to non-pregnant NSG-Lox mice (Pregnancy model).

List of selected pathways analyzed by ConsensusPathDB.
The p-value is calculated according to the hypergeometric test based on the number of physical entities present in both the predefined set and user-specified list of physical entities. Reference is cited in Methods.

UPREGULATED pathways	set size	candidates contained	p value	q value	Pathways database
Cytoplasmic Ribosomal Proteins	88	9 (10.2%)	1.11E-14	2.30E-12	Wikipathways
Peptide chain elongation	100	9 (9.0%)	3.64E-14	2.30E-12	Reactome
Eukaryotic Translation Termination	103	9 (8.7%)	4.79E-14	2.30E-12	Reactome
Selenocysteine synthesis	103	9 (8.7%)	4.79E-14	2.30E-12	Reactome
Nonsense Mediated Decay (NMD) independent of the Exon Junc	105	9 (8.6%)	5.73E-14	2.30E-12	Reactome
Eukaryotic Translation Elongation	105	9 (8.6%)	5.73E-14	2.30E-12	Reactome
Response of EIF2AK4 (GCN2) to amino acid deficiency	111	9 (8.1%)	9.58E-14	3.30E-12	Reactome
Formation of a pool of free 40S subunits	113	9 (8.0%)	1.13E-13	3.40E-12	Reactome
Nonsense Mediated Decay (NMD) enhanced by the Exon Junctio	117	9 (7.7%)	1.56E-13	3.75E-12	Reactome
Nonsense-Mediated Decay (NMD)	117	9 (7.7%)	1.56E-13	3.75E-12	Reactome
SRP-dependent cotranslational protein targeting to membrane	123	9 (7.3%)	2.47E-13	4.92E-12	Reactome
L13a-mediated translational silencing of Ceruloplasmin expressio	123	9 (7.3%)	2.47E-13	4.92E-12	Reactome
GTP hydrolysis and joining of the 60S ribosomal subunit	124	9 (7.3%)	2.66E-13	4.92E-12	Reactome
Selenoamino acid metabolism	128	9 (7.0%)	3.56E-13	6.12E-12	Reactome
Cap-dependent Translation Initiation	131	9 (6.9%)	4.40E-13	6.62E-12	Reactome
Eukaryotic Translation Initiation	131	9 (6.9%)	4.40E-13	6.62E-12	Reactome
Cellular response to starvation	145	9 (6.2%)	1.11E-12	1.58E-11	Reactome
Ribosome - Homo sapiens (human)	158	9 (5.7%)	2.43E-12	3.25E-11	KEGG
Coronavirus disease - COVID-19 - Homo sapiens (human)	232	9 (3.9%)	7.46E-11	9.46E-10	KEGG
Cellular responses to stress	554	11 (2.0%)	4.49E-10	5.42E-09	Reactome
Cellular responses to external stimuli	569	11 (1.9%)	5.97E-10	6.85E-09	Reactome
Translation	307	9 (2.9%)	9.33E-10	1.02E-08	Reactome
Metabolism of amino acids and derivatives	339	9 (2.7%)	2.23E-09	2.34E-08	Reactome
Pancreatic secretion - Homo sapiens (human)	102	6 (5.9%)	1.50E-08	1.51E-07	KEGG
Metabolism	1954	15 (0.8%)	3.99E-08	3.84E-07	Reactome
Metabolism of RNA	584	9 (1.5%)	2.43E-07	2.25E-06	Reactome
Cardiac muscle contraction - Homo sapiens (human)	87	5 (5.7%)	3.11E-07	2.78E-06	KEGG
Digestion	22	3 (13.6%)	6.61E-06	5.69E-05	Reactome
Digestion and absorption	26	3 (11.5%)	1.11E-05	9.23E-05	Reactome
Protein digestion and absorption - Homo sapiens (human)	103	4 (3.9%)	2.58E-05	0.000207	KEGG
Digestion of dietary lipid	7	2 (28.6%)	5.86E-05	0.000455	Reactome
Carbohydrate digestion and absorption - Homo sapiens (human)	47	3 (6.4%)	6.77E-05	0.00051	KEGG
Formation of the ternary complex, and subsequently, the 43S cor	59	3 (5.1%)	0.000134	0.000949	Reactome
Mineral absorption - Homo sapiens (human)	59	3 (5.1%)	0.000134	0.000949	KEGG
Lactose Degradation	11	2 (18.2%)	0.000153	0.00102	SMPDB
Lactose Intolerance	11	2 (18.2%)	0.000153	0.00102	SMPDB
Cytoprotection by HMOX1	65	3 (4.6%)	0.000179	0.00113	Reactome
Trehalose Degradation	12	2 (16.7%)	0.000183	0.00113	SMPDB
Ribosomal scanning and start codon recognition	66	3 (4.5%)	0.000187	0.00113	Reactome
Translation initiation complex formation	66	3 (4.5%)	0.000187	0.00113	Reactome
Activation of the mRNA upon binding of the cap-binding complex	67	3 (4.5%)	0.000196	0.00115	Reactome
Neuroinflammation	14	2 (14.3%)	0.000252	0.00145	Wikipathways
Respiratory electron transport	89	3 (3.4%)	0.000452	0.00253	Reactome
Salivary secretion - Homo sapiens (human)	93	3 (3.2%)	0.000514	0.00272	KEGG
Cellular response to chemical stress	99	3 (3.0%)	0.000618	0.00272	Reactome
PINK1-PRKN Mediated Mitophagy	22	2 (9.1%)	0.000634	0.00272	Reactome
Electron Transport Chain (OXPHOS system in mitochondria)	103	3 (2.9%)	0.000693	0.00272	Wikipathways
Proximal tubule bicarbonate reclamation - Homo sapiens (human	23	2 (8.7%)	0.000694	0.00272	KEGG
Parkinson disease - Homo sapiens (human)	249	4 (1.6%)	0.000773	0.00272	KEGG
Respiratory electron transport, ATP synthesis by chemiosmotic cc	113	3 (2.7%)	0.000908	0.00272	Reactome
Levomethadyl Acetate Action Action Pathway	29	2 (6.9%)	0.00111	0.00272	SMPDB
Levallorphan Action Pathway	29	2 (6.9%)	0.00111	0.00272	SMPDB
Dimethylthiambutene Action Pathway	29	2 (6.9%)	0.00111	0.00272	SMPDB
Ethylmorphine Action Pathway	29	2 (6.9%)	0.00111	0.00272	SMPDB
Pentazocine Action Pathway	29	2 (6.9%)	0.00111	0.00272	SMPDB
Naltrexone Action Pathway	29	2 (6.9%)	0.00111	0.00272	SMPDB
Buprenorphine Action Pathway	29	2 (6.9%)	0.00111	0.00272	SMPDB
Alvimopan Action Pathway	29	2 (6.9%)	0.00111	0.00272	SMPDB
Naloxone Action Pathway	29	2 (6.9%)	0.00111	0.00272	SMPDB
Dihydromorphine Action Pathway	29	2 (6.9%)	0.00111	0.00272	SMPDB
Ketobemidone Action Pathway	29	2 (6.9%)	0.00111	0.00272	SMPDB
Levorphanol Action Pathway	29	2 (6.9%)	0.00111	0.00272	SMPDB
Propoxyphene Action Pathway	29	2 (6.9%)	0.00111	0.00272	SMPDB
Tramadol Action Action Pathway	29	2 (6.9%)	0.00111	0.00272	SMPDB
Diphenoxylate Action Pathway	29	2 (6.9%)	0.00111	0.00272	SMPDB
Anileridine Action Pathway	29	2 (6.9%)	0.00111	0.00272	SMPDB
Oxycodone Action Pathway	29	2 (6.9%)	0.00111	0.00272	SMPDB
Alfentanil Action Pathway	29	2 (6.9%)	0.00111	0.00272	SMPDB
Oxymorphone Action Pathway	29	2 (6.9%)	0.00111	0.00272	SMPDB
Hydrocodone Action Pathway	29	2 (6.9%)	0.00111	0.00272	SMPDB
Hydromorphone Action Pathway	29	2 (6.9%)	0.00111	0.00272	SMPDB
Sufentanil Action Pathway	29	2 (6.9%)	0.00111	0.00272	SMPDB
Remifentanil Action Pathway	29	2 (6.9%)	0.00111	0.00272	SMPDB
Fentanyl Action Pathway	29	2 (6.9%)	0.00111	0.00272	SMPDB
Carfentanil Action Pathway	29	2 (6.9%)	0.00111	0.00272	SMPDB
3-Methylthiofentanyl Action Pathway	29	2 (6.9%)	0.00111	0.00272	SMPDB
Methadyl Acetate Action Pathway	29	2 (6.9%)	0.00111	0.00272	SMPDB
Dezocine Action Pathway	29	2 (6.9%)	0.00111	0.00272	SMPDB
Mepivacaine Action Pathway	29	2 (6.9%)	0.00111	0.00272	SMPDB
Chloroprocaine Action Pathway	29	2 (6.9%)	0.00111	0.00272	SMPDB
Dibucaine Action Pathway	29	2 (6.9%)	0.00111	0.00272	SMPDB
Levobupivacaine Action Pathway	29	2 (6.9%)	0.00111	0.00272	SMPDB
Benzocaine Action Pathway	29	2 (6.9%)	0.00111	0.00272	SMPDB
Bupivacaine Action Pathway	29	2 (6.9%)	0.00111	0.00272	SMPDB
Oxybuprocaine Action Pathway	29	2 (6.9%)	0.00111	0.00272	SMPDB
Prilocaine Action Pathway	29	2 (6.9%)	0.00111	0.00272	SMPDB

Procaine Action Pathway	29	2 (6.9%)	0.00111	0.00272	**SMPDB**
Proparacaine Action Pathway	29	2 (6.9%)	0.00111	0.00272	**SMPDB**
Ropivacaine Action Pathway	29	2 (6.9%)	0.00111	0.00272	**SMPDB**
Cocaine Action Pathway	29	2 (6.9%)	0.00111	0.00272	**SMPDB**
Escitalopram Action Pathway	29	2 (6.9%)	0.00111	0.00272	**SMPDB**
Nalbuphine Action Pathway	30	2 (6.7%)	0.00118	0.00272	**SMPDB**
Desipramine Action Pathway	30	2 (6.7%)	0.00118	0.00272	**SMPDB**
Mitophagy	30	2 (6.7%)	0.00118	0.00272	**Reactome**
Lidocaine (Local Anaesthetic) Action Pathway	31	2 (6.5%)	0.00127	0.00272	**SMPDB**
Heroin Action Pathway	32	2 (6.2%)	0.00135	0.00272	**SMPDB**
Codeine Action Pathway	32	2 (6.2%)	0.00135	0.00272	**SMPDB**
Spironolactone Action Pathway	33	2 (6.1%)	0.00143	0.00272	**SMPDB**
Eplerenone Action Pathway	33	2 (6.1%)	0.00143	0.00272	**SMPDB**
Triamterene Action Pathway	33	2 (6.1%)	0.00143	0.00272	**SMPDB**
Amiloride Action Pathway	33	2 (6.1%)	0.00143	0.00272	**SMPDB**
Imipramine Action Pathway	33	2 (6.1%)	0.00143	0.00272	**SMPDB**
Torsemide Action Pathway	33	2 (6.1%)	0.00143	0.00272	**SMPDB**
Iminoglycinuria	33	2 (6.1%)	0.00143	0.00272	**SMPDB**
Lysinuric Protein Intolerance	33	2 (6.1%)	0.00143	0.00272	**SMPDB**
Blue diaper syndrome	33	2 (6.1%)	0.00143	0.00272	**SMPDB**
Lysinuric protein intolerance (LPI)	33	2 (6.1%)	0.00143	0.00272	**SMPDB**
Cystinuria	33	2 (6.1%)	0.00143	0.00272	**SMPDB**
Hartnup Disorder	33	2 (6.1%)	0.00143	0.00272	**SMPDB**
Glucose Transporter Defect (SGLT2)	33	2 (6.1%)	0.00143	0.00272	**SMPDB**
Kidney Function	33	2 (6.1%)	0.00143	0.00272	**SMPDB**
Glucose Transporter Defect (SGLT2)	33	2 (6.1%)	0.00143	0.00272	**SMPDB**
Quinethazone Action Pathway	33	2 (6.1%)	0.00143	0.00272	**SMPDB**
Bendroflumethiazide Action Pathway	33	2 (6.1%)	0.00143	0.00272	**SMPDB**
Chlorthalidone Action Pathway	33	2 (6.1%)	0.00143	0.00272	**SMPDB**
Trichlormethiazide Action Pathway	33	2 (6.1%)	0.00143	0.00272	**SMPDB**
Indapamide Action Pathway	33	2 (6.1%)	0.00143	0.00272	**SMPDB**
Metolazone Action Pathway	33	2 (6.1%)	0.00143	0.00272	**SMPDB**
Hydrochlorothiazide Action Pathway	33	2 (6.1%)	0.00143	0.00272	**SMPDB**
Cyclothiazide Action Pathway	33	2 (6.1%)	0.00143	0.00272	**SMPDB**
Hydroflumethiazide Action Pathway	33	2 (6.1%)	0.00143	0.00272	**SMPDB**
Bumetanide Action Pathway	33	2 (6.1%)	0.00143	0.00272	**SMPDB**
Ethacrynic Acid Action Pathway	33	2 (6.1%)	0.00143	0.00272	**SMPDB**
Furosemide Action Pathway	33	2 (6.1%)	0.00143	0.00272	**SMPDB**
Polythiazide Action Pathway	33	2 (6.1%)	0.00143	0.00272	**SMPDB**
Methyclothiazide Action Pathway	33	2 (6.1%)	0.00143	0.00272	**SMPDB**
Chlorothiazide Action Pathway	33	2 (6.1%)	0.00143	0.00272	**SMPDB**
Oxidative phosphorylation - Homo sapiens (human)	133	3 (2.3%)	0.00145	0.00274	**KEGG**
Fluoxetine Action Pathway	34	2 (5.9%)	0.00152	0.00282	**SMPDB**
Mitochondrial CIV Assembly	34	2 (5.9%)	0.00152	0.00282	**Wikipathways**
Nicotine Action Pathway	36	2 (5.6%)	0.00171	0.00309	**SMPDB**
Methadone Action Pathway	36	2 (5.6%)	0.00171	0.00309	**SMPDB**
Citalopram Action Pathway	36	2 (5.6%)	0.00171	0.00309	**SMPDB**
Aldosterone-regulated sodium reabsorption - Homo sapiens (hum	37	2 (5.4%)	0.0018	0.00324	**KEGG**
Non-alcoholic fatty liver disease - Homo sapiens (human)	150	3 (2.0%)	0.00205	0.00366	**KEGG**
Nonalcoholic fatty liver disease	155	3 (1.9%)	0.00225	0.00399	**Wikipathways**
Fat digestion and absorption - Homo sapiens (human)	43	2 (4.7%)	0.00243	0.00427	**KEGG**
Ion homeostasis	44	2 (4.5%)	0.00254	0.0044	**Reactome**
Morphine Action Pathway	44	2 (4.5%)	0.00254	0.0044	**SMPDB**
The citric acid (TCA) cycle and respiratory electron transport	162	3 (1.9%)	0.00255	0.0044	**Reactome**
Retinoid metabolism and transport	45	2 (4.4%)	0.00265	0.00454	**Reactome**
Ion channel transport	165	3 (1.8%)	0.00269	0.00456	**Reactome**
Ion transport by P-type ATPases	47	2 (4.3%)	0.00289	0.00487	**Reactome**
Metabolism of fat-soluble vitamins	49	2 (4.1%)	0.00314	0.00524	**Reactome**
Metabolism of proteins	1935	9 (0.5%)	0.00315	0.00524	**Reactome**
Metabolism of vitamins and cofactors	180	3 (1.7%)	0.00344	0.00568	**Reactome**
Endocrine and other factor-regulated calcium reabsorption - Hom	53	2 (3.8%)	0.00366	0.00593	**KEGG**
Proximal tubule transport	57	2 (3.5%)	0.00423	0.00593	**Wikipathways**
Iron uptake and transport	57	2 (3.5%)	0.00423	0.00593	**Reactome**
Selective autophagy	59	2 (3.4%)	0.00452	0.00593	**Reactome**
Diabetic cardiomyopathy - Homo sapiens (human)	203	3 (1.5%)	0.00482	0.00593	**KEGG**
Bopindolol Action Pathway	61	2 (3.3%)	0.00483	0.00593	**SMPDB**
Timolol Action Pathway	61	2 (3.3%)	0.00483	0.00593	**SMPDB**
Carteolol Action Pathway	61	2 (3.3%)	0.00483	0.00593	**SMPDB**
Bevantolol Action Pathway	61	2 (3.3%)	0.00483	0.00593	**SMPDB**
Practolol Action Pathway	61	2 (3.3%)	0.00483	0.00593	**SMPDB**
Dobutamine Action Pathway	61	2 (3.3%)	0.00483	0.00593	**SMPDB**
Isoprenaline Action Pathway	61	2 (3.3%)	0.00483	0.00593	**SMPDB**
Arbutamine Action Pathway	61	2 (3.3%)	0.00483	0.00593	**SMPDB**
Levobunolol Action Pathway	61	2 (3.3%)	0.00483	0.00593	**SMPDB**
Metipranolol Action Pathway	61	2 (3.3%)	0.00483	0.00593	**SMPDB**
Sotalol Action Pathway	61	2 (3.3%)	0.00483	0.00593	**SMPDB**
Epinephrine Action Pathway	61	2 (3.3%)	0.00483	0.00593	**SMPDB**
Betaxolol Action Pathway	61	2 (3.3%)	0.00483	0.00593	**SMPDB**
Atenolol Action Pathway	61	2 (3.3%)	0.00483	0.00593	**SMPDB**
Alprenolol Action Pathway	61	2 (3.3%)	0.00483	0.00593	**SMPDB**
Acebutolol Action Pathway	61	2 (3.3%)	0.00483	0.00593	**SMPDB**
Propranolol Action Pathway	61	2 (3.3%)	0.00483	0.00593	**SMPDB**
Pindolol Action Pathway	61	2 (3.3%)	0.00483	0.00593	**SMPDB**
Penbutolol Action Pathway	61	2 (3.3%)	0.00483	0.00593	**SMPDB**
Oxprenolol Action Pathway	61	2 (3.3%)	0.00483	0.00593	**SMPDB**
Metoprolol Action Pathway	61	2 (3.3%)	0.00483	0.00593	**SMPDB**
Esmolol Action Pathway	61	2 (3.3%)	0.00483	0.00593	**SMPDB**
Bisoprolol Action Pathway	61	2 (3.3%)	0.00483	0.00593	**SMPDB**
Bupranolol Action Pathway	61	2 (3.3%)	0.00483	0.00593	**SMPDB**
Nebivolol Action Pathway	61	2 (3.3%)	0.00483	0.00593	**SMPDB**
Nadolol Action Pathway	61	2 (3.3%)	0.00483	0.00593	**SMPDB**
Muscle/Heart Contraction	61	2 (3.3%)	0.00483	0.00593	**SMPDB**
Diltiazem Action Pathway	61	2 (3.3%)	0.00483	0.00593	**SMPDB**

Amlodipine Action Pathway	61	2 (3.3%)	0.00483	0.00593	**SMPDB**
Verapamil Action Pathway	61	2 (3.3%)	0.00483	0.00593	**SMPDB**
Nitrendipine Action Pathway	61	2 (3.3%)	0.00483	0.00593	**SMPDB**
Nisoldipine Action Pathway	61	2 (3.3%)	0.00483	0.00593	**SMPDB**
Nimodipine Action Pathway	61	2 (3.3%)	0.00483	0.00593	**SMPDB**
Isradipine Action Pathway	61	2 (3.3%)	0.00483	0.00593	**SMPDB**
Nifedipine Action Pathway	61	2 (3.3%)	0.00483	0.00593	**SMPDB**
Felodipine Action Pathway	61	2 (3.3%)	0.00483	0.00593	**SMPDB**
Quinidine Action Pathway	61	2 (3.3%)	0.00483	0.00593	**SMPDB**
Procainamide (Antiarrhythmic) Action Pathway	61	2 (3.3%)	0.00483	0.00593	**SMPDB**
Disopyramide Action Pathway	61	2 (3.3%)	0.00483	0.00593	**SMPDB**
Fosphenytoin (Antiarrhythmic) Action Pathway	61	2 (3.3%)	0.00483	0.00593	**SMPDB**
Mexiletine Action Pathway	61	2 (3.3%)	0.00483	0.00593	**SMPDB**
Tocainide Action Pathway	61	2 (3.3%)	0.00483	0.00593	**SMPDB**
Flecainide Action Pathway	61	2 (3.3%)	0.00483	0.00593	**SMPDB**
Amiodarone Action Pathway	61	2 (3.3%)	0.00483	0.00593	**SMPDB**
Ibutilide Action Pathway	61	2 (3.3%)	0.00483	0.00593	**SMPDB**
Carvedilol Action Pathway	62	2 (3.2%)	0.00498	0.00606	**SMPDB**
Labetalol Action Pathway	62	2 (3.2%)	0.00498	0.00606	**SMPDB**
Lidocaine (Antiarrhythmic) Action Pathway	63	2 (3.2%)	0.00514	0.00622	**SMPDB**
Mitophagy - animal - Homo sapiens (human)	68	2 (2.9%)	0.00596	0.00719	**KEGG**
Thermogenesis - Homo sapiens (human)	232	3 (1.3%)	0.00698	0.00836	**KEGG**
Thyroid hormone synthesis - Homo sapiens (human)	75	2 (2.7%)	0.00721	0.0086	**KEGG**
TP53 Regulates Metabolic Genes	76	2 (2.6%)	0.0074	0.00878	**Reactome**
Pathways of neurodegeneration - multiple diseases - Homo sapie	475	4 (0.8%)	0.00801	0.00946	**KEGG**
Potential therapeutics for SARS	80	2 (2.5%)	0.00817	0.00961	**Reactome**
Phenytoin (Antiarrhythmic) Action Pathway	82	2 (2.4%)	0.00857	0.01	**SMPDB**
Insulin secretion - Homo sapiens (human)	86	2 (2.3%)	0.00939	0.0109	**KEGG**

**DOWNGULATED pathways**	**set size**	**candidates contained**	**p value**	**q value**	**Pathways database**
PAR1-mediated thrombin signaling events	43	7 (16.3%)	0.000196	0.228	**PID**
Phospholipase D signaling pathway - Homo sapiens (human)	148	12 (8.2%)	0.00105	0.457	**KEGG**
Bacterial invasion of epithelial cells - Homo sapiens (human)	77	8 (10.4%)	0.00156	0.457	**KEGG**
GnRH secretion - Homo sapiens (human)	64	7 (10.9%)	0.00226	0.457	**KEGG**
Spinocerebellar ataxia - Homo sapiens (human)	143	11 (7.7%)	0.00268	0.457	**KEGG**
PKC-gamma calcium signaling pathway in ataxia	22	4 (18.2%)	0.0032	0.457	**Wikipathways**
superpathway of inositol phosphate compounds	70	7 (10.0%)	0.00377	0.457	**HumanCyc**
IL1 and megakaryocytes in obesity	24	4 (16.7%)	0.00444	0.457	**Wikipathways**
pyrimidine deoxyribonucleosides degradation	4	2 (50.0%)	0.00471	0.457	**HumanCyc**
Kisspeptin-kisspeptin receptor system in the ovary	39	5 (12.8%)	0.00481	0.457	**Wikipathways**
Shigellosis - Homo sapiens (human)	246	15 (6.1%)	0.00482	0.457	**KEGG**
NOTCH3 Activation and Transmission of Signal to the Nucleus	25	4 (16.0%)	0.00517	0.457	**Reactome**
fosb gene expression and drug abuse	5	2 (40.0%)	0.0077	0.457	**BioCarta**
IL8- and CXCR1-mediated signaling events	28	4 (14.3%)	0.00782	0.457	**PID**
GPR40 Pathway	15	3 (20.0%)	0.00816	0.457	**Wikipathways**
Chagas disease - Homo sapiens (human)	102	8 (7.8%)	0.0088	0.457	**KEGG**
Synthesis of IP3 and IP4 in the cytosol	29	4 (13.8%)	0.00887	0.457	**Reactome**
mRNA decay by 3, to 5, exoribonuclease	16	3 (18.8%)	0.00984	0.457	**Reactome**
Cell-extracellular matrix interactions	16	3 (18.8%)	0.00984	0.457	**Reactome**

Supplemental Table 6. Selected differentially regulated pathways in the ductal cluster #4 of non-pregnant NSG-LIRKO mice compared to non-pregnant NSG-Lox mice (Insulin Resistant model).

List of selected pathways analyzed by ConsensusPathDB.
The p-value is calculated according to the hypergeometric test based on the number of physical entities present in both the predefined set and user-specified list of physical entities. Reference is cited in Methods.

UPREGULATED pathways	set size	candidates contained	p value	q value	Pathways database
Cytoplasmic Ribosomal Proteins	88	3 (3.4%)	7.41E-05	0.000513	**Wikipathways**
Peptide chain elongation	100	3 (3.0%)	0.000108	0.000513	**Reactome**
Eukaryotic Translation Termination	103	3 (2.9%)	0.000118	0.000513	**Reactome**
Selenocysteine synthesis	103	3 (2.9%)	0.000118	0.000513	**Reactome**
Nonsense Mediated Decay (NMD) independent of the Exon Junction Complex (EJC)	105	3 (2.9%)	0.000125	0.000513	**Reactome**
Eukaryotic Translation Elongation	105	3 (2.9%)	0.000125	0.000513	**Reactome**
Response of EIF2AK4 (GCN2) to amino acid deficiency	111	3 (2.7%)	0.000148	0.000513	**Reactome**
Formation of a pool of free 40S subunits	113	3 (2.7%)	0.000156	0.000513	**Reactome**
Nonsense Mediated Decay (NMD) enhanced by the Exon Junction Complex (EJC)	117	3 (2.6%)	0.000173	0.000513	**Reactome**
Nonsense-Mediated Decay (NMD)	117	3 (2.6%)	0.000173	0.000513	**Reactome**
L13a-mediated translational silencing of Ceruloplasmin expression	123	3 (2.4%)	0.0002	0.000513	**Reactome**
SRP-dependent cotranslational protein targeting to membrane	123	3 (2.4%)	0.0002	0.000513	**Reactome**
GTP hydrolysis and joining of the 60S ribosomal subunit	124	3 (2.4%)	0.000205	0.000513	**Reactome**
Selenoamino acid metabolism	128	3 (2.3%)	0.000225	0.000513	**Reactome**
Cap-dependent Translation Initiation	131	3 (2.3%)	0.000241	0.000513	**Reactome**
Eukaryotic Translation Initiation	131	3 (2.3%)	0.000241	0.000513	**Reactome**
Cellular response to starvation	145	3 (2.1%)	0.000325	0.000651	**Reactome**
Ribosome - Homo sapiens (human)	158	3 (1.9%)	0.000419	0.000791	**KEGG**
Metabolism	1954	7 (0.4%)	0.00104	0.00185	**Reactome**
Coronavirus disease - COVID-19 - Homo sapiens (human)	232	3 (1.3%)	0.00126	0.00215	**KEGG**
Metabolism of RNA	584	4 (0.7%)	0.00183	0.00297	**Reactome**
Translation	307	3 (1.0%)	0.00285	0.00441	**Reactome**
Metabolism of amino acids and derivatives	339	3 (0.9%)	0.00377	0.00558	**Reactome**

DOWNGULATED pathways	set size	candidates contained	p value	q value	Pathways database
Mitochondrial translation initiation	88	6 (6.8%)	3.62E-05	0.00463	**Reactome**
Mitochondrial translation	94	6 (6.4%)	5.26E-05	0.00463	**Reactome**
Translation	307	10 (3.3%)	6.09E-05	0.00463	**Reactome**
TNFalpha	234	8 (3.4%)	0.000248	0.0142	**NetPath**
Mitochondrial translation termination	88	5 (5.7%)	0.000392	0.0149	**Reactome**
Mitochondrial translation elongation	88	5 (5.7%)	0.000392	0.0149	**Reactome**
Insertion of tail-anchored proteins into the endoplasmic reticulum membrane	22	3 (13.6%)	0.000479	0.0156	**Reactome**
Glycerophospholipid catabolism	7	2 (28.6%)	0.00102	0.0289	**Reactome**
SEMA3A-Plexin repulsion signaling by inhibiting Integrin adhesion	14	2 (14.3%)	0.00426	0.108	**Reactome**
TGF-beta super family signaling pathway canonical	115	4 (3.5%)	0.00893	0.204	**INOH**

Supplemental Table 7. Selected differentially regulated pathways in the ductal cluster #4 of non-pregnant NSG-LIRKO mice compared to non-pregnant NSG-Lox mice (Combined model).

List of selected pathways analyzed by ConsensusPathDB.
The p-value is calculated according to the hypergeometric test based on the number of physical entities present in both the predefined set and user-specified list of physical entities. Reference is cited in Methods.

UPREGULATED pathways	set size	candidates contained	p value	q value	Pathways database
Pancreatic secretion - Homo sapiens (human)	102	4 (3.9%)	3.83E-07	5.75E-06	**KEGG**
Digestion of dietary lipid	7	2 (28.6%)	8.36E-06	6.27E-05	**Reactome**
Protein digestion and absorption - Homo sapiens (human)	103	3 (2.9%)	3.56E-05	0.000178	**KEGG**
Digestion	22	2 (9.1%)	9.15E-05	0.000343	**Reactome**
Digestion and absorption	26	2 (7.7%)	0.000129	0.000386	**Reactome**
Metabolism of vitamins and cofactors	180	3 (1.7%)	0.000187	0.000468	**Reactome**
Type II interferon signaling (IFNG)	37	2 (5.4%)	0.000263	0.000563	**Wikipathways**
Fat digestion and absorption - Homo sapiens (human)	43	2 (4.7%)	0.000355	0.000649	**KEGG**
Retinoid metabolism and transport	45	2 (4.4%)	0.000389	0.000649	**Reactome**
Metabolism of fat-soluble vitamins	49	2 (4.1%)	0.000462	0.000692	**Reactome**
Salivary secretion - Homo sapiens (human)	93	2 (2.2%)	0.00165	0.00226	**KEGG**
Visual phototransduction	103	2 (1.9%)	0.00202	0.00253	**Reactome**

DOWNGULATED pathways	set size	candidates contained	p value	q value	Pathways database
Cori Cycle	16	3 (18.8%)	0.00012	0.0283	**Wikipathways**
Diabetic cardiomyopathy - Homo sapiens (human)	203	7 (3.4%)	0.000255	0.0283	**KEGG**
Peptide chain elongation	100	5 (5.0%)	0.000379	0.0283	**Reactome**
Eukaryotic Translation Elongation	105	5 (4.8%)	0.000475	0.0283	**Reactome**
Translation	307	8 (2.6%)	0.000606	0.0283	**Reactome**
Formation of a pool of free 40S subunits	113	5 (4.4%)	0.000664	0.0283	**Reactome**
Glycolysis in senescence	7	2 (28.6%)	0.000776	0.0283	**Wikipathways**
SRP-dependent cotranslational protein targeting to membrane	123	5 (4.1%)	0.000974	0.0283	**Reactome**
L13a-mediated translational silencing of Ceruloplasmin expression	123	5 (4.1%)	0.000974	0.0283	**Reactome**
GTP hydrolysis and joining of the 60S ribosomal subunit	124	5 (4.0%)	0.00101	0.0283	**Reactome**
Cap-dependent Translation Initiation	131	5 (3.8%)	0.00129	0.0296	**Reactome**
Eukaryotic Translation Initiation	131	5 (3.8%)	0.00129	0.0296	**Reactome**
VEGFA-VEGFR2 Signaling Pathway	433	9 (2.1%)	0.00138	0.0296	**Wikipathways**
Insulin secretion - Homo sapiens (human)	86	4 (4.7%)	0.00196	0.0392	**KEGG**
Cytoplasmic Ribosomal Proteins	88	4 (4.5%)	0.00213	0.0398	**Wikipathways**
PAR1-mediated thrombin signaling events	43	3 (7.0%)	0.00235	0.0411	**PID**
Neurodegeneration with brain iron accumulation (NBIA) subtypes pathway	44	3 (6.8%)	0.00251	0.0413	**Wikipathways**
Ribosome - Homo sapiens (human)	158	5 (3.2%)	0.00293	0.0456	**KEGG**
Inflammatory mediator regulation of TRP channels - Homo sapiens (human)	98	4 (4.1%)	0.00315	0.0464	**KEGG**
Eukaryotic Translation Termination	103	4 (3.9%)	0.00377	0.0487	**Reactome**
Selenocysteine synthesis	103	4 (3.9%)	0.00377	0.0487	**Reactome**
Synaptic Vesicle Pathway	51	3 (5.9%)	0.00383	0.0487	**Wikipathways**
Nonsense Mediated Decay (NMD) independent of the Exon Junction Complex	105	4 (3.8%)	0.00404	0.0491	**Reactome**
control of skeletal myogenesis by hdac and calcium/calmodulin-dependent kin	16	2 (12.5%)	0.00428	0.0499	**BioCarta**
HIF-1 signaling pathway - Homo sapiens (human)	109	4 (3.7%)	0.00461	0.051	**KEGG**
endocytotic role of ndk phosphins and dynamin	17	2 (11.8%)	0.00483	0.051	**BioCarta**
Response of EIF2AK4 (GCN2) to amino acid deficiency	111	4 (3.6%)	0.00492	0.051	**Reactome**
Complex I biogenesis	57	3 (5.3%)	0.00523	0.0523	**Reactome**
Formation of the ternary complex, and subsequently, the 43S complex	59	3 (5.1%)	0.00576	0.0535	**Reactome**
Nonsense Mediated Decay (NMD) enhanced by the Exon Junction Complex (I	117	4 (3.4%)	0.00592	0.0535	**Reactome**
Nonsense-Mediated Decay (NMD)	117	4 (3.4%)	0.00592	0.0535	**Reactome**
Metabolism	1954	21 (1.1%)	0.00669	0.0586	**Reactome**
regulation of pgc-1a	21	2 (9.5%)	0.00734	0.0589	**BioCarta**
pkc-catalyzed phosphorylation of inhibitory phosphoprotein of myosin phospha	21	2 (9.5%)	0.00734	0.0589	**BioCarta**
Ribosomal scanning and start codon recognition	66	3 (4.5%)	0.00787	0.0589	**Reactome**
Translation initiation complex formation	66	3 (4.5%)	0.00787	0.0589	**Reactome**
Chaperone Mediated Autophagy	22	2 (9.1%)	0.00804	0.0589	**Reactome**
Selenoamino acid metabolism	128	4 (3.1%)	0.00809	0.0589	**Reactome**
Activation of the mRNA upon binding of the cap-binding complex and eIFs, an	67	3 (4.5%)	0.0082	0.0589	**Reactome**
Sphingolipid Metabolism (general overview)	23	2 (8.7%)	0.00877	0.0614	**Wikipathways**
Ephrin B reverse signaling	24	2 (8.3%)	0.00953	0.0635	**PID**
Sphingolipid Metabolism (integrated pathway)	24	2 (8.3%)	0.00953	0.0635	**Wikipathways**

Supplemental Table 8. Selected differentially regulated pathways in type 2 diabetic human beta cells compared to non-diabetic cases (Public Available dataset, GSE81608)
List of selected pathways analyzed by ConsensusPathDB.
The p-value is calculated according to the hypergeometric test based on the number of physical entities present in both the predefined set and user-specified list of physical entities. Reference is cited in Methods.

UPREGULATED pathways	p value	set size	candidates	q value	Pathways database
Translation	1.59E-13	310	108 (35.2%)	3.50E-10	Reactome
Metabolism of proteins	1.08E-12	2008	471 (23.6%)	1.19E-09	Reactome
Metabolism of RNA	1.07E-11	586	169 (28.9%)	7.85E-09	Reactome
SRP-dependent cotranslational protein targeting to membrane	5.72E-11	124	53 (43.1%)	3.14E-08	Reactome
Gene expression (Transcription)	1.57E-10	1373	331 (24.3%)	5.77E-08	Reactome
L13a-mediated translational silencing of Ceruloplasmin expression	2.02E-10	125	52 (42.3%)	5.77E-08	Reactome
Nonsense Mediated Decay (NMD) enhanced by the Exon Junction Complex (EJC)	2.84E-10	118	50 (42.7%)	5.77E-08	Reactome
Nonsense-Mediated Decay (NMD)	2.84E-10	118	50 (42.7%)	5.77E-08	Reactome
Cap-dependent Translation Initiation	2.85E-10	133	54 (41.2%)	5.77E-08	Reactome
Eukaryotic Translation Initiation	2.85E-10	133	54 (41.2%)	5.77E-08	Reactome
GTP hydrolysis and joining of the 60S ribosomal subunit	2.89E-10	126	52 (41.9%)	5.77E-08	Reactome
Formation of a pool of free 40S subunits	8.24E-10	115	48 (42.5%)	1.51E-07	Reactome
Peptide chain elongation	1.08E-09	101	44 (44.0%)	1.82E-07	Reactome
RNA Polymerase II Transcription	1.53E-09	1236	298 (24.3%)	2.39E-07	Reactome
Nonsense Mediated Decay (NMD) independent of the Exon Junction Complex (EJC)	1.96E-09	106	45 (42.9%)	2.87E-07	Reactome
Eukaryotic Translation Elongation	6.86E-09	106	44 (41.9%)	9.42E-07	Reactome
Eukaryotic Translation Termination	1.16E-08	104	43 (41.7%)	1.42E-06	Reactome
Selenocysteine synthesis	1.16E-08	104	43 (41.7%)	1.42E-06	Reactome
Cytoplasmic Ribosomal Proteins	2.73E-08	88	38 (43.2%)	3.16E-06	Wikipathways
Selenoamino acid metabolism	4.51E-08	130	49 (38.0%)	4.95E-06	Reactome
Protein processing in endoplasmic reticulum - Homo sapiens (human)	6.98E-08	165	58 (35.2%)	7.30E-06	KEGG
Generic Transcription Pathway	2.13E-07	1107	260 (23.7%)	2.13E-05	Reactome
RNA transport - Homo sapiens (human)	2.84E-07	171	58 (33.9%)	2.71E-05	KEGG
Class I MHC mediated antigen processing & presentation	3.41E-07	324	94 (29.1%)	3.12E-05	Reactome
Respiratory electron transport, ATP synthesis by chemiosmotic coupling, and heat production by uncoupling p	5.54E-07	123	45 (36.6%)	4.87E-05	Reactome
ER to Golgi Anterograde Transport	1.02E-06	137	48 (35.0%)	8.64E-05	Reactome
Antigen Presentation: Folding, assembly and peptide loading of class I MHC	1.45E-06	24	15 (62.5%)	0.000118	Reactome
Neddylation	1.78E-06	235	71 (30.3%)	0.000139	Reactome
COPII-mediated vesicle transport	2.32E-06	69	29 (42.0%)	0.000176	Reactome
Ribosome - Homo sapiens (human)	2.61E-06	153	51 (33.3%)	0.000191	KEGG
Asparagine N-linked glycosylation	3.09E-06	286	82 (28.8%)	0.000219	Reactome
Respiratory electron transport	4.04E-06	100	37 (37.0%)	0.000277	Reactome
Membrane Trafficking	4.68E-06	582	146 (25.1%)	0.000312	Reactome
Oxidative phosphorylation - Homo sapiens (human)	6.37E-06	133	45 (33.8%)	0.000412	KEGG
Post-translational protein modification	7.30E-06	1383	306 (22.2%)	0.000458	Reactome
The citric acid (TCA) cycle and respiratory electron transport	1.21E-05	173	54 (31.2%)	0.00074	Reactome
Activation of the mRNA upon binding of the cap-binding complex and eIFs, and subsequent binding to 43S	1.31E-05	69	27 (40.3%)	0.000781	Reactome
HSP90 chaperone cycle for steroid hormone receptors (SHR)	1.47E-05	19	12 (63.2%)	0.000848	Reactome
Proteasome Degradation	1.60E-05	64	26 (40.6%)	0.000899	Wikipathways
Neurexins and neuroligins	1.64E-05	57	24 (42.1%)	0.000901	Reactome
Vesicle-mediated transport	1.79E-05	620	151 (24.4%)	0.000959	Reactome
Transport to the Golgi and subsequent modification	2.26E-05	168	52 (31.0%)	0.00118	Reactome
Electron Transport Chain (OXPHOS system in mitochondria)	2.35E-05	103	36 (35.0%)	0.0012	Wikipathways
Translation initiation complex formation	3.04E-05	68	26 (39.4%)	0.00148	Reactome
Ribosomal scanning and start codon recognition	3.04E-05	68	26 (39.4%)	0.00148	Reactome
snRNP Assembly	3.35E-05	52	22 (42.3%)	0.00157	Reactome
Metabolism of non-coding RNA	3.35E-05	52	22 (42.3%)	0.00157	Reactome
Cellular responses to stress	3.68E-05	345	91 (26.5%)	0.00168	Reactome
InlA-mediated entry of Listeria monocytogenes into host cells	3.86E-05	8	7 (87.5%)	0.00169	Reactome
GLI3 is processed to GLI3R by the proteasome	4.01E-05	15	10 (66.7%)	0.00169	Reactome
Degradation of GLI2 by the proteasome	4.01E-05	15	10 (66.7%)	0.00169	Reactome
Degradation of GLI1 by the proteasome	4.01E-05	16	10 (66.7%)	0.00169	Reactome
TP53 Regulates Metabolic Genes	4.31E-05	86	31 (36.0%)	0.00178	Reactome
Cellular responses to external stimuli	5.69E-05	414	105 (25.4%)	0.00232	Reactome
Cellular response to heat stress	5.89E-05	99	34 (34.3%)	0.00235	Reactome
Antigen processing: Ubiquitination & Proteasome degradation	7.30E-05	264	72 (27.4%)	0.00286	Reactome
Parkinson disease - Homo sapiens (human)	9.08E-05	142	44 (31.0%)	0.0035	KEGG
Metabolism of amino acids and derivatives	0.0001	342	88 (26.0%)	0.00386	Reactome
Formation of the ternary complex, and subsequently, the 43S complex	0.0001	61	23 (39.0%)	0.00387	Reactome
Alzheimer disease - Homo sapiens (human)	0.00016	171	50 (29.2%)	0.00587	KEGG
Protein-protein interactions at synapses	0.00018	88	30 (34.1%)	0.00648	Reactome
Downregulation of TGF-beta receptor signaling	0.00018	26	13 (50.0%)	0.00648	Reactome
Ubiquitin-dependent degradation of Cyclin D1	0.00019	7	6 (85.7%)	0.00648	Reactome
Ubiquitin-dependent degradation of Cyclin D	0.00019	7	6 (85.7%)	0.00648	Reactome
Vibrio cholerae infection - Homo sapiens (human)	0.00019	50	20 (40.0%)	0.00648	KEGG
Host Interactions of HIV factors	0.00023	89	30 (33.7%)	0.00761	Reactome
Huntington disease - Homo sapiens (human)	0.00031	193	54 (28.0%)	0.0101	KEGG
Cytosolic tRNA aminoacylation	0.00033	24	12 (50.0%)	0.0105	Reactome
Signaling by TGF-beta Receptor Complex	0.00034	67	24 (35.8%)	0.0108	Reactome
SCF(Skp2)-mediated degradation of p27/p21	0.00035	18	10 (55.6%)	0.0109	Reactome
Insulin processing	0.00041	10	7 (70.0%)	0.0128	Reactome
Spliceosome - Homo sapiens (human)	0.00044	134	40 (29.9%)	0.0134	KEGG
Transcriptional Regulation by TP53	0.00063	374	91 (24.5%)	0.019	Reactome
Hedgehog ,off, state	0.00067	44	17 (39.5%)	0.0199	Reactome
Transport of Mature Transcript to Cytoplasm	0.0007	82	27 (32.9%)	0.0205	Reactome
HSF1-dependent transactivation	0.00072	36	15 (41.7%)	0.0207	Reactome
Ciliary landscape	0.00073	213	57 (26.8%)	0.0207	Wikipathways
Intra-Golgi and retrograde Golgi-to-ER traffic	0.00074	186	51 (27.4%)	0.021	Reactome
COPI-mediated anterograde transport	0.00086	83	27 (32.5%)	0.024	Reactome
Cyclin D associated events in G1	0.00091	44	17 (38.6%)	0.0248	Reactome
G1 Phase	0.00091	44	17 (38.6%)	0.0248	Reactome
Vitamin B6-dependent and responsive disorders	0.00101	4	4 (100.0%)	0.0271	Wikipathways
Cell Cycle	0.00107	564	129 (22.9%)	0.0283	Reactome
M Phase	0.00113	340	83 (24.5%)	0.0295	Reactome
Cell Cycle, Mitotic	0.00115	481	112 (23.3%)	0.0295	Reactome
Processing of Capped Intron-Containing Pre-mRNA	0.00116	240	62 (25.8%)	0.0295	Reactome
Proteasome - Homo sapiens (human)	0.00123	45	17 (37.8%)	0.031	KEGG

Regulation of HSF1-mediated heat shock response	0.00133	81	26 (32.1%)	0.0331	Reactome
Transport of Mature mRNA derived from an Intron-Containing Transcript	0.00138	73	24 (32.9%)	0.034	Reactome
MAP kinase activation	0.00141	65	22 (33.8%)	0.034	Reactome
Interleukin-17 signaling	0.00141	65	22 (33.8%)	0.034	Reactome
Glucose metabolism	0.00151	91	28 (31.1%)	0.0361	Reactome
Downregulation of SMAD2/3:SMAD4 transcriptional activity	0.00166	21	10 (47.6%)	0.0392	Reactome
Glycolysis	0.00174	71	23 (32.9%)	0.0407	Reactome
Iron uptake and transport	0.00177	58	20 (34.5%)	0.0409	Reactome
Regulation of RUNX2 expression and activity	0.00181	28	12 (42.9%)	0.0415	Reactome
Metabolism	0.00187	1972	396 (20.2%)	0.0423	Reactome
antigen processing and presentation	0.00195	12	7 (58.3%)	0.0437	BioCarta
TGF-beta receptor signaling activates SMADs	0.00212	32	13 (40.6%)	0.047	Reactome
Detoxification of Reactive Oxygen Species	0.00236	36	14 (38.9%)	0.0519	Reactome
Negative regulation of MAPK pathway	0.00255	40	15 (37.5%)	0.0554	Reactome
Downregulation of ERBB2 signaling	0.00259	29	12 (41.4%)	0.0559	Reactome
Cilium Assembly	0.00264	187	49 (26.2%)	0.0563	Reactome
Viral carcinogenesis - Homo sapiens (human)	0.00267	201	52 (25.9%)	0.0564	KEGG
Vpu mediated degradation of CD4	0.00274	7	5 (71.4%)	0.0572	Reactome
Amino sugar and nucleotide sugar metabolism - Homo sapiens (human)	0.00276	48	17 (35.4%)	0.0572	KEGG
Translesion Synthesis by POLH	0.00297	19	9 (47.4%)	0.0604	Reactome
Listeria monocytogenes entry into host cells	0.00297	19	9 (47.4%)	0.0604	Reactome
Toll Like Receptor 9 (TLR9) Cascade	0.00308	94	28 (29.8%)	0.062	Reactome
Organelle biogenesis and maintenance	0.00312	240	60 (25.0%)	0.062	Reactome
Insulin receptor recycling	0.00316	26	11 (42.3%)	0.062	Reactome
signaling pathway from g-protein families	0.00316	26	11 (42.3%)	0.062	BioCarta
Signaling by NOTCH1	0.00321	74	23 (31.5%)	0.0623	Reactome
MAP3K8 (TPL2)-dependent MAPK1/3 activation	0.00334	16	8 (50.0%)	0.0633	Reactome
TGF-beta receptor signaling in EMT (epithelial to mesenchymal transition)	0.00334	16	8 (50.0%)	0.0633	Reactome
Oxidative phosphorylation	0.00348	61	20 (32.8%)	0.0633	Wikipathways
Intra-Golgi traffic	0.00348	45	16 (35.6%)	0.0633	Reactome
Signal attenuation	0.00349	10	6 (60.0%)	0.0633	Reactome
Vif-mediated degradation of APOBEC3G	0.00349	10	6 (60.0%)	0.0633	Reactome
SCF-beta-TrCP mediated degradation of Emi1	0.00349	10	6 (60.0%)	0.0633	Reactome
FBXL7 down-regulates AURKA during mitotic entry and in early mitosis	0.00349	10	6 (60.0%)	0.0633	Reactome
DNA Damage Bypass	0.00354	49	17 (34.7%)	0.0634	Reactome
Endosomal/Vacuolar pathway	0.00358	13	7 (53.8%)	0.0634	Reactome
Downregulation of ERBB2:ERBB3 signaling	0.00358	13	7 (53.8%)	0.0634	Reactome
MyD88 cascade initiated on plasma membrane	0.00405	87	26 (29.9%)	0.07	Reactome
Toll Like Receptor 10 (TLR10) Cascade	0.00405	87	26 (29.9%)	0.07	Reactome
Toll Like Receptor 5 (TLR5) Cascade	0.00405	87	26 (29.9%)	0.07	Reactome
Transcriptional activity of SMAD2/SMAD3:SMAD4 heterotrimer	0.00423	38	14 (36.8%)	0.0726	Reactome
Thermogenesis - Homo sapiens (human)	0.00431	229	57 (24.9%)	0.0732	KEGG
Free fatty acid receptors	0.00433	5	4 (80.0%)	0.0732	Reactome
tRNA Aminoacylation	0.00438	42	15 (35.7%)	0.0735	Reactome
SUMOylation of RNA binding proteins	0.00446	46	16 (34.8%)	0.0743	Reactome
TRAF6 mediated induction of NFkB and MAP kinases upon TLR7/8 or 9 activation	0.00479	88	26 (29.5%)	0.0791	Reactome
Trafficking of AMPA receptors	0.00498	31	12 (38.7%)	0.0804	Reactome
Glutamate binding, activation of AMPA receptors and synaptic plasticity	0.00498	31	12 (38.7%)	0.0804	Reactome
Activated NOTCH1 Transmits Signal to the Nucleus	0.00498	32	12 (38.7%)	0.0804	Reactome
Anchoring of the basal body to the plasma membrane	0.00502	97	28 (28.9%)	0.0804	Reactome
E3 ubiquitin ligases ubiquitinate target proteins	0.00541	59	19 (32.2%)	0.0851	Reactome
UCH proteinases	0.0055	102	29 (28.4%)	0.0851	Reactome
Degradation of beta-catenin by the destruction complex	0.00553	39	14 (35.9%)	0.0851	Reactome
Activation of NMDA receptor and postsynaptic events	0.00553	39	14 (35.9%)	0.0851	Reactome
Translesion synthesis by Y family DNA polymerases bypasses lesions on DNA template	0.00553	39	14 (35.9%)	0.0851	Reactome
Complex I biogenesis	0.00554	55	18 (32.7%)	0.0851	Reactome
opposing roles of aif in apoptosis and cell survival	0.00567	3	3 (100.0%)	0.0865	BioCarta
Mitotic Telophase/Cytokinesis	0.00606	14	7 (50.0%)	0.0912	Reactome
Golgi Cisternae Pericentriolar Stack Reorganization	0.00606	14	7 (50.0%)	0.0912	Reactome
Attenuation phase	0.00621	28	11 (39.3%)	0.0927	Reactome
Downregulation of ERBB4 signaling	0.00624	8	5 (62.5%)	0.0927	Reactome
Establishment of Sister Chromatid Cohesion	0.00652	11	6 (54.5%)	0.0961	Reactome
MyD88 dependent cascade initiated on endosome	0.00662	90	26 (28.9%)	0.0963	Reactome
Toll Like Receptor 7/8 (TLR7/8) Cascade	0.00662	90	26 (28.9%)	0.0963	Reactome
Gene and protein expression by JAK-STAT signaling after Interleukin-12 stimulation	0.00699	36	13 (36.1%)	0.101	Wikipathways
Parkin-Ubiquitin Proteasomal System pathway	0.00701	73	22 (30.1%)	0.101	Wikipathways
Regulation of PLK1 Activity at G2/M Transition	0.00822	87	25 (28.7%)	0.117	Reactome
Signaling by TGF-beta family members	0.00842	96	27 (28.1%)	0.118	Reactome
Cyclin E associated events during G1/S transition	0.00842	29	11 (37.9%)	0.118	Reactome
Cyclin A:Cdk2-associated events at S phase entry	0.00842	29	11 (37.9%)	0.118	Reactome
Antigen processing-Cross presentation	0.00886	49	16 (32.7%)	0.123	Reactome
Glycolysis and Gluconeogenesis	0.00903	45	15 (33.3%)	0.125	Wikipathways
Type II diabetes mellitus	0.0096	22	9 (40.9%)	0.13	Wikipathways
Constitutive Signaling by NOTCH1 HD Domain Mutants	0.00964	15	7 (46.7%)	0.13	Reactome
Signaling by NOTCH1 HD Domain Mutants in Cancer	0.00964	15	7 (46.7%)	0.13	Reactome
Regulation of innate immune responses to cytosolic DNA	0.00964	15	7 (46.7%)	0.13	Reactome

**DOWNREGULATED pathways**	**p value**	**set size**	**candidates**	**q value**	**Pathways database**
Respiratory electron transport, ATP synthesis by chemiosmotic coupling, and heat production by uncoupling p	2.23E-11	123	37 (30.1%)	5.75E-08	Reactome
The citric acid (TCA) cycle and respiratory electron transport	1.44E-10	173	44 (25.4%)	1.86E-07	Reactome
Oxidative phosphorylation - Homo sapiens (human)	2.73E-10	133	37 (27.8%)	2.35E-07	KEGG
Metabolism of proteins	5.51E-10	2008	255 (12.8%)	3.56E-07	Reactome
Translation	3.75E-09	310	60 (19.8%)	1.75E-06	Reactome
Electron Transport Chain (OXPHOS system in mitochondria)	4.07E-09	103	30 (29.1%)	1.75E-06	Wikipathways
Respiratory electron transport	8.22E-09	100	29 (29.0%)	3.04E-06	Reactome
Alzheimer disease - Homo sapiens (human)	4.23E-08	171	39 (22.8%)	1.37E-05	KEGG
Parkinson disease - Homo sapiens (human)	8.85E-08	142	34 (23.9%)	2.54E-05	KEGG
Huntington disease - Homo sapiens (human)	4.38E-07	193	40 (20.7%)	0.000113	KEGG
ER to Golgi Anterograde Transport	1.22E-06	137	31 (22.6%)	0.000286	Reactome
Mitochondrial Electron Transport Chain	2.73E-06	20	10 (50.0%)	0.000588	SMPDB
Thermogenesis - Homo sapiens (human)	6.47E-06	229	42 (18.3%)	0.00129	KEGG

Metabolism	1.47E-05	1972	227 (11.6%)	0.00271	Reactome
Oxidative phosphorylation	1.80E-05	61	17 (27.9%)	0.00309	Wikipathways
isoleucine degradation	2.52E-05	12	7 (58.3%)	0.00407	HumanCyc
Asparagine N-linked glycosylation	3.43E-05	286	47 (16.5%)	0.00482	Reactome
Cellular responses to external stimuli	3.57E-05	414	62 (15.1%)	0.00482	Reactome
Insulin receptor recycling	4.74E-05	26	10 (38.5%)	0.00482	Reactome
Lysine Degradation	5.03E-05	13	7 (53.8%)	0.00482	SMPDB
Hyperlysinemia I, Familial	5.03E-05	13	7 (53.8%)	0.00482	SMPDB
2-aminoadipic 2-oxoadipic aciduria	5.03E-05	13	7 (53.8%)	0.00482	SMPDB
Pyridoxine dependency with seizures	5.03E-05	13	7 (53.8%)	0.00482	SMPDB
Saccharopinuria/Hyperlysinemia II	5.03E-05	13	7 (53.8%)	0.00482	SMPDB
Glutaric Aciduria Type I	5.03E-05	13	7 (53.8%)	0.00482	SMPDB
Hyperlysinemia II or Saccharopinuria	5.03E-05	13	7 (53.8%)	0.00482	SMPDB
valine degradation	5.03E-05	13	7 (53.8%)	0.00482	HumanCyc
Non-alcoholic fatty liver disease (NAFLD) - Homo sapiens (human)	5.59E-05	149	29 (19.5%)	0.00502	KEGG
Valine, leucine and isoleucine degradation - Homo sapiens (human)	5.63E-05	48	14 (29.2%)	0.00502	KEGG
Protein processing in endoplasmic reticulum - Homo sapiens (human)	6.36E-05	165	31 (18.8%)	0.00548	KEGG
Complex I biogenesis	7.29E-05	55	15 (27.3%)	0.00608	Reactome
adenosine ribonucleotides *de novo* biosynthesis	8.08E-05	38	12 (31.6%)	0.00644	HumanCyc
Propanoate metabolism	8.22E-05	18	8 (44.4%)	0.00644	EHMN
Vesicle-mediated transport	8.98E-05	620	84 (13.5%)	0.00648	Reactome
Transport to the Golgi and subsequent modification	9.03E-05	168	31 (18.5%)	0.00648	Reactome
Vibrio cholerae infection - Homo sapiens (human)	9.22E-05	50	14 (28.0%)	0.00648	KEGG
Golgi Cisternae Pericentriolar Stack Reorganization	9.28E-05	14	7 (50.0%)	0.00648	Reactome
TLR NFkB	9.98E-05	69	17 (24.6%)	0.00662	INOH
COPII-mediated vesicle transport	9.98E-05	69	17 (24.6%)	0.00662	Reactome
COPI-mediated anterograde transport	0.00012	83	19 (22.9%)	0.00737	Reactome
Nonalcoholic fatty liver disease	0.00012	155	29 (18.7%)	0.00737	Wikipathways
Proteasome Degradation	0.00013	64	16 (25.0%)	0.00792	Wikipathways
IL-1 NFkB	0.00013	64	16 (25.0%)	0.00792	INOH
Membrane Trafficking	0.00014	582	79 (13.6%)	0.00801	Reactome
Post-translational protein modification	0.00015	1383	162 (11.8%)	0.00884	Reactome
Ciliary landscape	0.00017	213	36 (16.9%)	0.00935	Wikipathways
HSF1-dependent transactivation	0.00022	36	11 (30.6%)	0.0122	Reactome
Cellular responses to stress	0.00023	345	51 (14.9%)	0.0124	Reactome
Neddylation	0.00026	235	38 (16.2%)	0.0135	Reactome
Mitochondrial translation initiation	0.00026	89	19 (21.6%)	0.0135	Reactome
Signaling by Insulin receptor	0.0003	75	17 (22.7%)	0.015	Reactome
Free fatty acid receptors	0.00031	5	4 (80.0%)	0.0152	Reactome
tRNA charging	0.00038	38	11 (28.9%)	0.018	HumanCyc
TLR p38	0.00038	63	15 (23.8%)	0.018	INOH
Lysine degradation	0.00043	22	8 (36.4%)	0.0203	INOH
Proteasome - Homo sapiens (human)	0.00048	45	12 (26.7%)	0.0219	KEGG
Iron uptake and transport	0.0005	58	14 (24.1%)	0.0228	Reactome
CD4 T cell receptor signaling	0.00054	130	24 (18.5%)	0.0239	INOH
Hedgehog	0.00056	72	16 (22.2%)	0.0245	INOH
superpathway of purine nucleotide salvage	0.00061	59	14 (23.7%)	0.025	HumanCyc
purine nucleotides *de novo* biosynthesis	0.00061	59	14 (23.7%)	0.025	HumanCyc
Branched-chain amino acid catabolism	0.00061	23	8 (34.8%)	0.025	Reactome
Cristae formation	0.00062	18	7 (38.9%)	0.025	Reactome
Formation of ATP by chemiosmotic coupling	0.00062	18	7 (38.9%)	0.025	Reactome
Mitochondrial translation	0.00063	95	19 (20.2%)	0.025	Reactome
IL-1 p38	0.00064	66	15 (22.7%)	0.0251	INOH
opposing roles of aif in apoptosis and cell survival	0.00073	3	3 (100.0%)	0.0263	BioCarta
Mitochondrial translation termination	0.00075	89	18 (20.5%)	0.0263	Reactome
Mitochondrial translation elongation	0.00075	89	18 (20.5%)	0.0263	Reactome
TNF	0.00076	67	15 (22.4%)	0.0263	INOH
Detoxification of Reactive Oxygen Species	0.00077	36	10 (28.6%)	0.0263	Reactome
Cytosolic tRNA aminoacylation	0.00085	24	8 (33.3%)	0.0263	Reactome
Scavenging by Class F Receptors	0.00085	6	4 (66.7%)	0.0263	Reactome
Amino sugar and nucleotide sugar metabolism - Homo sapiens (human)	0.00089	48	12 (25.0%)	0.0263	KEGG
HSP90 chaperone cycle for steroid hormone receptors (SHR)	0.00091	19	7 (36.8%)	0.0263	Reactome
CD4 T cell receptor signaling-NFkB cascade	0.00094	97	19 (19.6%)	0.0263	INOH
tRNA Aminoacylation	0.00096	42	11 (26.2%)	0.0263	Reactome
3-Methylglutaconic Aciduria Type I	0.00096	30	9 (30.0%)	0.0263	SMPDB
Valine, Leucine and Isoleucine Degradation	0.00096	30	9 (30.0%)	0.0263	SMPDB
2-Methyl-3-Hydroxybutryl CoA Dehydrogenase Deficiency	0.00096	30	9 (30.0%)	0.0263	SMPDB
Isovaleric Aciduria	0.00096	30	9 (30.0%)	0.0263	SMPDB
3-Methylcrotonyl Coa Carboxylase Deficiency Type I	0.00096	30	9 (30.0%)	0.0263	SMPDB
Propionic Acidemia	0.00096	30	9 (30.0%)	0.0263	SMPDB
Maple Syrup Urine Disease	0.00096	30	9 (30.0%)	0.0263	SMPDB
3-Hydroxy-3-Methylglutaryl-CoA Lyase Deficiency	0.00096	30	9 (30.0%)	0.0263	SMPDB
Isobutyryl-coa dehydrogenase deficiency	0.00096	30	9 (30.0%)	0.0263	SMPDB
3-hydroxyisobutyric aciduria	0.00096	30	9 (30.0%)	0.0263	SMPDB
3-hydroxyisobutyric acid dehydrogenase deficiency	0.00096	30	9 (30.0%)	0.0263	SMPDB
Isovaleric acidemia	0.00096	30	9 (30.0%)	0.0263	SMPDB
Methylmalonate Semialdehyde Dehydrogenase Deficiency	0.00096	30	9 (30.0%)	0.0263	SMPDB
Methylmalonic Aciduria	0.00096	30	9 (30.0%)	0.0263	SMPDB
3-Methylglutaconic Aciduria Type IV	0.00096	30	9 (30.0%)	0.0263	SMPDB
3-Methylglutaconic Aciduria Type III	0.00096	30	9 (30.0%)	0.0263	SMPDB
Beta-Ketothiolase Deficiency	0.00096	30	9 (30.0%)	0.0263	SMPDB
Signal attenuation	0.00101	10	5 (50.0%)	0.0274	Reactome
TLR JNK	0.00103	62	14 (22.6%)	0.0274	INOH
IL-1 JNK	0.00103	62	14 (22.6%)	0.0274	INOH
Valine, leucine and isoleucine degradation	0.00109	49	12 (24.5%)	0.0287	EHMN
Transferrin endocytosis and recycling	0.00124	31	9 (29.0%)	0.0324	Reactome
Pentose phosphate pathway (hexose monophosphate shunt)	0.0013	15	6 (40.0%)	0.0337	Reactome
Notch	0.00139	79	16 (20.5%)	0.0357	INOH
DNA Repair	0.00148	320	45 (14.2%)	0.0376	Reactome
Transcriptional Regulation by TP53	0.00153	374	51 (13.7%)	0.0383	Reactome
TP53 Regulates Metabolic Genes	0.00154	86	17 (19.8%)	0.0383	Reactome

Citrate cycle	0.00159	32	9 (28.1%)	0.0392	INOH
Integration of energy metabolism	0.00166	94	18 (19.1%)	0.0394	Reactome
DroToll-like	0.00167	65	14 (21.5%)	0.0394	INOH
MAP kinase activation	0.00167	65	14 (21.5%)	0.0394	Reactome
Interleukin-17 signaling	0.00167	65	14 (21.5%)	0.0394	Reactome
Phenylalanine and Tyrosine Metabolism	0.00172	11	5 (45.5%)	0.0394	SMPDB
Phenylketonuria	0.00172	11	5 (45.5%)	0.0394	SMPDB
Tyrosinemia Type 3 (TYRO3)	0.00172	11	5 (45.5%)	0.0394	SMPDB
Tyrosinemia Type 2 (or Richner-Hanhart syndrome)	0.00172	11	5 (45.5%)	0.0394	SMPDB
Glycolysis and Gluconeogenesis	0.00176	45	11 (24.4%)	0.0396	Wikipathways
Warburg Effect	0.00176	45	11 (24.4%)	0.0396	SMPDB
Ubiquitin-dependent degradation of Cyclin D1	0.00185	7	4 (57.1%)	0.0408	Reactome
Ubiquitin-dependent degradation of Cyclin D	0.00185	7	4 (57.1%)	0.0408	Reactome
E3 ubiquitin ligases ubiquitinate target proteins	0.00195	59	13 (22.0%)	0.0426	Reactome
transcription factor creb and its extracellular signals	0.002	27	8 (29.6%)	0.0434	BioCarta
Cap-dependent Translation Initiation	0.00211	133	22 (17.3%)	0.0452	Reactome
Eukaryotic Translation Initiation	0.00211	133	22 (17.3%)	0.0452	Reactome
TP53 Regulates Transcription of DNA Repair Genes	0.00226	70	14 (20.9%)	0.0471	Reactome
Gluconeogenesis	0.00242	22	7 (31.8%)	0.0471	SMPDB
Glycogenosis, Type IA. Von gierke disease	0.00242	22	7 (31.8%)	0.0471	SMPDB
Glycogenosis, Type IC	0.00242	22	7 (31.8%)	0.0471	SMPDB
Glycogen Storage Disease Type 1A (GSD1A) or Von Gierke Disease	0.00242	22	7 (31.8%)	0.0471	SMPDB
Triosephosphate isomerase	0.00242	22	7 (31.8%)	0.0471	SMPDB
Fructose-1,6-diphosphatase deficiency	0.00242	22	7 (31.8%)	0.0471	SMPDB
Phosphoenolpyruvate carboxykinase deficiency 1 (PEPCK1)	0.00242	22	7 (31.8%)	0.0471	SMPDB
Glycogenosis, Type IB	0.00242	22	7 (31.8%)	0.0471	SMPDB
Metabolism of amino acids and derivatives	0.00245	342	46 (13.8%)	0.0471	Reactome
Organelle biogenesis and maintenance	0.00246	240	35 (14.8%)	0.0471	Reactome
Ub-specific processing proteases	0.0025	220	33 (15.0%)	0.0471	Reactome
Gluconeogenesis	0.00253	35	9 (26.5%)	0.0471	Reactome
Attenuation phase	0.00258	28	8 (28.6%)	0.0471	Reactome
Prefoldin mediated transfer of substrate to CCT/TriC	0.00258	28	8 (28.6%)	0.0471	Reactome
Glycerolipid metabolism - Homo sapiens (human)	0.00267	61	13 (21.3%)	0.0471	KEGG
Processing of DNA double-strand break ends	0.00269	99	18 (18.4%)	0.0471	Reactome
Hypusine synthesis from eIF5A-lysine	0.00273	4	3 (75.0%)	0.0471	Reactome
Resolution of AP sites via the single-nucleotide replacement pathway	0.00273	4	3 (75.0%)	0.0471	Reactome
lysine degradation I (saccharopine pathway)	0.00273	4	3 (75.0%)	0.0471	HumanCyc
Vitamin B6-dependent and responsive disorders	0.00273	4	3 (75.0%)	0.0471	Wikipathways
Dimethyl-branched-chain fatty acid mitochondrial beta-oxidation	0.00273	12	5 (41.7%)	0.0471	EHMN
il 3 signaling pathway	0.00273	12	5 (41.7%)	0.0471	BioCarta
Sialuria or French Type Sialuria	0.00275	17	6 (35.3%)	0.0471	SMPDB
Sialuria or French Type Sialuria	0.00275	17	6 (35.3%)	0.0471	SMPDB
Amino Sugar Metabolism	0.00275	17	6 (35.3%)	0.0471	SMPDB
G(M2)-Gangliosidosis: Variant B, Tay-sachs disease	0.00275	17	6 (35.3%)	0.0471	SMPDB
Tay-Sachs Disease	0.00275	17	6 (35.3%)	0.0471	SMPDB
Salla Disease/Infantile Sialic Acid Storage Disease	0.00275	17	6 (35.3%)	0.0471	SMPDB
Butanoate metabolism	0.00275	17	6 (35.3%)	0.0471	INOH
Metabolism of RNA	0.00301	586	72 (12.4%)	0.0512	Reactome
Homology Directed Repair	0.00312	141	23 (16.5%)	0.0527	Reactome
how progesterone initiates the oocyte maturation	0.00321	23	7 (30.4%)	0.054	BioCarta
Butyrate Metabolism	0.00343	8	4 (50.0%)	0.0564	SMPDB
InlA-mediated entry of Listeria monocytogenes into host cells	0.00343	8	4 (50.0%)	0.0564	Reactome
Mitochondrial Beta-Oxidation of Medium Chain Saturated Fatty Acids	0.00343	8	4 (50.0%)	0.0564	SMPDB
Prolactin signaling pathway - Homo sapiens (human)	0.00346	70	14 (20.0%)	0.0567	KEGG
Synaptic vesicle cycle - Homo sapiens (human)	0.00359	63	13 (20.6%)	0.0584	KEGG
Golgi Associated Vesicle Biogenesis	0.00366	56	12 (21.4%)	0.0588	Reactome
TNFalpha	0.00366	234	34 (14.5%)	0.0588	NetPath
Gene and protein expression by JAK-STAT signaling after Interleukin-12 stimulation	0.00385	36	9 (25.0%)	0.0614	Wikipathways
Toll Like Receptor 9 (TLR9) Cascade	0.00411	94	17 (18.1%)	0.0643	Reactome
Pentose phosphate pathway - Homo sapiens (human)	0.00413	30	8 (26.7%)	0.0643	KEGG
Activation of the mRNA upon binding of the cap-binding complex and eIFs, and subsequent binding to 43S	0.00414	69	13 (20.3%)	0.0643	Reactome
B cell receptor signaling	0.00418	134	22 (16.4%)	0.0643	INOH
Doxorubicin Pathway (Cardiomyocyte Cell), Pharmacodynamics	0.00419	24	7 (29.2%)	0.0643	PharmGKB
proteasome complex	0.00419	24	7 (29.2%)	0.0643	BioCarta
UCH proteinases	0.0042	102	18 (17.6%)	0.0643	Reactome
Translation Factors	0.00428	50	11 (22.0%)	0.0651	Wikipathways
MyD88 cascade initiated on plasma membrane	0.00446	87	16 (18.4%)	0.0666	Reactome
Toll Like Receptor 10 (TLR10) Cascade	0.00446	87	16 (18.4%)	0.0666	Reactome
Toll Like Receptor 5 (TLR5) Cascade	0.00446	87	16 (18.4%)	0.0666	Reactome
SRP-dependent cotranslational protein targeting to membrane	0.00469	124	20 (16.8%)	0.0693	Reactome
L13a-mediated translational silencing of Ceruloplasmin expression	0.00469	125	20 (16.8%)	0.0693	Reactome
Signaling by ERBB4	0.00496	44	10 (22.7%)	0.0728	Reactome
TRAF6 mediated induction of NFkB and MAP kinases upon TLR7/8 or 9 activation	0.005	88	16 (18.2%)	0.0731	Reactome
Nucleotide Metabolism	0.00516	19	6 (31.6%)	0.0742	Wikipathways
Listeria monocytogenes entry into host cells	0.00516	19	6 (31.6%)	0.0742	Reactome
GTP hydrolysis and joining of the 60S ribosomal subunit	0.00517	126	20 (16.7%)	0.0742	Reactome
RNA Polymerase II Transcription	0.00526	1236	136 (11.1%)	0.0751	Reactome
Propanoate metabolism	0.00537	25	7 (28.0%)	0.0755	INOH
gluconeogenesis	0.00537	26	7 (28.0%)	0.0755	HumanCyc
Saturated fatty acids beta-oxidation	0.00537	25	7 (28.0%)	0.0755	EHMN
Selenium Metabolism and Selenoproteins	0.00566	38	9 (23.7%)	0.0788	Wikipathways
RNA transport - Homo sapiens (human)	0.00567	171	26 (15.2%)	0.0788	KEGG
Transfer of Acetyl Groups into Mitochondria	0.00573	9	4 (44.4%)	0.0792	SMPDB
Gene expression (Transcription)	0.0059	1373	149 (10.9%)	0.0794	Reactome
SHC1 events in ERBB4 signaling	0.00593	14	5 (35.7%)	0.0794	Reactome
Malonyl-coa decarboxylase deficiency	0.00593	14	5 (35.7%)	0.0794	SMPDB
Malonic Aciduria	0.00593	14	5 (35.7%)	0.0794	SMPDB
Propanoate Metabolism	0.00593	14	5 (35.7%)	0.0794	SMPDB
Methylmalonic Aciduria Due to Cobalamin-Related Disorders	0.00593	14	5 (35.7%)	0.0794	SMPDB
MyD88 dependent cascade initiated on endosome	0.00626	90	16 (17.8%)	0.0815	Reactome
Toll Like Receptor 7/8 (TLR7/8) Cascade	0.00626	90	16 (17.8%)	0.0815	Reactome

Valine Leucine Isoleucine degradation	0.00632	32	8 (25.0%)	0.0815	INOH
ROS, RNS production in phagocytes	0.00632	32	8 (25.0%)	0.0815	Reactome
Propanoate metabolism - Homo sapiens (human)	0.00632	32	8 (25.0%)	0.0815	KEGG
Beta oxidation of hexanoyl-CoA to butanoyl-CoA	0.00637	5	3 (60.0%)	0.0815	Reactome
Beta oxidation of lauroyl-CoA to decanoyl-CoA-CoA	0.00637	5	3 (60.0%)	0.0815	Reactome
Beta oxidation of octanoyl-CoA to hexanoyl-CoA	0.00637	5	3 (60.0%)	0.0815	Reactome
malate-aspartate shuttle	0.00637	5	3 (60.0%)	0.0815	HumanCyc
Antigen processing: Ubiquitination & Proteasome degradation	0.00667	264	36 (13.8%)	0.0849	Reactome
superpathway of methionine degradation	0.00682	20	6 (30.0%)	0.0861	HumanCyc
Selenium Micronutrient Network	0.00684	83	15 (18.1%)	0.0861	Wikipathways
Retrograde endocannabinoid signaling - Homo sapiens (human)	0.00686	148	23 (15.5%)	0.0861	KEGG
Insulin Pathway	0.00691	46	10 (21.7%)	0.0863	PID
Cellular response to heat stress	0.00703	99	17 (17.2%)	0.0874	Reactome
Epithelial cell signaling in Helicobacter pylori infection - Homo sapiens (human)	0.00707	68	13 (19.1%)	0.0875	KEGG
Generic Transcription Pathway	0.00744	1107	122 (11.1%)	0.0915	Reactome
Golgi-to-ER retrograde transport	0.00763	116	19 (16.4%)	0.0934	Reactome
Cooperation of Prefoldin and TriC/CCT in actin and tubulin folding	0.0077	33	8 (24.2%)	0.0934	Reactome
bioactive peptide induced signaling pathway	0.0077	33	8 (24.2%)	0.0934	BioCarta
HDR through Homologous Recombination (HR) or Single Strand Annealing (SSA)	0.00795	135	21 (15.8%)	0.096	Reactome
mTOR signaling pathway - Homo sapiens (human)	0.00806	151	23 (15.3%)	0.096	KEGG
superpathway of conversion of glucose to acetyl CoA and entry into the TCA cycle	0.00809	48	10 (21.3%)	0.096	HumanCyc
formaldehyde oxidation	0.00813	2	2 (100.0%)	0.096	HumanCyc
Abasic sugar-phosphate removal via the single-nucleotide replacement pathway	0.00813	2	2 (100.0%)	0.096	Reactome
Insulin effects increased synthesis of Xylulose-5-Phosphate	0.00813	2	2 (100.0%)	0.096	Reactome
GLI3 is processed to GLI3R by the proteasome	0.00824	15	5 (33.3%)	0.096	Reactome
Degradation of GLI2 by the proteasome	0.00824	15	5 (33.3%)	0.096	Reactome
Degradation of GLI1 by the proteasome	0.00824	16	5 (33.3%)	0.096	Reactome
Formation of a pool of free 40S subunits	0.00851	115	18 (16.5%)	0.0986	Reactome
Signaling by Receptor Tyrosine Kinases	0.00868	423	53 (12.5%)	0.1	Reactome
Mono-unsaturated fatty acid beta-oxidation	0.00883	21	6 (28.6%)	0.101	EHMN
Folding of actin by CCT/TriC	0.00888	10	4 (40.0%)	0.102	Reactome
Class I MHC mediated antigen processing & presentation	0.00901	324	42 (13.1%)	0.102	Reactome
Intra-Golgi and retrograde Golgi-to-ER traffic	0.00905	186	27 (14.5%)	0.102	Reactome
Regulation of insulin secretion	0.00905	70	13 (18.6%)	0.102	Reactome
AndrogenReceptor	0.00907	143	22 (15.4%)	0.102	NetPath
Translation initiation complex formation	0.00975	68	12 (19.0%)	0.109	Reactome
Ribosomal scanning and start codon recognition	0.00975	68	12 (19.0%)	0.109	Reactome

Supplemental Table 9: Sequences of oligonucleotides used for real time PCR.

Gene name	Accession number	Forward sequence (5'-3')	Reverse sequence (5'-3')
PCNA	NM_182649.2	CAAGTAATGTCGATAAAGAGGAGG	GTGTCACCGTTGAAGAGAGTGG
NGN3	NM_020999.4	CCTAAGAGCGAGTTGGCACTGA	AGTGCCGAGTTGAGGTTGTGCA
PDX1	NM_000209.4	GAAGTCTACCAAAGCTCACGCG	GGAACTCCTTCTCCAGCTCTAG
NKX2.2	NM_002509.4	CCTTCTACGACAGCAGCGACAA	ACTTGGAGCTTGAGTCCTGAGG
PAX6	NM_001368894.2	CTGAGGAATCAGAGAAGACAGGC	ATGGAGCCAGATGTGAAGGAGG
SCLY	NM_016510.7	ACTTCCTCGGTGGAACACGACT	ATGATGGTCACGAGGCGTGTGG
SRPRA	NM_003139.4	TCGTCACCTTCTGCGGCGTTAA	CCAGCACGAAATGTATCACAGGC
SEC61A2	NM_018144.4	TGCCACCAACATCTGTGAGACC	CCTCCCGTAAAGCTCGGACTTT
TBP	NM_003194.5	TGTATCCACAGTGAATCTTGGTTG	GGTTCGTGGCTCTCTTATCCTC
